# Pathobiochemical signatures of cholestatic liver disease in bile duct ligated mice

**DOI:** 10.1186/s12918-015-0229-0

**Published:** 2015-11-20

**Authors:** Kerstin Abshagen, Matthias König, Andreas Hoppe, Isabell Müller, Matthias Ebert, Honglei Weng, Herrmann-Georg Holzhütter, Ulrich M. Zanger, Johannes Bode, Brigitte Vollmar, Maria Thomas, Steven Dooley

**Affiliations:** Institute for Experimental Surgery, University Medicine Rostock, Schillingallee 69a, Rostock, 18057 Germany; Institute for Biochemistry, Computational Systems Biochemistry Group, Charité University Medicine Berlin, Berlin, 10117 Germany; Dr. Margarete Fischer-Bosch Institute of Clinical Pharmacology, University of Tuebingen, Tuebingen, Stuttgart 70376 Germany; Department of Medicine II, Section Molecular Hepatology, Medical Faculty Mannheim, Heidelberg University, Mannheim, 68167 Germany; Department for Gastroenterology, Hepatology and Infectiology, Heinrich-Heine University of Düsseldorf, Düsseldorf, 40225 Germany; Department of Medicine II, Medical Faculty Mannheim, Heidelberg University, Mannheim, 68167 Germany

**Keywords:** Liver injury, Mouse, Systems biology, Fibrosis, Cell proliferation, Bile duct ligation, Cholestasis, Morphological profiling, Virtual liver network

## Abstract

**Background:**

Disrupted bile secretion leads to liver damage characterized by inflammation, fibrosis, eventually cirrhosis, and hepatocellular cancer. As obstructive cholestasis often progresses insidiously, markers for the diagnosis and staging of the disease are urgently needed. To this end, we compiled a comprehensive data set of serum markers, histological parameters and transcript profiles at 8 time points of disease progression after bile duct ligation (BDL) in mice, aiming at identifying a set of parameters that could be used as robust biomarkers for transition of different disease progression phases.

**Results:**

Statistical analysis of the more than 6,000 data points revealed distinct temporal phases of disease. Time course correlation analysis of biochemical, histochemical and mRNA transcript parameters (=factors) defined 6 clusters for different phases of disease progression. The number of CTGF-positive cells provided the most reliable overall measure for disease progression at histological level, bilirubin at biochemical level, and metalloproteinase inhibitor 1 (Timp1) at transcript level. Prominent molecular events exhibited by strong transcript peaks are found for the transcriptional regulator Nr0b2 (Shp) and 1,25-dihydroxyvitamin D(3) 24-hydroxylase (Cyp24a1) at 6 h. Based on these clusters, we constructed a decision tree of factor combinations potentially useful as markers for different time intervals of disease progression. Best prediction for onset of disease is achieved by fibronectin (Fn1), for early disease phase by Cytochrome P450 1A2 (Cyp1a2), passage to perpetuation phase by collagen1α-1 (Col1a1), and transition to the progression phase by interleukin 17-a (Il17a), with early and late progression separated by Col1a1. Notably, these predictions remained stable even for randomly chosen small sub-sets of factors selected from the clusters.

**Conclusion:**

Our detailed time-resolved explorative study of liver homogenates following BDL revealed a well-coordinated response, resulting in disease phase dependent parameter modulations at morphological, biochemical, metabolic and gene expression levels. Interestingly, a small set of selected parameters can be used as diagnostic markers to predict disease stages in mice with cholestatic liver disease.

**Electronic supplementary material:**

The online version of this article (doi:10.1186/s12918-015-0229-0) contains supplementary material, which is available to authorized users.

## Background

Cholestatic liver diseases are caused by an impaired flow of bile from liver to duodenum. A major component of bile are the bile salts, strong detergents required for extraction of lipids from apical membrane of hepatocytes into bile fluid and emulgation of lipids in the gut. Moreover, bile fluid comprises numerous endogenous products (e.g. bilirubin) and potentially toxic compounds resulting from the clearance function of the liver. Hence, accumulation of bile compounds due to cholestasis causes unspecific cellular damage that initiates a cascade of inflammatory and fibrogenic events in the liver. At the cellular and molecular level, these comprise, among others, necrosis of hepatocytes and cholangiocytes, activation of macrophages, releasing pro-inflammatory cytokines and chemokines, neutrophil infiltration, cholangiocyte and hepatocyte proliferation, stellate cell activation with progressive fibrosis causing secondary biliary cirrhosis, and ultimately liver failure or progression into liver cancer [1].

Multiple pathologies may represent the primary trigger of impaired bile flow, e.g. defects in export of bile from hepatocytes to bile canaliculi (hepatocellular cholestasis), obstruction of bile ducts by gall stones or local tumor impingement (extrahepatic cholestasis) [2]. Among the most common cholestatic liver diseases in the adult population are primary biliary cirrhosis (PBC) and primary sclerosing cholangitis (PSC), while biliary atresia and Alagille syndrome are found in the pediatric population [3–5]. The corresponding experimental model to induce obstructive cholestatic injury in mice and rats is surgical bile duct ligation (BDL) [6, 7], which results in stereotypical histopathological phenotypes as in human cholestasis. The BDL experimental model has been well described and evaluated in rats and mice and is widely used to study cholestatic liver injury and fibrogenesis [8].

Chronic liver diseases (CLD), like cholestasis, represent with characteristic temporal morphologic, biochemical and molecular changes in liver and serum. For instance, in the BDL model, an early phase of acute hepatocyte injury is followed by a proliferative response of different parenchymal and non-parenchymal liver cell types, up-regulation of pro-inflammatory and pro-fibrotic cytokines and metabolic enzymes, presenting as liver fibrosis after around 7 days [7, 8]. Such alteration dynamics can be exploited to identify biomarkers of specific stages of disease progression and disease severity. Hitherto, semi-quantitative morphological scoring is the standard technique for grading and staging a CLD. However, the availability of high-throughput technologies enables to flank the histological assessment of injured tissue with a comprehensive molecular profiling of gene transcripts, gene products (proteins) and metabolites, in liver tissue as well as in serum of patients and animal models. Such analyses not only will provide a more detailed characterization and thus more refined staging of disease progression, they also lead to a deeper understanding of the molecular networks governing histological and pathophysical alterations observed at higher scales of tissue organization. The identification of key processes triggering the transition between different phases of disease progression based on high (or intermediate) throughput experimental data from different levels of cellular organization requires mathematical analyses, which take into account multiple parallel processes and process dynamics.

In this study, we wanted to systemize existing and newly acquired knowledge on morphological, biochemical and molecular biomarkers of cholestasis, and analyze disease progression following BDL in mice in a time resolved comprehensive manner. Our approach sets itself apart from existing studies, which either describe the time course of a limited number of selected parameters after BDL [7, 8], or provide gene expression signatures for a limited number of time points, thereby missing the acute damage after BDL in the first 24 h and long-term effects after 7 days [9]. None of these preceding studies applied predictive models based on acquired time course data. Therefore, a central aim of our study was to identify molecular markers for temporal progression of BDL cholestasis by correlating high-accuracy image data and transcriptional profiles of a set of preselected targets with pathobiochemical markers of obstructive cholestasis. We collected from 8 different time points after BDL more than 6,000 experimental data points (Additional file 1), comprising immunohistochemistry, biochemistry and molecular profiling measures. Statistical methods were applied to unravel robust interrelations in this large-scale data set, and to find clusters of parameters corresponding to characteristic time profiles of disease onset/progression. We correlated level and timing of pathophysiological events with transcriptional changes, in order to define molecular markers, and developed predictive decision trees on a subset of biomarkers for the assessment of different disease phases as they occur during development of cholestasis.

## Methods

### Ethic statement

All experiments were approved by the local government Landesamt für Landwirtschaft, Lebensmittelsicherheit und Fischerei Mecklenburg-Voprommern (LALLF M-V/TSD/7221.3-1.2-049/09) and performed in accordance with the German legislation on protection of animals and the National Institutes of “Health Guide for the Care and Use of Laboratory Animals” (Institute of Laboratory Animal Resources, National Research Council; NIH publication 86–23 revised 1985).

### Mice

Male C57BL/6 J (Charles River Laboratories, Sulzfeld, Germany) at 8–10 weeks of age with a body weight of 23–26 g were kept on water and standard laboratory chow ad libitum.

### Surgical procedure and experimental groups

Mice were anesthetized by breathing isoflurane (1.5 vol%). BDL was performed after midline laparotomy. The common bile duct was ligated three times with 5–0 silk and transected between the two most distal ligations. Sham operation was performed similarly, except for ligation and transection of the bile duct (0 h, n = 5). All surgical procedures were performed under aseptic conditions. Animals were allowed to recover from anesthesia and surgery under a red warming lamp and were held in single cages until subsequent experiments followed at postoperative hours 6, 12, 18 and 30 (n = 5 animals per time point), and at 2, 5 and 14 days after BDL (n = 5 animals per time point). Sham-operated animals without BDL served as controls (n = 5). To analyze the regenerative response in regard to proliferation of different cell types, 5-bromo-2-deoxyuridine (BrdU; 50 mg/kg bw ip) was injected 1 h prior to harvest of liver tissue. BrdU incorporation into DNA was analyzed by immunohistochemistry. To obtain blood and liver samples, mice were killed at the indicated time points. Liver tissue was partially embedded in paraffin for morphology analysis and snap frozen for molecular biology and biochemistry analyses. In addition, liver tissue served for the parallel Taqman qRT-PCR using microfluidic Fluidigm Biomark™ platform (Fluidigm, CA, USA).

### Hematological measurements and plasma enzyme levels

Animals were anesthetized and exsanguinated by puncture of the vena cava inferior. Red blood cell and blood platelets count, hemoglobin, and hematocrit were assessed with an automated cell counter (Sysmex KX-21, Sysmex). Plasma activities of alanine aminotransferase (ALT), aspartate aminotransferase (AST) and glutamate dehydrogenase (GLDH) were measured spectrophotometrically.

### Assays

EDTA plasma served for the analysis of albumin as a parameter of liver function, which was determined with a commercially available enzyme-linked immunosorbent assay kit in accordance with the manufacturer’s instructions (Assaypro, MO, USA).

### Histopathology and image analysis

Liver tissue samples were fixed in formalin for 2 to 3 days and embedded in paraffin. 5 μm sections were stained with hematoxylin and eosin (H&E) for routine examination and quantification of bile infarcts. Sirius red staining served for quantification of collagen deposition. All samples from a series of experiments were stained simultaneously and evaluated in a blinded manner. For histomorphometric analysis, images of 20 random low power fields (x10 magnification, Olympus BX 51, Hamburg, Germany) were acquired with a Color View II FW camera (Color View, Munich, Germany) and evaluated using an image analysis system (Adobe Photoshop, Adobe Systems, Uxbridge, UK). Fibrosis deposition was quantified as a percentage of Sirius red stained area compared with the total section area. The surfaces of large centrilobular veins and large portal tracts were excluded from this calculation. Bile infarcts were quantified in H&E-stained sections in a similar manner and the percentage of the focal necrosis surface to the whole liver section area was assessed.

### Immunohistochemistry and image analysis

For analyzing DNA-incorporated BrdU in liver cells, 4 μm sections collected on poly-L-lysine-coated glass slides were incubated with monoclonal mouse anti-BrdU antibody (1:50; Dako Cytomation, Hamburg, Germany) overnight at 4 °C, followed by horseradish-peroxidase (HRP)-conjugated goat anti-mouse immunoglobin (LSAB kit plus; Dako). Sites of peroxidase-binding were detected by 3,3’-diaminobenzidine (Dako). Sections were counterstained with hemalaun. BrdU-positive hepatocellular nuclei were counted in a blinded manner within 30 consecutive high power fields (HPF) (x40 objective, numerical aperture 0.65) and are given as cells/mm^2^. In analogy, BrdU-expressing non-parenchymal cells were assessed and also given as cells/mm^2^.

To specify the proliferative response of non-parenchymal cells upon BDL, we performed double immunohistochemistry of BrdU with specific markers for different liver cells: F4-80/BrdU for Kupffer cells and SM22α/BrdU for biliary epithelial cells (BECs). For each protocol, the immune-staining procedure for the specific marker was conducted after the BrdU staining protocol. Resident liver macrophages were analyzed using the F4-80 antigen (1:10; Serotec, Oxford, UK). Overnight (ON) incubation (4 °C) with the first antibody (polyclonal rat anti-F4-80) was followed by alkaline-phosphatase (AP) conjugated mouse anti-rat immunoglobulin (1:200; Santa Cruz Biotechnology, Santa Cruz, CA, USA). The sites of AP-binding were detected using the chromogen fuchsin (Dako).

BECs and oval cells were detected by ON incubation (4 °C) with a polyclonal rabbit anti-SM22α antibody (1:100; Abcam, Cambridge, UK) followed by AP conjugated goat anti-rabbit immunoglobulin as secondary antibody (1:100; Dako). The sites of AP-binding were detected by Permanent Red (Dako).

Slides were viewed under a light microscope (Olympus BX 51) and the number of BrdU-positive cells co-expressing F4-80 or SM22α were counted in a blinded manner within 30 consecutive high power fields (HPF) (x40 objective, numerical aperture 0.65) and are given as cells/mm^2^.

Antibodies for detection of α-SMA in HSCs and of S100a4-positive cells were from DAKO (M0851 and A5114, 1:500 and 1:200 dilution, respectively). CTGF antibody was from Santa Cruz (sc-1439, 1:200 dilution). Sections were de-paraffinized in serial ethanol dilutions. After a PBS wash, sections were transferred into 10 mM sodium citrate buffer (pH 6.0) and antigen unmasking was performed in a microwave. After cooling down, sections were incubated in peroxidase blocking reagent (Dako) for 1 h and with primary antibodies ON at 4 °C. EnVision peroxidase (Dako) was applied for 1 h at room temperature after a PBS wash next day. Sections were developed with diaminobenzidine for 5 min. The number of α-SMA-, CTGF- and S100a4-positive cells was quantified under a Leica light microscope (x20) by counting three fields.

### High-throughput quantitative Taqman RT-PCR analysis

Total RNA was isolated from the liver tissue samples using RNeasy Mini Kit including on column genomic DNA digestion with RNase free DNase Set (Qiagen, Hilden, Germany). RNA was reverse transcribed to cDNA with TaqMan Reverse Transcription Reagents (Applera GmbH, Darmstadt, Germany). For quantitative real-time PCR, we used the Fluidigm’s Biomark high-throughput qPCR chip platform (Fluidigm Corporation, San Francisco, CA, USA) with pre-designed gene expression assays from Applied Biosystems, according to the manufacturer’s instructions [10]. Data were analyzed using the ddCt method and expression values were normalized to the expression levels of the Gapdh gene.

### Statistical data analysis

#### Dimension reduction

A one-way analysis of variance (ANOVA) was applied to reduce the data set to the parameter subset showing significant (p_adj_ < 0.05) up- or down-regulation during the time course. Multiple testing correction was performed using the Holm procedure [11]. To specifically test for the initial changes, a two-tailed unpaired *t*-test (Welch correction for nonhomogeneity of variance) was performed for all factors between the classes 0 h and 6 h.

#### Correlation analysis

Correlation between two factor time courses was calculated via Y^S3^, a modified correlation coefficient based similarity measure for clustering time-course gene expression data [12]. The correlation Y_i,j_^S3^ between two factors i and j is the linear combination of three terms: i) a classical correlation part based on Spearman correlation ${\mathrm{S}}_{\mathrm{i},\mathrm{j}}^{*},\kern0.5em \mathsf{i}\mathsf{i}\Big)\kern0.5em {{\mathrm{A}}_{\mathrm{i},}^{*}}_{\mathrm{j}}^{*}$ accounting for the similarity in changes between the two time courses, and iii) M_i,j_^*^ comparing the location of the minimum and maximum values of the time course (see [12] and Additional file 2, correlation analysis for definitions). S_i,j_^*^ is calculated on individual data points, A_i,_^*^_j_^*^ and M_i,j_^*^ on the mean time courses averaged over the repeats per time point. For the calculation of the similarity of changes within time courses, A_i,_^*^_j_^*^, we used Spearman (S) correlation which is more robust against outliers as Pearson (R) correlation:

$$ \begin{array}{l}{A_{i,}^{*}}_j^{*}\kern0.5em =\kern0.5em \left(S\left({d}_i,\kern0.5em {d}_j\right)\kern0.5em +\kern0.5em 1\right)/2\\ {}{Y_{i,}^S}_j^3\kern0.5em =\kern0.5em {\omega}_1{S}_{\mathrm{i},\mathrm{j}}^{*}\kern0.5em +\kern0.5em {\omega}_2{A_{i,}^{*}}_j^{*}\kern0.5em +{\omega}_3{M}_{\mathrm{i},\mathrm{j}}^{*}\end{array} $$

In the analysis, the following weights were used: *ω*_1_ = 0.5, *ω*_2_ = 0.3, *ω*_3_ = 0.2. All reported correlations are ${{\overline{Y}}_i^S}_{,j}^3$ values in the interval [−1,1]:

$$ {{\overline{Y}}_i^S}_{,j}^3\kern0.5em =\kern0.5em 2\left({{\overline{Y}}_i^S}_{,j}^3\kern0.5em -\kern0.5em 0.5\right) $$

Cluster analysis of the correlation matrix used complete-linkage hierarchical clustering with Euclidian distance measurement. This combination of complete-linkage with *Y*^*s*^ provided the best enrichments on gene expression time series in a recent comparison of methods [13, 14]. The number of clusters *N*_*c*_ = 6, was selected as maximum number of clusters so that all clusters contain more than one factor. Normalization of factors was performed separately for each factor *f*_*k*_ for all time points *i* = 1, … , *N*_*t*_ and repeats *r* = 1, … , *N*_*r*_ with *N*_*t*_ = 8 and *N*_*r*_ = 5 via

$$ {\overline{f}}_k\left({t}_{i,r}\right)\kern0.5em =\kern0.5em \frac{\left({f}_k\left({t}_{i,r}\right)-\kern0.5em <\kern0.5em {f}_k\kern0.5em >\right)}{ \max \left({f}_k\right)\kern0.5em -\kern0.5em  min\kern0.5em \left({f}_k\right)} $$

#### Decision trees

For the prediction of distinct time points of disease progression, a regression tree with the mean normalized factor data of the 6 clusters as predictor variables and the log transformed time points ${\tilde{t}}_i$ as dependent variable was fitted based on recursive partitioning using *rpart* [15]. Logarithmic transformation was performed to obtain approximately equal distant time points.

$$ {\tilde{t}}_i\kern0.5em =\kern0.5em  \log \kern0.5em \left({t}_i\kern0.5em +\kern0.5em 1\right) $$

The regression tree was fitted using the complete training set (*N*_*s*_ = *N*_*t*_*N*_*r*_ = 40) with the minimum number of observations in a node, for which a split was computed being 6, the minimum number of observations in a terminal node as 2, and the complexity parameter *c*_*p*_ = 0.01. The splitting criterion, deciding which predictor variable gives the best split for nodes in the regression tree was *S*_*T*_ − (*S*_*L*_ + *S*_*R*_), with ${S}_T\kern0.5em =\kern0.5em \varSigma {\left({\tilde{t}}_{\iota}\kern0.5em -\kern0.5em <\tilde{t}>\right)}^2$ the sum of squares for node T, and *S*_*T*_ and *S*_*L*_ the sums of squares for the left and right child. A leave one out approach was used to test the robustness of the predicted time classes and predictive performance: For each sample (*N*_*S*_ = 40 mice), the regression tree was generated under the exclusion of data from the sample with subsequent prediction on the left out test data (see Additional file 2, decision trees).

The predictive capacity of the regression tree was evaluated using all single combinations of individual factors from the clusters (88572) and a random subset of 10000 two factor combinations from each cluster. Predictions for a given combination of factors 〈*f*_1_〉, … , 〈*f*_6_〉 from the 6 clusters were scored, using the root mean square distance on log scale *d*, with the best combination of factors minimizing d.

$$ d\left(\left\langle {f}_1\right\rangle, \kern0.5em \dots \kern0.5em ,\kern0.5em \left\langle {f}_6\right\rangle \right)\kern0.5em =\kern0.5em \frac{1}{N_S}\sqrt{\underset{i=1}{\overset{N_S}{\Sigma}}{\left({t}_i^{pre}-\kern0.5em {t}_i^{exp}\right)}^2} $$

All computations were performed in R with source code data and the full analysis available from http://matthiaskoenig.github.io/bdl-analysis (doi:10.5281/zenodo.32577) and Additional file 2.

## Results and discussion

### Temporal changes of biochemical, cellular and histochemical markers after BDL

**Fig. 1:**
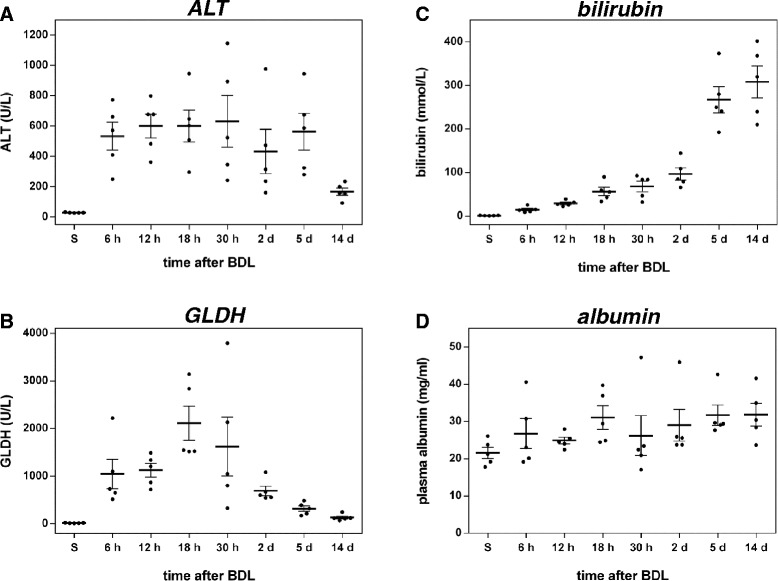
Analysis of liver injury and function. Plasma activities of alanine aminotransferase (ALT) (**a**) and glutamate dehydrogenase (GLDH) (**b**) and concentrations of plasma bilirubin (**c**) and albumin (**d**) at multiple time points after BDL. Values are given in means ± SEM of five independent experiments per time point

In mice, BDL over 14 days induces time dependently progressing stages of a secondary biliary CLD. The first week begins with an acute cholestatic injury, associated with necroinflammation that is followed by a chronic injury stage, comprising hepatitis and liver fibrosis. Macroscopically, marked dilation of the gallbladder and formation of bilioma are found, in line with weight loss and a mortality rate of 10 % in the first week due to bile leakage and rupture of the gallbladder [16]. Pathophysiologically, BDL disturbs glandular liver function and hepatobiliary transport, comprising detoxification and secretion functions, including bile formation. Obstruction of the bile duct leads to afflux of newly generated bile fluid. The main components of the bile, bile acids and phospholipids, induce toxicity and damage towards hepatocytes and cholangiocytes, therewith initiating the disease process. Rapidly after BDL, mice develop obstructive jaundice and cholestasis, as displayed by markedly elevated serum transaminase activity and bilirubin levels (Fig. 1), macroscopically evident from yellow ears and urine. Within the first 30 h, there is a massive release of liver enzymes, like ALT and GLDH, reflecting hepatocyte damage as initial pathophysiological event in the process of BDL-induced liver fibrosis (Fig. 1a, b).

Plasma levels of diagnostic liver enzymes remain elevated over several days, but then drop to reach values that were only slightly above those of sham operated mice until day 14. Concomitantly, liver detoxification capacity is deteriorated, as indicated by the rise of total bilirubin, a classical plasma marker of cholestasis (Fig. 1c). Notably, the plasma level of albumin, an important parameter for the evaluation of liver function remains relatively constant over the time course of 14 days (Fig. 1d). The systemic blood cell count (Table 1) shows constant levels of erythrocytes and platelets up to day 5. In contrast, leukocytes decrease by 50 % during the first two days, reflecting intrahepatic cell entrapment, and recover to values of sham operated animals within the subsequent observation period. During progression of fibrosis, red blood cells, hemoglobin and hematocrit are slightly decreasing.

**Table 1:** Systemic blood cell count of sham-operated mice (S) and mice, which underwent BDL. Values are given as means ± SEM

	Erythrocytes [*10^12^/L]	Platelets [*10^9^/L]	Leukocytes [*10^9^/L]	Hemoglobin [mmol/L]	Hematocrit
S	8.4 ± 0.1	1177 ± 60	7.5 ± 0.3	7.9 ± 0.1	44.8 ± 0.7
6 h	8.1 ± 0.1	1061 ± 39	4.2 ± 0.5	7.7 ± 0.1	42.8 ± 0.7
12 h	8.2 ± 0.1	1036 ± 47	4.5 ± 0.4	7.7 ± 0.1	43.0 ± 0.6
18 h	8.7 ± 0.3	856 ± 110	4.1 ± 0.2	8.1 ± 0.3	45.6 ± 1.9
30 h	8.5 ± 0.5	1071 ± 100	5.8 ± 0.9	7.9 ± 0.5	44.9 ± 2.8
2 d	8.7 ± 0.2	1117 ± 65	4.7 ± 1.3	6.5 ± 1.7	45.9 ± 0.9
5 d	8.7 ± 0.3	1295 ± 107	7.6 ± 1.2	7.8 ± 0.3	46.5 ± 1.6
14 d	7.6 ± 1.4	1362 ± 58	7.4 ± 1.1	6.6 ± 0.2	38.4 ± 1.3

**Fig. 2:**
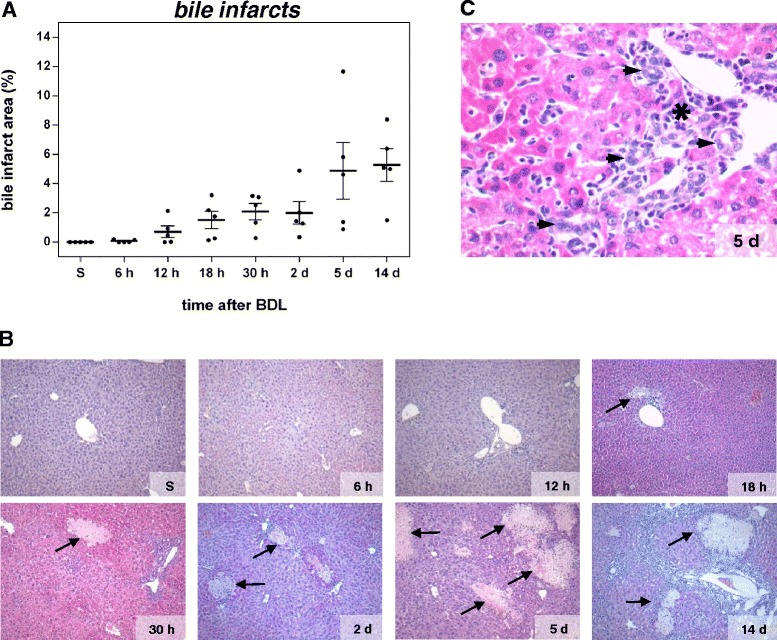
Quantification of bile infarcts in H&E stained liver sections at multiple time points after BDL (**a**). Values are given in means ± SEM of five independent experiments per time point. Representative H&E stainings of paraffin embedded liver sections for each time point after BDL (**b**; arrows indicate bile lakes; magnification x10) with higher magnifications (x40) in (**c**), displaying cellular infiltrates (asterisk) and formation of artificial bile ductules (arrowhead)

**Fig. 3:**
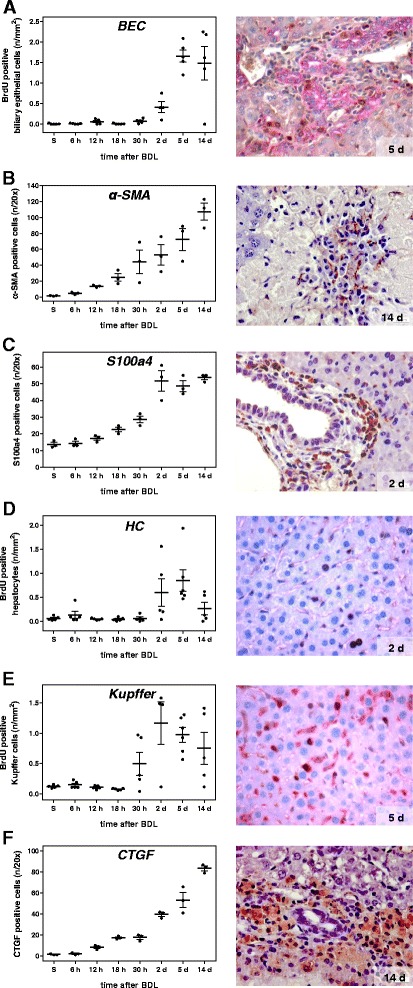
Analysis of the proliferative and cellular response at multiple time points after BDL. Quantitative immunohistochemical analysis of BrdU positive biliary epithelial cells (**a**), liver cells positive for α-SMA (**b**) and S100a4 (**c**), BrdU positive hepatocytes (**d**) and Kupffer cells (**e**) and CTGF positive cells (**f**). Values are given in means ± SEM of five independent experiments per time point. Corresponding representative immunohistochemical stainings are shown in the right panel (magnifications x40)

In consequence of intrahepatic toxic bile accumulation, progressive development of confluent bile lakes is a hallmark of cholestasis. Histological quantification of bile infarcts, defined as clusters of injured hepatocytes, reveals a steady rise of infarct areas until day 14 after BDL (Fig. 2a). The typical appearance of liver tissue at representative time points after BDL using H&E staining is depicted in Fig. 2b. Further histopathological changes of the livers after BDL include enlargement of portal tracts, dilation of bile canaliculi as well as proliferation of BECs and oval cells (Fig. 3a), resulting in formation of artificial bile ductules (Fig. 2c), a cellular response termed ‘ductular reaction’ [7, 17]. Recent data from lineage tracing experiments indicate however that BECs and oval cells do not contribute to the population of ECM producing/fibrogenic cells, which in the BDL model is largely consisting of hepatic stellate cells (HSCs) [18].

**Fig. 4:**
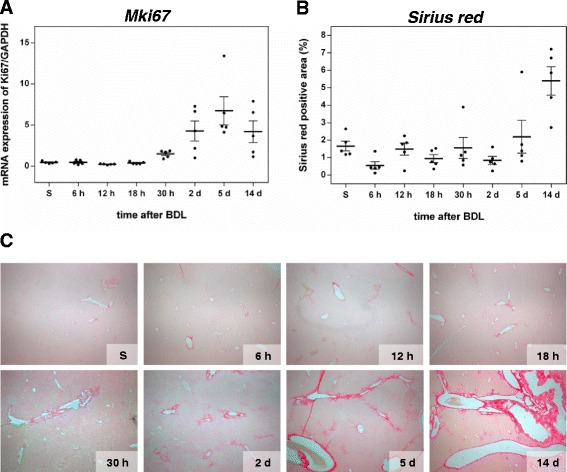
Analysis of proliferation and extracellular matrix accumulation. mRNA quantification of the proliferation marker Ki67 (**a**) by Fluidigm real-time PCR. Values are given in means ± SEM of five independent experiments per time point. Quantitative analysis of extracellular matrix deposition (**b**) and representative histological images (**c**; magnification x10) of Sirius red positive areas at multiple time points after BDL. Values are given in means ± SEM of five independent experiments per time point

The inflammatory response resulting from chronic hepatocyte injury is reflected by accumulation of immune cells in the liver, among others, T cells, macrophages and dendritic cells, which are mainly found within and around bile infarct areas (Fig. 2c, asterisk) [19]. It is initiated by resident liver cells, primarily liver macrophages (Kupffer cells, KC) and activated HSCs, both secreting a wide range of cytokines and chemokines, which determine quality and quantity of inflammatory and consequently fibrotic responses [20, 21]. Upon parenchymal damage, quiescent HSCs undergo a phenotypical change to myofibroblasts (MFBs). The most prominent role of MFBs is gain of a migratory phenotype and extracellular matrix (ECM) production and reorganization, as reflected by, among others, increased synthesis of α-SMA, type I collagen and TIMPs. A marked increase of the number of α-SMA- and S100a4-positive cells, as measured by immunohistochemistry, is observed after BDL (Fig. 3b, c). Migration of MFBs to the site of injury and their contractility contribute to liver scarring and portal hypertension. This is accompanied by parenchymal cell proliferation, which begins at day 2 as regenerative response and which decreases at day 14 (Fig. 3d). With a slightly faster response as compared to HSCs, KCs start to proliferate at the 30 h time point upon BDL (Fig. 3e). The overall hepatic proliferative response as analyzed by immunohistochemistry is confirmed by elevated mRNA expression of Ki67 (Fig. 4a).

Between day 5 and 14 after BDL, periportal alterations are associated with fibrotic changes. As demonstrated by Sirius red staining, extensive fibrosis, characterized by a several-fold increase of collagen deposition (Fig. 4b), including bridging, occurs at day 5 after BDL (Fig. 4c). We further stained for connective tissue growth factor (CTGF), a prominent fibrogenic cytokine and enhancer of TGF-β effects [22]. Appearance of CTGF-positive cells starts as early as 12 h upon BDL and their count increases continuously (Fig. 3f).

### Time phases of disease progression after BDL

**Fig. 5:**
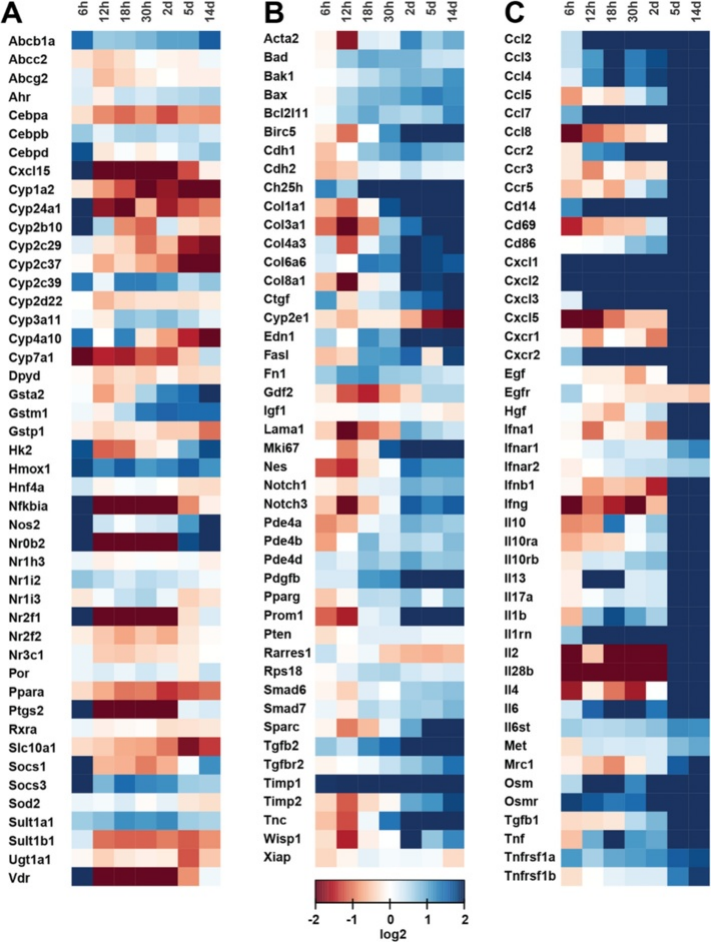
Heat maps displaying gene expression pattern at multiple time points after BDL. Gene expression relative to the Gapdh gene, obtained from Fluidigm qPCR, are shown as fold changes to sham operated mice (0 h) and are displayed in log2 scale. Red colour indicates down-regulation (log2 of 2), blue up-regulation (log2 of −2) and white transcription fold changes about 1 (log2 of 0). **a** selected ADME genes, (**b**) selected fibrogenesis genes, and (**c**) selected inflammation genes

To define distinct disease progression phases upon BDL damage, time-resolved transcriptomics profiles of three preselected gene panels relating to (1) hepatocyte metabolism, (2) fibrogenesis, and (3) inflammation were measured using the Fluidigm platform (Fig. 5, Additional file 2 explorative data analysis) and matched with biochemical and histological markers. Selection of representative genes for (1) ADME- (absorption, distribution, metabolism, and excretion) (Fig. 5a) (2) fibrogenesis- (Fig. 5b), and (3) inflammation-related genes (Fig. 5c) was hereby made based on state-of the art knowledge.

**Fig. 6:**
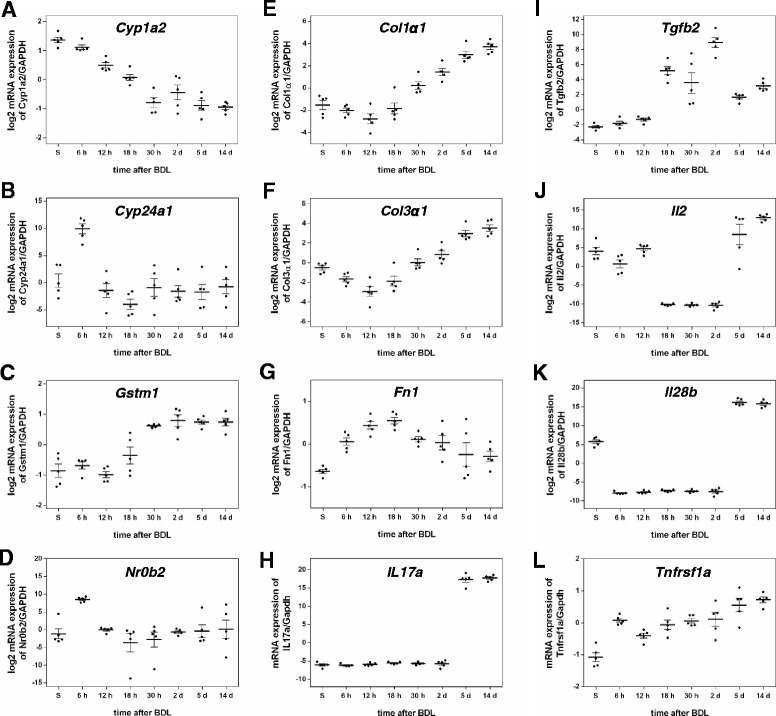
mRNA quantification of selected genes by Fluidigm real-time PCR displayed in log2 scale. **a** Cyp1a2, (**b**) Cyp24a1, (**c**) Gstm1, (**d**) Nr0b2, (**e**) Col1α1, (**f**) Col3α1, (**g**), Fn1, (**h**) Il17a, (**i**) Tgfb2, (**j**) Il2, (**k**) Il28b, (**l**) Tnfrsf1a. Values are given in means ± SEM of five independent experiments per time point

**Fig. 7:**
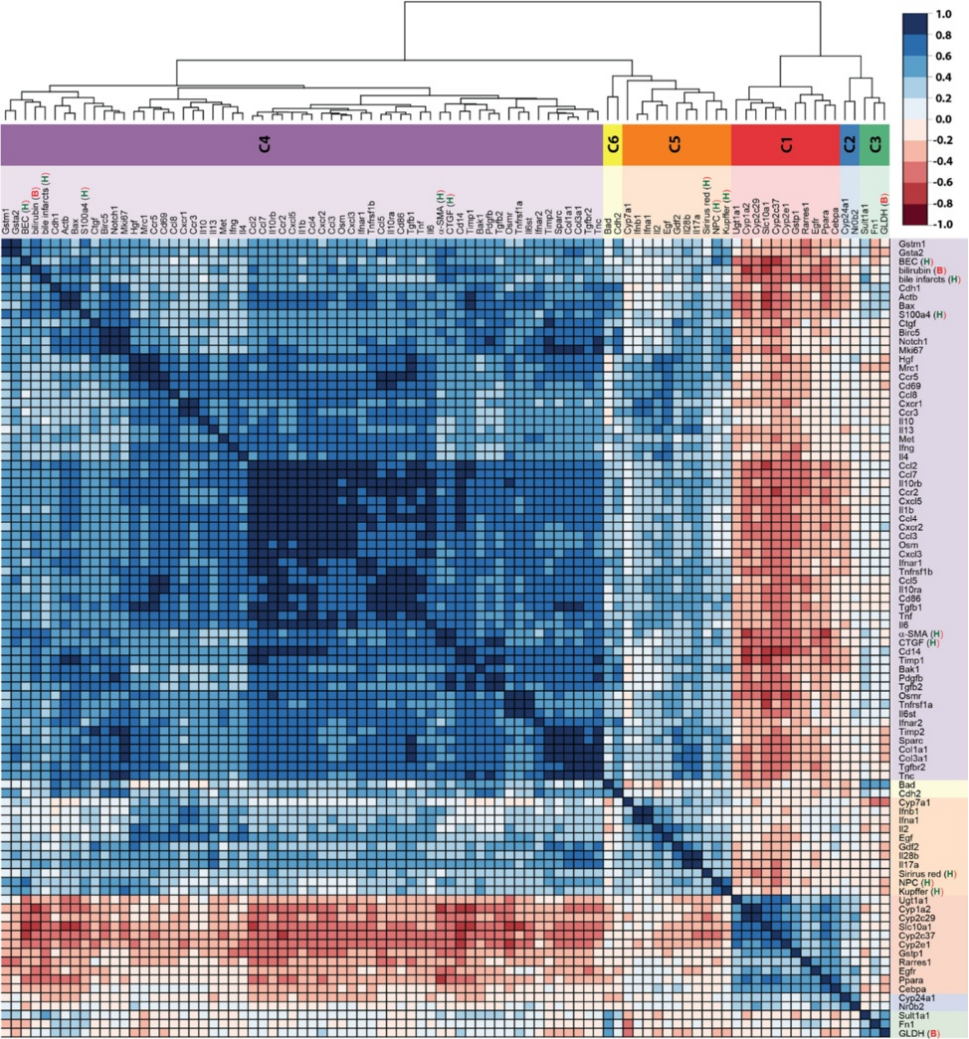
Correlation matrix of factors. Matrix of correlation coefficients between subset of factors, which changed significantly after BDL as determined by ANOVA. Correlation coefficients are YS3 correlations, with positive correlation depicted in blue, negative correlation in red, according to color key. Side dendrogram shows the results of hierarchical clustering with the resulting six time course clusters c1-c6 marked in the color sidebar (see Fig. 9 for time courses corresponding to the individual clusters). Histological factors are marked with H, immunostainings with A, and biochemical factors with B. The list of full names corresponding to the factor abbreviations is provided in Additional file 2, gene probes

**Fig. 8:**
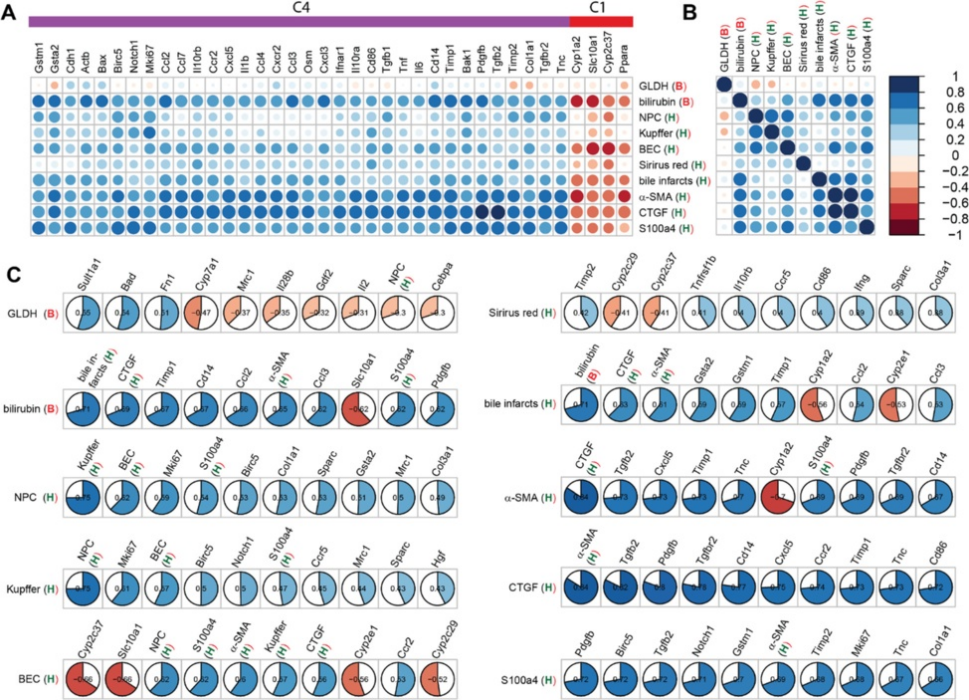
Histological (H), biochemical (B), and immunostaining (A) correlations. Top correlations between classical and transcriptional factors (numerical values provided in Additional file 2). Correlation coefficients are YS3 correlations with positive correlation depicted in blue, negative correlation in red, according to color key. **a** Top correlation between histological, biochemical and immunostaining factors with gene transcripts (area of circles corresponds to the correlation coefficients). Only genes with at least one YS3 correlation of abs(YS3) > =0.6 are shown. Genes are sorted based on hierarchical clustering in Fig. 7 with corresponding clusters depicted in the side color bar (C4 and C1). **b** Correlation between histological, biochemical, and immunostaining factors with color coding analogue to **a**. **c** Highest absolute correlations between classical factors (histological, biochemical, and immunostaining), and all other factors. Data sorted from left to right by absolute value of correlation. Color and size of the filled pie corresponds to the respective correlation value, with positive correlation in blue and negative correlation in red

**Fig. 9:**
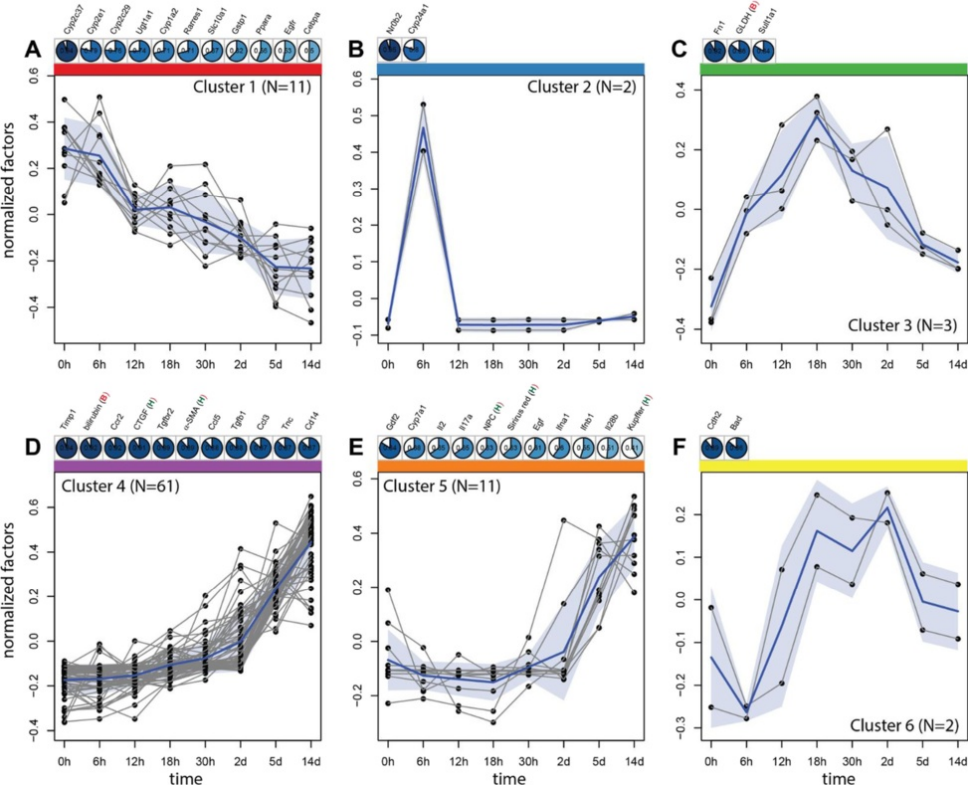
Time course clusters in BDL. Six time course clusters (**a**-**f** corresponding to cluster 1-6) resulting from hierarchical clustering (see Fig. 7). The mean cluster time course (averaged over all factors and repeats) is depicted in blue, all representatives of the respective cluster in grey. The shaded blue area corresponds to the standard deviation between the mean time courses of the representatives in the cluster. The top correlations between the mean cluster time course and factors in the cluster are listed above the time course (color coding analog to Fig. 8c with positive correlations in blue and negative correlations in red) with histological factors marked with H, immunostainings with A, and biochemical factors with B. The cluster members are fully enumerated for all clusters with exception of cluster 4. The full set of members and respective correlation to the mean cluster time course for cluster 4 are: Timp1 (0.94), bilirubin (B 0.92), Ccr2 (0.92), CTGF (A 0.91), Tgfbr2 (0.89), α-SMA (A 0.89), Ccl5 (0.88), Tgfb1 (0.88), Ccl3 (0.87), Tnc (0.87), Cd14 (0.87), Ccl2 (0.86), Cd86 (0.86), Pdgfb (0.86), Col1a1 (0.86), Cxcl3 (0.86), Ccl4 (0.85), Cxcl5 (0.85), Il10ra (0.85), Col3a1 (0.85), Il10rb (0.84), Ccl7 (0.82), Cd69 (0.82), Ifnar1 (0.82), Tnf (0.82), Osm (0.81), Sparc (0.8), Il6 (0.8), Tnfrsf1b (0.8), Cxcr2 (0.78), Il1b (0.78), Timp2 (0.77), Ifnar2 (0.77), Ccr5 (0.77), Il10 (0.76), Osmr (0.75), Gsta2 (0.74), Il4 (0.71), Ifng (0.71), Ccl8 (0.71), Hgf (0.7), Bak1 (0.7), Mrc1 (0.69), Tgfb2 (0.69), Ccr3 (0.68), Actb (0.68), S100a4 (A 0.66), Il13 (0.66), Met (0.66), bile infarcts (H 0.65), Il6st (0.63), Tnfrsf1a (0.63), Mki67 (0.62), Birc5 (0.6), Ctgf (0.58), BEC (H 0.56), Bax (0.56), Notch1 (0.54), Cxcr1 (0.51), Gstm1 (0.45), Cdh1 (0.42)

In a first step, ANOVA was applied to reduce the complete set of biochemical, histochemical and transcript data, consisting of 153 parameters (=factors), to a subset showing significant (p_ad j_ < 0.05) changes during the disease time course (Additional file 2, dimension reduction). This reduced the number to 90 factors, comprising two biochemical markers (bilirubin, GLDH), eight (immuno)-histological markers (BEC, NPC (non-parenchymal cells), Kupffer cells, Sirius red, bile infarcts, CTGF, α-SMA, S100a4) and 80 genes (14/47 ADME-, 22/46 fibrosis-, 44/47 inflammation-panel). Many of the ADME- and fibrosis-genes were filtered out, whereas almost all genes of the inflammation panel were retained. The top significant factors were: Cyp1a2 (Fig. 6a), serum bilirubin (Fig. 1c), Il10rb, Tgfb1, Ccl2, Cd86, Ccr2, and Mrc1. Within the filtered subset, a bivariate time-dependent correlation analysis (Methods and Additional file 2 correlation analysis) was performed for all pairs of factors to identify those displaying similar temporal profiles (Fig. 7), with the top correlations for biochemical, histological and immunostaining factors depicted in Fig. 8. Based on the obtained correlation matrix, a hierarchical cluster analysis was applied, resulting in 6 clusters with distinct time courses that comprise between 2–61 factors, and which, attain their maximum at different time points (see Fig. 9). The identified clusters comprise both ‘classical’ biochemical and histochemical factors as well as genes characteristic for a specific phase of disease progression.

### Correlations between transcripts and non-transcript factors

The time course of each ‘classical’ factor contained in the ANOVA-subset can be correlated with the expression time course of at least one gene (Fig. 8a). Only for GLDH and Sirius Red, the correlations are weak. Notably, all top correlations to genes come either from cluster 4 or cluster 1. Bilirubin, bile infarcts and immunostainings (α-SMA, CTGF and S100a4), all have high correlations among each other, so do the BrdU positive BEC, KC and HSC measurements (Fig. 8b). GLDH and Sirius red do not show high correlation with any other classical factor. In the following, the top correlated factors are discussed in the context of different aspects of the disease process (Fig. 8c). 

#### Initial response

Immediately after BDL, there is a massive release of liver enzymes until day 5, followed by a drop down to almost values of sham-operated livers (Fig. 1a, b). GLDH and ALT show a strong initial increase, whereby GLDH in contrast to ALT increases further up to 18 h, before its gradual decrease. GLDH is highly correlated with members of cluster 3 (Figs. 8c and 9c), the early up-regulated transcripts Fn1 (fibronectin, Fig. 6g) and Sult1a1 (Sulfotransferase 1A1).

Initial molecular events with strong transcript peaks are visible at 6 h for members of cluster 2, the transcriptional regulator Nr0b2 (small heterodimer partner, SHP, Fig. 6d) and Cyp24a1 (mitochondrial 1,25-dihydroxyvitamin D3 24-hydroxylase, see Fig. 6b). Nr0b2 was previously associated with cirrhosis and hepatic tumors [23]. A functional role for Shp was supported by the fact that cholestatic liver fibrosis induced by BDL is increased in SHP−/− mice [24]. Additionally up-regulated transcripts at 6 h (Additional file 2, *t*-test for initial phase) are Tnfrsf1a (Fig. 6l), Il6st (Interleukin-6 receptor subunit beta), Osmr, Cd14, Cxcl1/2, Timp1 and Hmox1 (heme oxygenase), the latter in line with reported marked increase in heme oxygenase activity following BDL in rats [25]. Marked initial down-regulation is present, among others, for Cdh2, Pde4a and the main enzyme of bile acid synthesis Cyp7a1 (cholesterol-7-α-hydroxylase), which can be interpreted as a fast and straightforward response to cholestasis. As underlying mechanism for such expression down-regulation, activation of the JNK/c-Jun pathway has been proposed [26].

#### Macroscopic organ damage

Necroinflammation is caused by BDL-induced intrahepatic toxic bile accumulation with individual liver cell death and progressive development of confluent bile infarct areas, as documented by H&E staining in Fig. 2b. The total area of infarcts increases steadily with relatively high variance (Fig. 2a). Bilirubin (Fig. 1c) shows the highest correlation with bile infarcts, followed by the immunostainings for CTGF (Fig. 3f) and α-SMA (Fig. 3b). At the mRNA expression level, Gsta2 (Glutathione S-transferase A2), Gstm1 (glutathione-S-transferase mu 1, Fig. 6c) and Timp1 (Metalloproteinase inhibitor 1) display the highest positive correlation. Timp1 is a metalloproteinase inhibitor that functions by forming one to one complexes with target metalloproteinases, such as collagenases. In contrast, Cyp1a2 (Cytochrome P450 1A2, Fig. 6a) and Cyp2e1 (Cytochrome P450 2E1) are highly anti-correlated to the bile infarct area, with Cyp1a2 decreasing continuously after BDL, which is in line with observations in rats [27].

#### Loss of liver function

Liver function after BDL was representatively measured using albumin (Fig. 1d) and bilirubin (Fig. 1c) levels. Surprisingly, albumin synthesis is maintained relatively constant over the observation period of 14 days, and was consequently filtered out via ANOVA. Bilirubin, on the other hand increases continuously after BDL. The highest positive correlation with serum bilirubin levels is observed for the transcripts Timp1, Cd14, Ccl2 (chemokine C-C motif ligand 2), a soluble biomarker for hepatic fibrosis in NAFLD [28], and Ccl3 (Fig. 8c). Notably, a very high negative correlation is present for bilirubin and Slc10a1 (Sodium/bile acid cotransporter), encoding the Na + −taurocholate co-transporting polypeptide, which transports bile acids as part of the hepatic sodium bile acid uptake system. The decrease in Slc10a1 has been shown to protect hepatocytes from cholestasis-induced injury [29].

#### Hepatic cell proliferative response

During disease progression, various hepatic cell types start proliferating, as documented by (co)-immunostaining with BrdU and cell type specific markers (Fig. 3), and which is indirectly reflected by the marked up-regulation of Ki67 mRNA (Fig. 4a). The observed time course is principally very similar in hepatocytes, KC and BECs, resulting in a high correlation within this group (Fig. 8b).

Hepatocyte proliferation occurs between 30 h and 2 days, as monitored by the parameter BrdU-positive hepatocytes (Fig. 3d). S100a4 positive cells represent Kupffer cells (KC, liver macrophages), which infiltrate the damaged liver tissue, become activated and proliferate starting at 30 h, to reach a maximum at day 2 and to decrease proliferative activity again thereafter. KC numbers are highly correlated to the transcript Mki67 (antigen Ki-67), a known proliferation marker, followed by the transcripts Birc5 (Baculoviral IAP repeat-containing protein 5, survivin) and Notch1, a transmembrane receptor involved in developmental processes (Fig. 8c).

BEC display the highest proliferative activity between day 2 and 5 after BDL, with minor activity after 30 h. Interestingly, the highest correlations between BECs and transcripts are all negative, namely Cyp2c37 (Cytochrome P450 2C37), Slc10a1, Cyp2e1, and Cyp2c29 (Fig. 8c). From these, Cyp2c37 and Slc10a1 are interesting candidates, since they are with Cyp1a2 and Ppara the only factors from the top correlations (Fig. 8a) which are from time course cluster C1 and have high negative correlations with the classical factors.

#### Increase in fibrogenic cells

In accordance with the reported proliferative activity of HSCs and recruitment of KCs to the area of injury, immunohistochemical analyses demonstrate the gradual rise in CTGF- (Fig. 3f), α-SMA- (Fig. 3b) and S100a4-expressing cells (Fig. 3c). These markers reflect activated HSCs and activated KCs. CTGF and α-SMA are highly correlated to each other (respective top correlation Fig. 8c), with S100a4 having a strong correlation to both of them (Fig. 8b).

CTGF is a highly pro-fibrogenic protein expressed by HSCs, BECs and hepatocytes [30, 31] and mediates extracellular matrix modulating properties. Levels of CTGF have been reported significantly up-regulated in experimental liver fibrogenesis and human chronic liver disease patients of various etiologies [32, 33]. CTGF-positive cell number is the best candidate to monitor the disease progress among the selected biochemical, histological and immunostaining parameters, showing a steady increase with comparatively little variance (among top ANOVA results, p_adj_ = 7.9E-10). This is consistent with data from other studies, which observed a correlation of increased CTGF levels with histological fibrosis stages [34, 35]. Since CTGF can be measured in patients’ blood, it was suggested as valuable diagnostic marker with potential application in the follow-up of patients suffering from chronic liver diseases [36]. The highest transcript correlation with CTGF positive cell number shows Tgfb2 (cytokine TGF-β2, Fig. 6i), followed by Pdgfb (platelet-derived growth factor subunit B). TGF-β is the major stimulus for CTGF expression in hepatocytes [37], and elevated levels of Tgfb2 were reported for BDL rats [38]. Pdgfb has been reported up-regulated in liver inflammation and fibrosis [39]. Additionally, there are considerable correlations to several other genes such as Tgfbr2, encoding the transforming growth factor β receptor 2 [40], Cd14, Cxcl5, Ccr2, and Timp1.

α-SMA-positive cells, representing activated HSC, increase steadily during disease progression (Fig. 3b), and are highly correlated to CTGF. Consequently, the top transcript correlations are very similar: Tgfb2, Cxcl4, Timp1, Tnc, and Pdgfb. Notably, α-SMA staining, but also CTGF and S100a, show strong negative correlations to Cyp1a2 (Fig. 6a), known as downregulated in liver cirrhosis [41], and to Ppara (Fig. 8a).

S100a4-positive cells, which are steadily rising until day 2, after which they stay elevated (Fig. 3c), are also good markers for disease progression, presenting with a similar time course than CTGF (Fig. 3b), but with a larger variation from the 18 h time point on. Many transcripts are highly correlated with S100a4, e.g. Pdgfb, Birc5, Tgfb2 and Notch1.

#### Fibrosis

The progression of fibrogenesis is histomorphologically characterized by excessive deposition of extracellular matrix, visible by Sirius red staining of liver slices (Fig. 4b, c). Surprisingly, Sirius red did not display high correlations to other factors (Fig. 8, a-c), mainly due to the high variability in measurements from 0 h to 2 days, and therefore was in this study not a very reliable predictor for fibrogenesis. After day 2, a strong increase in Sirius red was observed. Both Col1a1 (fibrillar collagen 1α1, Fig. 6e) and Col3a1 (fibrillar collagen 3α1, Fig. 6f) transcripts, which predominantly exist in fibrotic livers, show up-regulation beginning 30 h after BDL that continuously increases with severity of liver fibrosis up to 14 days. Among the peptide mediators, Tgfb1 and Tgfb2 (Tgf-β isoforms 1 and 2) expression is increasing after 2 days, confirming their postulated role as fibrogenic master cytokines [42]. Tgfb, encoding the cytokine TGF-β is well known to correspond with the fibrotic process in a positive feedback loop [43]. Further, its expression is associated with induction of fibrogenesis-related genes (Fig. 5b), which particularly are representative for HSC activation. The dynamics of the inflammation gene signature (Fig. 5c) nicely matches with the increase in the number of proliferating Kupffer cells (F4-80/BrdU staining values) observed from day 2 onwards (Fig. 3e). Very low expression levels are present immediately after BDL, except for the chemokines Cxcl1 und 2. Starting at time points between 2 and 5 days after BDL, most cytokines and chemokines in the list were strongly upregulated until day 14. During the perpetuation phase (18 h - 2 days), paracrine and autocrine cytokines amplify hepatic inflammation and HSC activation, resulting in continued ECM remodeling, characterized by enhanced mRNA expression of both, fibrillar collagen1α1 and 3α1 (Fig. 6e and f).

### Markers of disease progression

Main focus of this study was to detect factors and factor combinations which best characterize particular stages of the disease process. Here, we discuss in more detail the biological significance of the six time course clusters.

Cluster 1 (Fig. 9a) decreases continuously over time with no classical factor included in c1. Most of the members (8/11) are from the ADME panel, with exception of Rarres1 and Egfr coming from the fibrosis panel. All members of cluster 1 have very high significance in the ANOVA, but for Rarres1 (*p*_*adj*_ = 0.036). The ADME genes in c1 display decreased expression during late initial, perpetuation and progression phase. Top correlations with the cluster mean time course are in decreasing order Cyp2c37, Cyp2e1, Cyp2e29, Ugt1a1, Cyp1a2 (Fig. 6a), Rarres1 and Slc10a1, remarkably containing many enzymes of the cytochrome P450 system. Down-regulation of Ugt1a1 (UDP-glucuronosyl-transferase 1A), the main enzyme for conjugation of bilirubin, and Slc10a1, encoding the Na + −taurocholate co-transporting polypeptide, which transports bile acids, are protective against the increased concentration of conjugated bilirubin in hepatocytes after BDL.

Cluster 2 (Fig. 9b) consists of strong transcript peaks at 6 h for the transcriptional regulator Nr0b2 (SHP, Fig. 6d) and Cyp24a1 (Fig. 6b), both probes of the ADME chip. Nr0b2 (*p*_*adj*_ = 1.30*E*^− 7^) and Cyp24a1 (*p*_*adj*_ = 9.88*E*^− 3^) both present with very high ANOVA significance. Other transcripts also show an increase in the initial phase at 6 h, for instance members of cluster 3, but none of them decreases to baseline during disease progression from 18 h up to 14 days. This makes Nr0b2 and Cyp24a1 the most interesting candidates for detecting the initial phase of cholestasis (3 h – 6 h).

Cluster 3 (Fig. 9c) is characterized by an increase in the initial phase up to 18 h with subsequent decrease during disease progression up to 14 days. The cluster consists of the biochemical factor GLDH, the fibrosis transcript Fn1 (Fibronectin, Fig. 6g), and the ADME gene Sulf1a1 (Sulfotransferase 1A1), making this cluster interesting because of the combination of various factor types.

Cluster 4 (Fig. 9d) shows continuous increase starting in the initial phase, lasting throughout disease progression up to 14 days. Consequently, members of the cluster are good candidates to predict continuous disease progression (fibrosis). Among the top candidates are bilirubin, CTGF and α-SMA. Cluster 4 is the largest cluster, containing 61/90 significant factors of the ANOVA. Notably, most of the classical markers are contained in cluster 4, that is bilirubin, CTGF α-SMA, S100a4, bile infarcts and BEC. All transcripts in c4 either belong to inflammation or fibrosis panels, except Gsta2 and Gstm1 (ADME panel). The top transcript correlation with the cluster mean comprises Timp1, followed by Ccr2, and Tgfbr2, with a large number of transcripts showing high correlation to the cluster mean.

The time course of cluster 5 (Fig. 9e) is highly similar to c4, but increasing only after around 30 h compared to the continuous increase of c4 starting already in the initial damage phase. Cluster 5 contains the classical markers NPCs, Kupffer cells and Sirius red. Top correlating transcripts are Gdl2 and Cyp7a1, which show increasing values starting at around 30 h, despite a strong down-regulation in the initial phase (see above). Notably, the interleukins Il2, Il17a (interleukin-17A, Fig. 6h) and Il28b (interleukin 28β, Fig. 6k), altogether secreted proteins, are members of c5, and are, likely detectable in blood, thus representing candidate diagnostic markers. Il17a (interleukin-17A, Fig. 6h), plays a pivotal role in cholestatic liver fibrosis in mice by participation in activation of KCs and HSCs [44].

Cluster 6 (Fig. 9f) is characterized by an initial decrease, followed by an increase in the late initial phase at 12 h up to 2 days, and subsequent decrease during disease progression at 5 days and 14 days. Both cluster members, Cdh2 (cadherin 2) and Bad1, are part of the fibrosis panel. Cluster 6 shows a similar up and down regulation than c3, but the increase starts later, the decrease starts later and the maximum transcript values are consequently shifted to a later time point.

The large majority of histologic parameters, cell count observations, as well as most genes related to inflammation and fibrogenesis increased with disease progression, either in a continuous manner starting in the perpetuation phase after around 1 day or latest beginning at day 5 (cluster 4 and 5). Based on the strong increase between day 2 and 5 in cluster 5 (but also in cluster 4), we conclude that transition from disease stage at day 2 to progression at day 5 can be easily monitored. This transition could provide valuable information for clinical practice, as serum bilirubin is among the top correlation candidates of cluster 4, the interleukins (Il2, Il17a, Il28b, Fig. 6h and k) of cluster 5, as well as the growth factors (Pdgfb, Tgfb1, Tgfb2, Hgf; Fig. 6i) of cluster 4, all of which are secreted factors and can be measured in blood. Furthermore, additional prominent candidates of cluster 4 are extracellular matrix components (Sparc, Col3a1, Col1a1, Fig. 6f and e).

Cluster 4 is representative for disease progression due to the continuous increase of factors starting already in the initial phase. Counting CTGF-positive cells presents as the most reliable overall measure for disease progression at histological level, bilirubin at biochemical level, and metalloproteinase inhibitor 1 (Timp1) at transcript level.

Interestingly, no histological, biochemical or immunostaining based factors were found in clusters c1, c2 and c6. The transcripts in these clusters provide unique time course information, which cannot be captured with the subset of analyzed histological markers, thereby providing crucial information for the initial and perpetuation phase, not attainable via c3, c4 and c5.

### Decision trees for disease progression

**Fig. 10:**
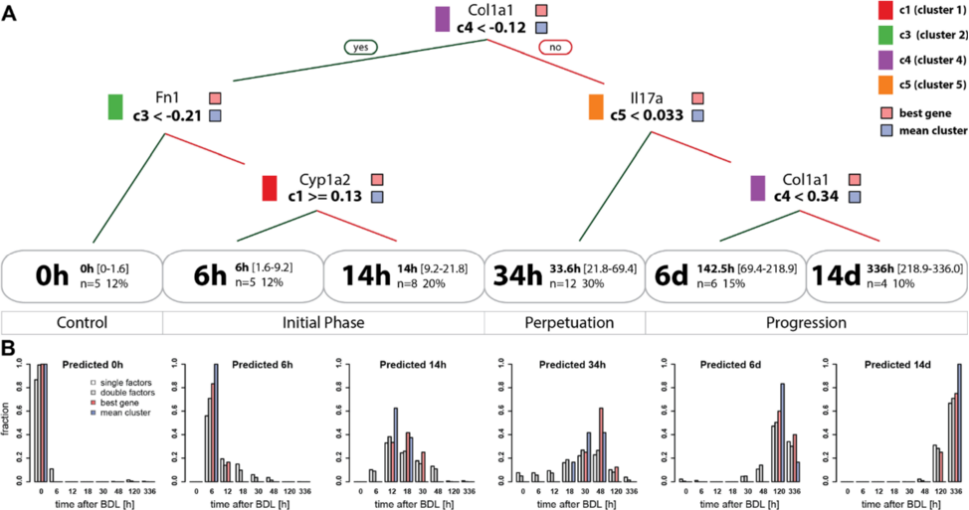
Decision tree for disease progression. (**a**) Regression tree for the prediction of time phases after BDL based on mean cluster time courses (Fig. 9). Splitting rules are depicted at the respective branching points of the tree, with left branches correspond to ‘yes’, right branches to ‘no’ decisions. In addition to the cluster used in the decisions, also the best gene representatives from the respective cluster is shown above the decision rule. The regression tree classifies the data in six time classes 0 h, 6 h, 14 h, 24 h, 6d, 14d with information on mean time, range, number and percentage of samples falling in the respective class listed. In addition to the tree based on the mean cluster time courses (mean cluster), the best tree only using a single gene transcript from every cluster is shown (best gene). The best decision tree based on genes, histological, biochemical, and immunostaining factors (not shown) is highly similar to the depicted best gene tree, with the single change of using S100a4 instead of Col1a1 for the decision on cluster c4 and allowing GLDH as equally good alternative to Fn1 in c3. (**b**) Predictive performance of decision tree. The predictive performance of the regression tree was evaluated using all single factor combinations from the individual clusters (white), a random sample (N = 10000) of two factors from each cluster (gray), the best gene representative tree (red), and the mean cluster data (blue, trainings data). For all classes of the decision tree the histogram of predicted vs. experimental data are shown

We next wanted to determine, which of the analyzed factors are best suited as markers for particular stages of the disease process. To this end, we constructed a decision tree (Fig. 10) based on the time course of the normalized clusters (Fig. 9). This so called regression tree allows to predict a specific time interval of the disease process upon combining the dynamics of factors from the clusters (Fig. 10). The algorithm used for constructing the decision tree avoids over-fitting of data by balancing the number of knots against robustness of the classification tested by cross-validation. Consequently, the decision tree assigns a cluster pattern to time intervals of disease progression, rather than to discrete experimental time points, which results in 6 time classes. Interestingly, mainly time points in late initial and perpetuation phases (12 h, 18 h, 30 h, 48 h) were merged into time classes, whereas the control (0 h), early initial (6 h), and progression phases (5 days, 14 days) remained almost unchanged. Robustness of predicted time classes and prediction performance were tested with a leave one out approach, resulting in reproducible time classes and good prediction performance, when using left out test data (Additional file 2, decision tree).

The resulting regression tree exploits time course information from clusters c1, c3, c4 and c5, whereas clusters c2 and c6 are not used. Cluster 4 possesses the strongest discriminating power, performing the important split between early phase after BDL (classes 0 h, 6 h and 14 h with range 0 h-21.8 h) and later perpetuation and progression phases (classes 34 h, 6 days,14 days, with range 21.8 h-14 days). If the mean value of a factor in cluster c4 is smaller than −0.12 at a measured time point, this time point is classified as not later than 21.8 h. A more detailed assignment of the respective time interval requires interrogation of additional clusters. The value of c3 decides between control and initial phase, with subsequent splitting based on c1 into early and late initial phase. Analogue, the value of c5 decides between perpetuation and progression phase, with subsequent c4 based splitting in early and late progression phase. Note that the values of cluster 4 appear twice in the decision tree, owing to the monotonous increase after BDL.

The predictive performance of the regression tree for the mean cluster data is depicted in Fig. 10b (blue), providing information of experimental time point classifications. All samples of control, 6 h, and 14 days are assigned to their respective time classes 0 h, 6 h and 14 days, whereas neighboring time points are combined in classes 14 h, 34 h and 6 days. In addition, we evaluated the decision tree based on a subset of factors from time course clusters, using either a single or two factors randomly chosen from each cluster, and used their values for predictions (Fig. 10b, single and double factors). Even with only one factor selected from c1, c3, c4 and c5, surprisingly consistent classifications were achieved. As expected, the quality of predictions improved by increasing the number of factors. With our approach, we are able to predict control, early initial and late progression phases with high accuracy, whereas the intermediate phase of disease progression shows more misclassifications, in case of single and double factor analyses.

The best performing decision tree based on a single transcript from each cluster (Fig. 10a) splits on Col1a1 (Collagen alpha-1(I) chain, c4), Fn1 (Fibronectin, c3), Cyp1a2 (Cytochrome P450 1A2, c1), and Il17a (Interleukin-17A, c5), all important factors involved in disease progression (discussed above). The best performing decision tree based on all factors, i.e. histological, biochemical, immunostaining factors, and transcripts, resulted in a highly similar tree to the best tree based solely on transcription factors, with the single change of using S100a4 instead of Col1a1 for c4 splitting, and providing GLDH as alternative factor to Fn1 to perform the c3 split. By selecting a subset of good performing transcripts, a very good prediction performance can be achieved already on a small subset of factors (Fig. 10b red).

### Individual variability

We observed a large variability for many analyzed factors, when comparing individual mice of the same time points. For example at day 5, the infarct area varies between 0.9 % and 12 % (Fig. 2a), and the collagen deposition area measured by Sirius red varies between 0.8 % and 5.9 % (Fig. 4b). Similar inter individual variability can be observed for proliferative activity (BECs Fig. 3a, Kupffer cells Fig. 3e) or expression of collagen (Col1a1 Fig. 6e, Col3a1 Fig. 6f), to name a few. Such large variation in parameters during perpetuation and especially progression phases are an intriguing finding, considering homogeneity of the experimental system and the relatively short time frame of 14 days (see also heatmap of time courses in Additional file 2). A possible explanation is different individual pace of disease onset or progression due to variations in susceptibility and/or repair activity to the damage induced by BDL. As a consequence, heterogeneous time courses develop, with highly affected mice showing strong signs of fibrosis earlier.

Another hypothesis for this result is variable routes of disease progression. For example, one route is characterized by a strong increase in necrotic tissue and a weaker activation of HSCs with lower expression changes of inflammation factors. Another route is represented by strong activation of fibrogenesis factors, and finally macroscopically visible scar tissue. Both routes are similarly connected to loss of liver function, however, histopathological presentation is quite different. The first route is depicted with a large amount of necrotic tissue, while the second route contains large areas of fibrotic tissue. Such alternative disease routes could be of far-reaching importance for an individualized therapy, as obviously medical interventions avoiding necrosis significantly differ from interventions to reduce overshooting fibrosis. The design of the study, which included sacrification of mice after a defined time, did not allow to answer whether alternate developments, as macroscopically identified in late time points, can be similarly differentiated at earlier time points.

## Conclusion

**Fig. 11:**
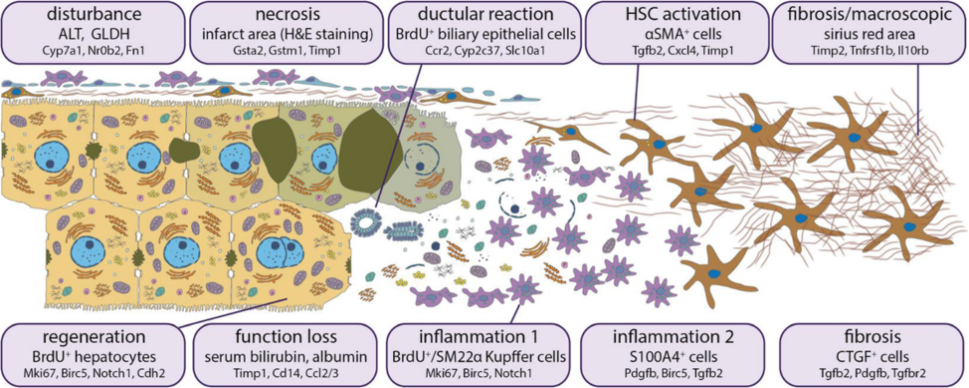
Outline of the disease process. Each box is dedicated to a specific disease aspect (first line), which is represented by a commonly known marker (second line) or by several markers. Below (in small font), genes are given, whose expression is correlated to the factor above

The time resolved analysis of a wide range of parameters in bile duct ligated mice has shown that many of the preselected factors share the pattern of increase throughout disease progression (Fig. 9). Particularly pronounced changes were observed during transition from perpetuation to progression phase, 2 to 5 days after BDL, characterized by strong increases of parameters, such as Il17A, Il2, Il28b or Il13. This information has strong clinical relevance, as it indicates a robust switching point, and human homologs of the respective interleukins are top candidates to be used as clinical markers. Main points are summarized in Fig. 11, bringing the different aspects, phases and markers together.

Our experiments also found previously unknown molecular events, which are probably elements of a wider transcriptional reprograming related to damage or tissue repair activity. For instance, there are strong transcript peaks for SHP (Nr0b2) at 6 h, which now needs a focused analysis to delineate the chain of molecular interactions causing it and the functional consequences for disease onset and progression or its repair.

Based on time course correlation analyses, we found a distinct molecular and pathomorphological patterns related to disease phases following BDL. From a subset of parameters of these patterns, we propose a decision tree, such as in Fig. 11, as a promising tool for assessment of disease progression. Notably, our approach allows prediction of disease progression from an arbitrary subset of measured parameters. Therefore, as a next step, suitability of the parameters selected from the mouse model need to be confirmed for human patients as e.g. in [45]. In this study, the relationship of portal inflammation to ductular reaction and thus, the correlation with disease severity was investigated in NAFLD liver biopsy sections by analyzing selected inflammatory and broad leukocyte subset markers [45].

Translation from mouse models into human patients is challenging, however, should be the final aim of all studies with animal disease models. One major drawback is that animal disease models develop in a very short time frame, e.g. 14 days for BDL, whereas in human, a chronic liver disease in many cases requires decades until occurrence of a progressed disease stage. Another challenge is that except from blood samples, no time course estimation of disease dynamics can be performed in human, since usually only one or in best cases a second biopsy sample is available for a patient. However, we believe that a thorough analysis of multiple parameters in such patient samples can be matched with dense time course analyses of animal models as presented in the present report and, upon further optimization, finally may lead to improved estimation of the patients disease stage and therapy decisions.

Many of the measured parameters display quite large variability, which may be one of the reasons why translation of a set of such parameters into diagnostic approaches has not proven sufficiently robust for predictions in human patients with chronic liver diseases. The here suggested approach of pooling information of factors falling in the same time course classes could be a possible solution for more robust predictors in the future.

Taken together, the detailed time-resolved profiling of mouse liver samples following BDL revealed a coordinated response, resulting in disease phase dependent modulations at morphological, biochemical, metabolic and gene expression levels, which can be used as diagnostic markers to predict a disease stage more thoroughly. Such approach is recommended for human patient cohorts, to generate similar prediction trees based on estimating a maximum amount of parameters for improved diagnosis.

To further elucidate the regulatory network behind the disease stage related expression signatures, additional studies are needed, which have to include knowledge on transcription factor activation due to accumulation of bile salts and shared transcription factor binding motifs of genes belonging to the same transcript cluster.

### Availability of supporting data

The data sets supporting the results of this article are included within the article and its additional files.

## Additional files

Additional file 1:
**Dataset S1.** Measurements in bile duct ligated mice. (PDF 191 kb) Additional file 2:
**Dataset S2.** Statistical analysis. (PDF 943 kb)

## Abbreviations

CTGF: Connective tissue growth factor
BDL: Bile duct ligation
SHP: Small heterodimer partner
CLD: Chronic liver disease
HSC: Hepatic stellate cells
KC: Kupffer cells
ADME: Absorption, distribution, metabolism, and excretion
ALT: Alanine aminotransferase
GLDH: Glutamate dehydrogenase
EDTA: Ethylenediaminetetraacetic acid
H&E: Hematoxylin and eosin
