# Supplementary material for: Pathobiochemical signatures of cholestatic liver disease in bile duct ligated mice
Source: BMC Syst Biol. 2015 Nov 20;9:83. doi: 10.1186/s12918-015-0229-0 (PMC4654904; doi:10.1186/s12918-015-0229-0)
Supplement: Additional file 1: — Dataset S1. Measurements in bile duct ligated mice. (PDF 191 kb) [file 12918_2015_229_MOESM1_ESM.pdf]

Measurements in bile duct ligated mice

Andreas Hoppe, Kerstin Abshagen, Maria Thomas

September 16, 2014

Contents

1 **Main factors** 2

2 **RNA ADME genes** 3

3 **RNA Fibrosis genes** 5

4 **RNA Zytokine genes** 7

ALT,GLDH	U/l
Bilirubin	mmol/l
Albumin	mg/ml
area	percentage number, share
$BrdU^+$ cells	n/mm^2, cell count
SMA,CTGF,S100A4	n/20x, cell count
RNA	ratio to GAPDH expression

Table 1 – units

1 Main factors

ID	ALT	Albumin	Bilirubin	GLDH	BrdU hepatocytes	BrdU non-parenchym	BrdU Kupffer	BrdU BEC	Collagen area	Infarct area	SMA cells	CTGF cells	S100A4 cells
IM 1 0h 17.12.09	26.5	21.2	1.49	16.3	0.08	0.28	0.09	0	1.38%	0%	n.m.	n.m.	n.m.
IM 2 0h 17.12.09	27.6	26.1	0.93	9.3	0.04	0.1	0.13	0	1.18%	0%	n.m.	n.m.	n.m.
IM 3 0h 06.01.10	28.6	23.8	1.65	8.4	0.02	0.38	0.16	0.01	1.21%	0%	2	1	12
IM 4 0h 06.01.10	28.6	17.8	0.85	10.4	0.03	1.33	0.11	0	1.88%	0%	1	2	16
IM 5 0h 06.01.10	33.2	19.2	0.53	20.6	0.13	0.4	0.13	0.03	2.65%	0%	2	1	13
IM 2 6h 11.12.09	410	40.6	25.8	516	0.44	0.44	0.19	0	1.36%	0.19%	3	2	13
IM 3 6h 11.12.09	661.8	30.8	13.9	734.4	0.03	0.22	0.11	0.03	0.39%	0.15%	n.m.	n.m.	n.m.
IM 4 6h 11.12.09	772.9	19.1	10.7	1102	0.09	0.33	0.11	0.02	0.43%	0%	6	1	17
IM 3 6h 01.12.09	570.8	23.1	15.1	2220	0.03	0.36	0.11	0	0.45%	0%	5	3	13
IM 4 6h 01.12.09	249.8	20.1	8.96	652	0.09	0.52	0.23	0.02	0.12%	0%	n.m.	n.m.	n.m.
IM 1 12h 08.12.09	678	24.6	31.5	1492	0.03	0.15	0.07	0	0.25%	0.92%	15	9	15
IM 2 12h 08.12.09	678	22.4	22.5	1340	0.02	0.25	0.1	0.1	1.78%	0.46%	14	10	19
IM 1 12h 11.12.09	799	27.9	39	1206	0.05	0.23	0.1	0.13	1.32%	2.13%	12	6	18
IM 1 12h 14.12.09	483	24	25.8	869	0.05	0.21	0.14	0.01	2.25%	0.02%	n.m.	n.m.	n.m.
IM 2 12h 14.12.09	362	25.5	27.2	722	0.07	0.19	0.13	0.06	1.85%	0%	n.m.	n.m.	n.m.
IM 1 18h 17.12.09	647	29.4	59.4	1520	0	0.07	0.07	0.01	0.76%	0.26%	n.m.	n.m.	n.m.
IM 2 18h 17.12.09	296.5	39.7	34	2836	0.03	0.14	0.09	0.03	1.4%	0.12%	23	17	23
IM 3 18h 17.12.09	604.5	36.9	55.8	1526	0.02	0.14	0.08	0	0.35%	2.18%	34	16	25
IM 4 18h 26.11.09	946	24.5	90.1	3140	0.1	0.2	0.06	0	1.53%	1.75%	17	19	20
IM 5 18h 26.11.09	508.5	24.9	43.3	1546	0.06	0.18	0.09	0	0.66%	3.22%	n.m.	n.m.	n.m.
IM 1 30h 08.01.10	524	22.4	47.1	1048	0.07	1.06	0.93	0.08	3.9%	1.32%	45	19	25
IM 2 30h 08.01.10	346	17	32.3	331	0.17	0.64	0.24	0.04	0.59%	0.28%	69	20	29
IM 3 30h 08.01.10	1146	23.5	84	3794	0	0.05	0.04	0.01	0.85%	2.55%	n.m.	n.m.	n.m.
IM 3 30h 15.12.09	895	20.9	92.7	2134	0.04	0.6	0.31	0.15	1.13%	3.07%	18	14	32
IM 4 30h 15.12.09	243	47.2	84	802	0.02	1.15	0.97	0.09	1.32%	3.17%	n.m.	n.m.	n.m.
IM 1 2d 03.12.09	473.5	23.8	109	602.2	0.97	3.37	n.m.	n.m.	0.95%	4.89%	51	36	54
IM 2 2d 03.12.09	976.9	23.8	144.2	1084	0.23	2.48	1.58	0.75	1.61%	2.01%	76	41	61
IM 3 2d 10.12.09	314.6	25.9	79.1	554.8	1.56	1.78	1.47	0.4	0.65%	1.43%	32	42	40
IM 4 2d 10.12.09	160	46	85.1	670	0.04	0.44	0.12	0.1	0.24%	1.27%	n.m.	n.m.	n.m.
IM 5 2d 10.12.09	236.9	25.6	66	542	0.2	3.05	1.49	0.4	0.75%	0.34%	n.m.	n.m.	n.m.
IM 1 5d 21.04.10	281.1	29.1	241	172.7	0.47	4.13	1.31	1.52	1.75%	1.38%	n.m.	n.m.	n.m.
IM 2 5d 21.04.10	945.2	29.4	249.8	216.9	0.92	3.84	0.7	1.2	0.8%	11.7%	89	66	44
IM 3 5d 21.04.10	587.4	27.7	192.5	390	0.54	3.02	1.25	2.08	1.12%	4.62%	83	53	47
IM 4 5d 07.12.09	n.m.	n.m.	n.m.	n.m.	0.64	4.52	1.09	n.m.	n.m.	n.m.	n.m.	n.m.	n.m.
IM 5 5d 21.04.10	324.9	29.7	373.2	486.6	0.58	3.34	0.93	1.63	5.9%	0.88%	45	41	55
IM 4 5d 21.04.10	675	42.6	279.7	321.9	1.94	3.41	0.57	1.85	1.39%	5.82%	n.m.	n.m.	n.m.
IM 1 14d 09.12.09	157	35	401.5	246	0.06	2.03	0.59	1.35	2.72%	8.4%	112	79	51
IM 2 14d 09.12.09	153	28.4	210	117	0	0.13	0.12	0	6.71%	5.01%	n.m.	n.m.	n.m.
IM 3 14d 09.12.09	93	30.4	368	123	0.13	1.54	0.31	1.62	5.86%	6.4%	123	85	55
IM 1 14d 16.12.09	235	41.6	239.9	108	0.62	4.52	1.32	2.24	4.44%	5.09%	n.m.	n.m.	n.m.
IM 2 14d 16.12.09	199	23.7	319.5	68	0.54	6.53	1.41	2.17	7.21%	1.49%	87	87	55

2 RNA ADME genes

ID	Ppara	Cyp3a11	Cyp24a1	Nos2	Nfkbia	Cyp2c39	Nr3c1	Cyp1a2	Abcb1a	Sult1b1	Gstp1	Cxcl15	Cebpa	Nr1h3	Sod2	Sult1a1	Gstm1	Cyp4a10	Hmox1	Ahr	Cebpd	Slc10a1	Gsta2	Dpyd
IM 1 0h 17.12.09	1.42	0.89	9.76	0.4	0.18	0.25	0.92	2.47	0.16	1.21	1.14	0.004	1.4	0.66	0.7	0.48	0.54	2.5	0.46	0.56	0.49	1.53	0.67	1.14
IM 2 0h 17.12.09	2.03	0.87	0.14	0.36	14.3	0.68	1.47	3.19	0.43	2.54	1.36	191.8	2.47	1.32	1.13	0.63	0.82	2.36	0.43	0.96	1	2.2	0.9	1.49
IM 3 0h 06.01.10	1.8	1.07	9.5	0.47	0.23	0.39	0.93	2.58	0.52	1.46	1.42	0.28	1.91	0.98	1.01	0.56	0.38	1.47	0.53	0.7	0.33	1.74	0.68	1.01
IM 4 0h 06.01.10	2.59	0.6	0.9	2.78	91.9	1.59	1.77	2.67	1.76	3.12	1.2	1465	1.9	1.38	1.34	0.7	0.73	2.16	0.55	1.12	2.03	2.36	1.19	1.4
IM 5 0h 06.01.10	1.56	0.5	0.37	0.09	0.1	0.31	0.9	2.13	0.25	0.95	1.51	0.0005	1.27	0.82	0.86	0.54	0.41	0.16	0.27	0.73	0.77	1.41	0.23	1.13
IM 2 6h 11.12.09	2.02	0.83	2819	10.3	304.2	2.9	1.88	2.06	2.75	3.24	1.26	4897	1.76	1.58	1.28	0.94	0.59	8.02	1.93	1.25	5.92	1.47	0.67	1.18
IM 3 6h 11.12.09	1.41	0.72	3663	12.8	346.2	3.34	1.84	2.02	3.72	3.5	1.06	6443	1.47	1.6	1.5	1.23	0.69	4.5	2.2	1.39	5.35	1.25	1.29	1.3
IM 4 6h 11.12.09	1.23	0.77	1442	3.8	150.2	1.63	1.36	2.07	2.17	2.75	1.09	2231	1.1	1.11	1.22	1.23	0.7	4.08	2.17	0.98	2.76	1.41	0.75	1.2
IM 3 6h 01.12.09	1.17	0.96	125.7	0.26	0.01	0.28	0.73	2.09	0.17	0.92	1.15	0.1	1.12	0.7	0.77	0.69	0.47	4.05	0.91	0.72	0.79	1.21	0.49	0.99
IM 4 6h 01.12.09	1.49	0.94	403.3	0.03	0.64	0.4	0.93	2.67	0.2	1.43	1.36	0.37	1.52	0.82	0.88	0.67	0.7	2.88	0.59	0.73	0.66	1.76	0.61	1.22
IM 1 12h 08.12.09	0.83	1.01	0.24	1.69	0.29	1.02	0.94	1.52	1.39	0.93	0.95	0.23	1.01	0.99	1.29	1.28	0.58	1.52	1.51	0.67	0.78	1.45	0.43	1.01
IM 2 12h 08.12.09	1.19	0.8	0.33	1.51	0.31	0.68	0.89	1.76	0.91	1	1.19	0.17	1.05	0.86	1.02	0.87	0.61	1.47	0.99	0.67	0.69	1.83	0.5	0.92
IM 1 12h 11.12.09	1.15	0.58	0.02	0.49	0.24	0.77	0.82	1.25	1.04	0.81	1.1	0.01	0.78	0.95	1	1.14	0.49	0.86	0.61	0.81	0.75	0.98	0.42	0.78
IM 1 12h 14.12.09	1.55	0.44	0.99	1.35	0.54	0.71	0.9	1.31	1.3	0.8	0.82	0.86	0.97	0.92	0.98	1.05	0.43	3.02	1.35	0.67	0.8	1.39	0.36	0.86
IM 2 12h 14.12.09	0.94	0.59	4.44	0.46	0.56	0.52	0.72	1.28	0.65	0.77	0.92	5.2	0.8	0.86	1.08	0.95	0.43	1.61	0.89	0.74	0.8	0.8	0.35	0.71
IM 1 18h 17.12.09	0.96	1.74	0.24	1.04	0.65	1.77	0.93	1.37	1.77	1.03	1.3	24.4	0.87	0.9	1.15	1.54	1.31	3.16	1.73	0.91	1.05	1.48	1.15	0.96
IM 2 18h 17.12.09	0.74	1.17	0.05	0.39	0.25	1.33	0.79	1.01	1.28	0.59	1.11	0.09	0.65	0.87	1.07	1.22	0.72	1.78	0.98	0.92	0.64	0.98	0.39	0.87
IM 3 18h 17.12.09	1.01	2.28	0.02	0.36	0.4	1.88	0.69	1.05	0.68	0.95	1.25	0.01	0.76	0.89	0.99	1.43	1.11	3.05	0.64	1.09	0.99	1.26	0.75	0.81
IM 4 18h 26.11.09	0.82	0.89	0.33	2.35	0.4	0.98	1.01	1.03	1.28	0.64	1.18	0.19	0.9	1.17	1.89	1.2	0.55	2.3	2.49	1.1	1.65	0.73	0.26	1.19
IM 5 18h 26.11.09	1	0.88	0.02	0.09	0.22	2.41	0.75	0.87	0.32	0.85	0.63	0.01	0.87	1.07	1.15	1.33	0.49	11.3	1.3	1.61	0.56	1.1	0.24	1
IM 1 30h 08.01.10	0.92	1.28	9.07	1.03	0.82	1.25	1.04	0.82	0.8	0.73	1.25	3.86	1.15	1.17	1.13	0.96	1.46	1.36	0.9	1.14	0.62	1.02	1.34	1.05
IM 2 30h 08.01.10	0.92	1.51	0.72	0.86	0.65	2.4	0.94	0.64	0.81	0.96	1.16	0.67	0.93	1.21	0.97	1.22	1.56	3.47	1	1.21	1.19	1.29	1.21	0.95
IM 3 30h 08.01.10	0.64	1.51	0.14	0.98	0.55	1.92	0.76	0.46	1.82	0.78	1.01	0.35	0.63	0.98	0.83	1.58	1.57	0.57	1.12	0.79	0.62	1.03	0.93	0.94
IM 3 30h 15.12.09	1.06	0.93	0.02	1.71	0.72	0.98	0.91	0.46	1.7	0.68	1.16	0.03	0.7	0.91	0.89	1.06	1.59	0.46	0.96	0.95	0.92	0.82	0.9	0.57
IM 4 30h 15.12.09	1.17	1.21	3.28	0.6	0.17	1.9	0.93	0.59	0.72	0.91	1.26	0.03	1.32	1.17	1.01	1.51	1.5	1.36	0.96	1.07	0.65	1.18	1.31	1.04
IM 1 2d 03.12.09	0.54	1.58	0.12	0.22	0.74	0.9	0.98	1.08	1.5	0.66	1.1	0.07	0.59	0.97	0.88	1.28	2.24	0.61	1.3	1.08	1.09	0.73	2.42	0.91
IM 2 2d 03.12.09	0.47	1.33	0.09	3.05	0.41	1.47	1.14	0.54	1.44	0.45	0.92	0.07	0.64	1.09	1.89	1.52	1.93	0.41	2.04	1.24	1.42	0.5	1.91	1.1
IM 3 2d 10.12.09	0.78	1.76	5.56	1.3	0.14	1.05	0.99	1.04	1.59	0.75	0.83	0.04	0.55	0.85	0.97	0.95	2.17	0.43	0.75	1.13	0.52	0.92	2	1.09
IM 4 2d 10.12.09	0.74	1.43	0.44	0.45	0.4	2.17	0.83	0.44	0.97	1.58	0.88	0.34	0.96	0.88	1.05	1.73	1.13	2.82	0.59	0.89	0.74	1.27	0.99	1.2
IM 5 2d 10.12.09	0.9	1.1	0.15	0.45	0.41	1.56	0.95	0.79	0.95	1.15	0.96	0.14	0.84	1.13	1.14	1.49	1.48	0.65	0.84	1.34	1.05	1.01	1.89	1.46
IM 1 5d 21.04.10	0.6	1.44	0.04	0.61	0.27	0.8	0.81	0.51	0.64	0.59	0.97	0.03	0.89	0.91	0.8	0.79	1.66	0.86	1.04	0.94	0.49	0.56	1.61	0.96
IM 2 5d 21.04.10	0.65	1.5	0.04	3.5	0.67	0.63	1.06	0.39	1.41	0.65	1.06	12.2	0.92	1.23	0.99	1.17	1.77	0.09	2.18	1.43	1.48	0.38	2.3	0.92
IM 3 5d 21.04.10	0.51	0.86	0.44	1.59	0.21	0.38	0.78	0.5	0.62	0.43	0.95	0.39	0.71	0.85	0.68	0.62	1.42	0.1	1.03	0.9	0.58	0.39	1.47	0.83
IM 4 5d 07.12.09	0.9	0.95	7.57	1.73	1.33	0.77	0.95	0.64	1.72	0.65	0.7	6.58	1	0.97	0.64	0.93	1.92	0.2	0.97	0.85	0.34	0.59	2.79	0.85
IM 5 5d 21.04.10	1.27	1.09	0.46	1.53	52.5	2.09	1.51	0.74	2.2	1.68	1.01	659.5	1.41	1.57	1.14	1.27	1.62	1.7	1.82	1.9	3.49	0.64	2.41	1.04
IM 4 5d 21.04.10	n.m.	n.m.	n.m.	n.m.	n.m.	n.m.	n.m.	n.m.	n.m.	n.m.	n.m.	n.m.	n.m.	n.m.	n.m.	n.m.	n.m.	n.m.	n.m.	n.m.	n.m.	n.m.	n.m.	n.m.
IM 1 14d 09.12.09	1.07	0.86	0.77	4.73	78.7	2.09	1.67	0.59	3.42	1.87	0.62	1257	1.19	1.15	1.07	1.1	2.14	0.42	1.08	1.32	2.83	0.83	4.8	1.34
IM 2 14d 09.12.09	1.16	0.79	0.18	4.92	14.4	1.5	1.62	0.44	3.12	1.06	0.49	204.1	1.12	0.93	0.9	0.91	1.59	0.15	1.21	1.57	1.73	0.8	2.91	1.11
IM 3 14d 09.12.09	0.91	0.9	7.38	2.84	0.99	1.13	0.93	0.48	1.56	1.06	0.6	5.95	0.79	0.77	0.77	1.11	1.76	0.14	0.71	1.27	1.18	0.9	2.96	1.05
IM 1 14d 16.12.09	0.7	1.2	0.04	2.58	0.26	0.52	0.92	0.54	1.1	0.6	0.6	2.97	1.01	0.92	0.67	0.68	1.26	0.13	1.07	1.2	1.11	0.58	2.11	0.77
IM 2 14d 16.12.09	0.51	0.8	1.76	2.94	0.23	0.28	0.73	0.57	0.66	0.4	0.77	0.01	0.65	0.79	0.6	0.61	1.72	0.05	0.93	1.04	1.05	0.37	1.97	0.56

ID	Socs3	Vdr	Nr2f2	Rxra	Cyp7a1	Ugt1a1	Socs1	Abcg2	Abcc2	Por	Cyp2c37	Nr1i2	Cebpb	Nr0b2	Cyp2b10	Nr2f1	Cyp2d22	Actb	Hnf4a	Hk2	Ptgs2	Nr1i3	Cyp2c29
IM 1 0h 17.12.09	0.56	0.38	0.9	0.9	1.02	1.13	0.61	0.93	0.71	0.57	2.35	0.57	0.61	0.15	0.99	0.55	1.17	0.64	0.9	0.24	0.34	0.72	2.11
IM 2 0h 17.12.09	0.62	2.33	1.55	1.26	1.58	1.63	1.57	1.35	1.28	0.69	3.18	0.88	0.73	21.9	1.33	2.76	1.63	0.71	1.43	1.01	2.33	1.02	2.37
IM 3 0h 06.01.10	0.29	0.23	1.42	0.98	2.02	1.23	0.33	1.03	0.99	0.89	1.58	0.76	0.7	0.34	0.59	0.71	1.04	0.75	1.03	0.46	0.18	0.77	1.62
IM 4 0h 06.01.10	0.64	19.8	2.02	1.39	4.38	1.67	3.09	1.5	1.79	1.22	2.03	1.1	0.94	0.15	2.94	8.46	1.51	0.6	1.12	3.03	20.1	1.75	1.35
IM 5 0h 06.01.10	0.56	0.16	0.83	1.03	4	0.93	0.65	0.95	0.95	1.22	0.93	0.65	0.78	0.11	0.76	0.38	0.8	0.59	0.74	0.41	0.06	0.82	1.06
IM 2 6h 11.12.09	3.53	59.2	1.64	1.56	0.72	1.31	8.68	1.78	1.14	1.14	1.71	1.69	1.6	496.5	18.3	26.7	1.27	0.96	1.82	6.15	46.2	1.18	2
IM 3 6h 11.12.09	4.39	71.8	1.37	1.43	0.55	1.35	13.1	1.74	0.95	0.86	2.18	1.51	1.62	655.5	19.1	29.5	1.27	1.06	1.56	6.99	58.5	1.03	1.96
IM 4 6h 11.12.09	2.63	26.2	1.07	1.19	0.29	1.4	3.9	1.43	1.03	1.51	1.66	2.01	1.68	193.2	7.92	10.6	1.32	0.89	1.51	3.4	23.2	0.83	1.77
IM 3 6h 01.12.09	1.25	0.43	0.69	1.02	0.51	0.94	1.08	0.87	0.6	0.8	1.74	0.65	0.78	228.5	2.2	0.5	0.97	0.75	0.97	0.35	0.6	0.56	2.15
IM 4 6h 01.12.09	0.97	0.05	1.02	1.12	0.85	1.22	0.94	1.05	0.88	0.95	2.24	0.86	0.61	451.4	0.91	0.62	1.18	0.64	1.15	0.27	0.18	0.66	2.31
IM 1 12h 08.12.09	1.14	0.59	1.1	0.97	0.67	1.11	0.64	0.78	0.88	1.3	1.23	1.29	0.87	1.15	1.81	0.34	0.86	0.88	1.04	0.59	0.6	0.92	1.63
IM 2 12h 08.12.09	0.68	0.49	1.09	0.91	1.8	1.08	0.6	0.89	1.05	1.12	1.2	1.02	0.71	1.32	0.65	0.49	0.9	0.64	0.87	0.54	0.45	1.05	1.46
IM 1 12h 11.12.09	0.99	0.24	0.72	0.98	1.03	0.78	0.52	0.7	0.68	1.14	1.21	1.07	0.84	0.43	4.08	0.28	0.72	1.08	0.7	0.29	0.07	1.21	1.29
IM 1 12h 14.12.09	0.77	0.41	1	1.09	0.79	1.09	0.66	0.68	0.87	1.39	1.31	1.43	1.05	1.41	1.32	0.57	0.76	0.74	1.33	0.46	0.28	1.46	1.34
IM 2 12h 14.12.09	1.4	0.6	0.65	0.99	0.24	0.78	1.54	0.65	0.51	0.65	1.03	0.93	0.95	1.05	2.37	0.58	0.68	0.96	0.86	0.38	0.2	0.77	1.37
IM 1 18h 17.12.09	1.09	0.52	0.98	0.9	0.6	1.32	0.56	0.97	1.18	1.44	1.85	1.28	1.24	0.66	0.82	0.55	1.02	1.12	1.13	0.64	2.37	1.43	2.03
IM 2 18h 17.12.09	1.3	1.13	0.84	0.86	0.49	1.13	0.53	0.76	0.66	1.01	1.21	1.06	1.01	0.16	0.4	0.26	0.81	1.17	0.96	0.4	0.39	1.26	0.97
IM 3 18h 17.12.09	0.73	0.41	0.7	0.93	0.95	1.12	0.65	0.89	0.89	1.05	1.28	0.89	0.99	0.46	1.51	0.3	1.07	1.02	1.05	0.35	0.44	1.44	1.38
IM 4 18h 26.11.09	2.57	0.72	0.69	1.19	0.21	0.92	1.71	0.81	0.91	0.96	1.4	0.98	1.63	0.85	0.36	0.4	0.85	1.27	1.25	0.69	0.79	1.17	0.99
IM 5 18h 26.11.09	1.97	0.12	0.68	1.26	1.89	1.05	0.35	0.9	0.86	0.68	2.11	0.6	0.95	0	0.38	0.25	0.89	0.98	0.99	0.34	0.04	1.8	0.84
IM 1 30h 08.01.10	1.69	0.65	0.81	1.07	1.27	1.14	0.63	1.35	1.27	0.92	1.51	1.06	1	1.74	0.24	0.57	0.94	1.06	1.3	0.84	0.71	1.42	1.2
IM 2 30h 08.01.10	1	0.92	0.79	1.09	2.55	1.27	0.75	1.07	1.28	1.15	1.14	1.11	1.12	0.27	0.96	0.66	1.1	0.96	1.12	1.06	2.11	1.88	0.56
IM 3 30h 08.01.10	1.2	0.96	0.86	0.93	0.08	1.23	0.43	0.77	0.92	1.49	1.01	1.48	1.18	0.77	0.89	0.56	1.03	0.83	1.16	0.61	1.01	1.13	0.63
IM 3 30h 15.12.09	1.25	0.3	1.06	0.93	0.22	1.01	0.47	0.78	1.25	1.25	0.88	1.17	1.27	0.44	0.4	0.66	0.75	1.24	0.91	0.79	0.59	1.36	0.66
IM 4 30h 15.12.09	1.44	1.01	1.13	1.25	1.47	1.13	0.81	0.91	1.28	1.19	1.53	1.02	1.39	0.0005	0.46	0.29	1.24	0.97	1.26	0.94	0.16	2.01	0.94
IM 1 2d 03.12.09	1.76	0.2	0.97	0.91	0.32	0.9	0.55	1.02	1.14	0.87	0.86	1.14	1.13	1.22	0.38	0.56	0.84	1.2	0.97	0.91	0.38	0.83	1.5
IM 2 2d 03.12.09	2.46	0.66	0.75	0.95	0.03	1.17	2.3	0.95	0.91	0.8	1.12	1.1	1.4	0.58	0.25	0.79	0.75	1.81	1.19	1.44	1.12	0.74	0.87
IM 3 2d 10.12.09	0.27	0.94	0.78	0.65	1.89	1.24	0.32	1.28	1.04	0.76	1.14	0.87	0.79	0.94	0.62	0.73	0.96	1.36	0.82	1.26	0.68	1.01	1.53
IM 4 2d 10.12.09	0.63	0.38	0.82	0.87	1.27	1.2	0.37	1.03	0.95	1.73	1.05	1.21	0.72	0.55	6.55	0.49	1.32	0.59	1.02	0.31	0.12	1.52	0.6
IM 5 2d 10.12.09	1.08	0.33	0.75	1	1.51	1.13	0.34	1.31	1.08	1.18	0.97	1.1	1.15	0.31	0.86	0.67	1.15	0.91	1.09	0.69	0.1	1.67	0.95
IM 1 5d 21.04.10	0.3	0.47	0.84	0.88	2.12	0.58	0.4	0.95	0.8	0.74	0.53	0.82	0.85	0.11	0.41	0.79	0.84	0.92	0.8	1.3	6.58	0.75	0.73
IM 2 5d 21.04.10	1.69	1.06	1.21	0.83	0.64	0.53	0.96	0.97	0.96	0.65	0.22	1.07	1.44	0.25	0.24	1.58	0.91	2.44	0.88	2.39	2.13	0.49	0.6
IM 3 5d 21.04.10	0.39	0.81	0.81	0.66	1.16	0.39	0.4	0.93	0.88	0.44	0.24	0.75	0.86	0.13	0.12	0.97	0.83	1.13	0.55	1.69	1.05	0.5	0.36
IM 4 5d 07.12.09	0.55	1.22	1.12	0.78	3.49	0.58	0.51	0.8	1.07	0.95	0.32	0.88	0.63	0.95	1.27	1.39	1	0.98	0.62	1.35	0.74	0.72	0.44
IM 5 5d 21.04.10	1.45	8.8	1.62	1.41	1.82	0.7	4.29	1.39	1.37	1.11	0.72	1.37	1.73	76.2	3.2	5.51	1.28	1.42	1.29	3.64	16.4	1.26	0.5
IM 4 5d 21.04.10	n.m.	n.m.	n.m.	n.m.	n.m.	n.m.	n.m.	n.m.	n.m.	n.m.	n.m.	n.m.	n.m.	n.m.	n.m.	n.m.	n.m.	n.m.	n.m.	n.m.	n.m.	n.m.	n.m.
IM 1 14d 09.12.09	1.2	15.1	1.55	1.25	4.32	1.31	3.93	1.28	1.51	1.71	0.7	0.98	0.94	130.6	2.6	8.19	1.41	1.22	1.03	4.72	15.5	1.35	0.41
IM 2 14d 09.12.09	1.15	6.04	1.61	1.1	3.6	1.02	3.12	1.07	1.41	1.51	0.53	0.93	1.17	21.4	0.63	4.42	1.07	1.67	0.9	5.18	8.36	0.89	0.45
IM 3 14d 09.12.09	0.75	0.68	1.06	0.84	5.42	0.96	0.88	0.85	1.39	1.67	0.39	0.93	0.92	0.71	0.47	1.31	1.1	0.85	0.77	1.8	1.59	1.01	0.44
IM 1 14d 16.12.09	0.69	1.24	1.31	0.87	3.29	0.69	2.61	0.93	0.98	0.88	0.26	0.85	1.02	0.004	0.53	1.11	0.94	1.82	0.75	2.94	1.62	0.51	0.64
IM 2 14d 16.12.09	0.41	2	0.95	0.62	1.85	0.51	3.95	0.96	0.96	0.53	0.13	0.56	0.75	0.17	0.4	0.82	0.77	1.49	0.54	3.27	2	0.34	0.36

3 RNA Fibrosis genes

ID	Pparg	Edn1	Birc5	Sparc	Col1a1	Col8a1	Igf1	Fasl	Bad	Tgfbr2	Rarres1	Lama1	Timp1	Nes	Bcl2l11	Pdgfb	Xiap	Notch3	Cdh1	Timp2	Wisp1	Bak1	Gdf2	Pde4a
IM 1 0h 17.12.09	1.59	0.52	0.63	0.77	0.48	2.1	1.14	1.07	0.7	0.79	1.26	1.83	0.06	1.28	0.93	0.63	1.13	1.35	0.69	1.01	2.26	0.63	1.51	0.87
IM 2 0h 17.12.09	0.59	0.79	0.47	0.68	0.18	1.1	1.07	0.18	0.65	0.63	1.21	1.82	0.07	1.55	0.73	0.44	1.03	1.11	0.76	0.9	1.74	0.65	1.84	1.11
IM 3 0h 06.01.10	0.79	0.24	0.7	0.81	0.19	1.11	0.96	1.88	0.76	0.66	1.46	1.83	0.09	1.18	0.67	0.42	1.33	1.24	0.81	0.8	1.81	0.81	1.7	0.95
IM 4 0h 06.01.10	0.75	0.6	0.58	0.91	0.65	1.33	1.08	1.89	0.67	0.95	1.08	1.74	0.08	1.34	0.69	0.46	0.88	1.74	0.63	1.06	1.73	0.73	1.77	0.92
IM 5 0h 06.01.10	0.64	0.38	0.65	0.68	0.54	1.16	0.83	0.45	0.72	0.74	1.32	1.54	0.11	0.88	0.54	0.37	0.92	1.02	0.66	1.1	1.04	0.66	2.05	1.37
IM 2 6h 11.12.09	0.62	0.92	0.92	0.95	0.36	0.53	0.88	0.93	0.59	0.66	2	2.16	0.36	0.42	0.61	0.6	0.76	0.87	0.57	0.92	1.4	0.73	2	0.65
IM 3 6h 11.12.09	0.53	0.82	0.23	0.75	0.18	0.57	0.94	0.58	0.62	0.69	1.57	1.16	0.26	0.35	0.77	0.58	0.76	0.74	0.44	0.57	1.03	0.69	1.77	0.32
IM 4 6h 11.12.09	0.61	0.76	0.46	0.72	0.32	0.37	1.02	1.43	0.8	0.82	2.61	1	0.34	0.27	0.65	0.51	0.66	0.61	0.57	0.66	1.17	0.74	1.35	0.42
IM 3 6h 01.12.09	0.7	0.93	0.59	0.68	0.24	0.57	0.82	2.41	0.86	0.72	1.55	1.03	0.42	0.71	1.16	0.67	0.95	0.74	0.72	0.73	1.84	0.9	1.05	0.75
IM 4 6h 01.12.09	0.49	0.73	0.28	0.59	0.25	0.41	1.17	0.45	0.57	0.64	1.26	0.75	0.13	0.39	0.72	0.55	0.89	0.63	0.3	0.6	0.34	0.64	1.22	0.43
IM 1 12h 08.12.09	0.89	0.5	0.46	0.5	0.22	0.29	0.87	1.36	1.48	0.96	0.79	0.55	1.06	0.27	1.42	0.73	1	0.38	1.05	0.67	0.73	0.87	1.14	0.98
IM 2 12h 08.12.09	0.84	0.96	0.38	0.62	0.42	0.71	0.87	1.03	1.26	0.78	0.84	0.82	0.54	0.7	1.24	0.72	0.91	0.52	0.83	0.74	1.06	0.75	1.31	0.9
IM 1 12h 11.12.09	0.92	0.36	0.33	0.26	0.07	0.11	0.83	0.9	1.04	0.79	0.73	0.15	0.47	0.35	0.94	0.51	1.02	0.23	0.79	0.3	0.17	0.89	0.48	0.56
IM 1 12h 14.12.09	1.31	0.78	0.54	0.47	0.17	0.4	1.15	2.14	1.56	0.9	1.79	0.64	0.47	0.65	1.8	0.88	0.99	0.61	0.81	0.55	1.04	1.08	0.57	0.75
IM 2 12h 14.12.09	1.04	0.64	0.32	0.51	0.15	0.76	1.05	0.75	1.43	0.77	2.59	0.48	0.92	0.83	1.45	0.76	1.11	0.59	0.99	0.46	1.38	0.93	0.72	0.76
IM 1 18h 17.12.09	1.32	2.03	0.48	0.86	1.11	1.3	1.15	1.5	1.54	1.19	1.37	0.44	1.75	1.73	1.92	1.6	1.14	1.26	1.54	0.95	1.51	1.15	0.52	0.95
IM 2 18h 17.12.09	0.96	0.68	0.22	0.26	0.18	0.12	1.05	0.38	1.29	0.6	0.89	0.18	0.66	0.29	0.7	0.53	0.99	0.17	1.07	0.33	0.43	0.63	0.33	0.68
IM 3 18h 17.12.09	0.79	0.73	0.29	0.24	0.17	0.02	1.06	0.16	0.88	0.59	1.24	0.18	0.72	0.49	0.96	0.41	1.08	0.18	0.87	0.37	0.19	0.78	0.29	0.7
IM 4 18h 26.11.09	0.68	0.87	0.11	0.33	0.11	0.21	1.28	1.16	1.16	0.77	1.06	0.24	0.47	0.3	1.56	0.75	1.19	0.17	0.83	0.34	0.56	0.94	0.43	0.82
IM 5 18h 26.11.09	1.18	0.31	0.14	0.24	0.11	0.14	1.16	0.41	1.1	0.44	1.25	0.31	0.24	0.18	0.88	0.47	1.18	0.17	0.51	0.34	0.06	0.71	0.39	0.73
IM 1 30h 08.01.10	1.3	0.42	0.93	0.49	0.68	0.12	1.19	0.19	0.95	0.65	0.88	0.24	1.05	0.84	0.78	0.73	1.05	0.29	0.94	0.56	0.68	0.77	0.38	0.64
IM 2 30h 08.01.10	1.54	1.24	1.88	0.8	1.38	1	1.02	4.34	1.48	1.02	1.13	0.8	2.03	1.34	1.26	1.28	1.15	1.14	1.59	1.16	2.29	1.2	0.94	1.44
IM 3 30h 08.01.10	0.87	0.82	1.17	0.85	1.06	1.39	1.14	1.17	1.34	0.98	0.88	0.64	1.64	0.56	0.79	0.66	0.9	0.9	1.5	0.76	0.78	1.07	1.25	1
IM 3 30h 15.12.09	0.98	1.25	1.01	1.58	2.84	2.47	0.81	1.76	1.47	1.43	0.71	1.82	3.51	2.14	1.44	1.36	1.2	2.4	1.92	1.03	1.34	1.21	1.62	0.98
IM 4 30h 15.12.09	0.86	0.38	0.65	0.33	0.66	0.09	1.12	0.52	1	0.74	0.52	0.32	1.24	0.17	0.8	0.66	1.09	0.51	0.84	0.4	0.19	1.13	0.58	1.56
IM 1 2d 03.12.09	0.89	1.11	4.59	1.67	2.93	0.94	1.1	1.12	0.77	1.1	0.48	2.66	1.62	1.89	0.6	1.3	0.98	1.35	1.35	1.07	1.48	1.25	1.81	1.48
IM 2 2d 03.12.09	1.18	1.56	2.77	1.59	5.1	1.91	1.21	0.95	1.05	1.18	0.34	1.06	4.22	1.91	0.85	1.61	1.2	1.23	1.35	1.12	1.78	1.34	0.63	1.23
IM 3 2d 10.12.09	1.3	2.03	4.74	1.73	3.32	3.66	1.14	0.08	1.18	1.24	0.79	1.36	4.85	3.85	0.17	1.35	1.15	0.68	2.64	2.49	0.001	1.26	0.001	0.31
IM 4 2d 10.12.09	0.78	0.71	0.44	0.44	1.24	0.34	0.95	0.48	0.92	0.78	0.97	0.23	0.73	0.47	0.63	0.54	0.88	0.31	0.82	0.49	0.17	0.75	0.38	0.61
IM 5 2d 10.12.09	1.52	0.9	1.99	1.05	2.54	0.58	1.53	1.13	1.5	0.95	0.57	0.85	0.93	1.04	0.66	0.96	1.21	0.55	1.7	0.8	0.73	1.17	0.87	1.38
IM 1 5d 21.04.10	1.17	1.7	5.37	2.58	5.67	3.94	0.86	0.57	0.9	1.22	0.95	1.63	5.7	1.71	1.39	1.87	0.91	2.4	0.9	1.94	2.44	1.05	2.96	1.48
IM 2 5d 21.04.10	1.28	4.16	14.4	6.03	19.1	12.5	1.1	2.81	1.66	2.22	0.76	5.45	11.3	6.01	1.72	4.85	1.78	8.79	2.72	4	7.78	1.92	3.2	3.52
IM 3 5d 21.04.10	0.84	2.1	3.91	3.13	6.89	6.15	0.85	1.08	0.77	1.59	0.77	3.24	5.38	4.14	1.05	2.37	0.98	3.44	1.27	2.05	3.65	1.25	2.48	2.03
IM 4 5d 07.12.09	0.94	1.28	4.63	2.72	7.12	2.97	1.01	0.84	0.78	1.53	0.59	2.06	2.32	2.81	1.04	1.9	0.83	2.31	1.18	1.96	2.75	1.17	2.01	1.59
IM 5 5d 21.04.10	1.2	2.55	6.67	3.1	7.86	4.75	0.94	1.03	1.11	1.34	1.06	3.78	3.69	1.52	1.32	2.08	1.22	3.01	1.13	2.49	2.28	1.37	3.67	1.67
IM 4 5d 21.04.10	n.m.	n.m.	n.m.	n.m.	n.m.	n.m.	n.m.	n.m.	n.m.	n.m.	n.m.	n.m.	n.m.	n.m.	n.m.	n.m.	n.m.	n.m.	n.m.	n.m.	n.m.	n.m.	n.m.	n.m.
IM 1 14d 09.12.09	1.38	2.34	0.84	3.24	9.91	6.71	0.94	1.3	1	1.97	0.86	1.58	4.49	1.54	1.98	2.02	0.69	3.22	0.99	3.83	3.17	1.11	2.39	1.13
IM 2 14d 09.12.09	2.18	3.51	3.3	6.8	6.66	14.4	0.79	5.86	1.12	2.91	0.92	3.23	11.7	4.32	2.89	5.13	0.9	7.47	1.53	6.34	8.86	1.64	3.56	2.04
IM 3 14d 09.12.09	1.46	3.01	1.13	2.41	9.22	4.31	1.05	1.12	0.83	1.75	0.8	1.34	3.41	2.16	1.17	2.66	0.69	2.86	0.93	2.61	2.58	1.12	2.63	1.29
IM 1 14d 16.12.09	2.35	3.53	9.25	6.58	21.6	18.2	0.79	9.42	1.38	2.46	1.04	5.2	10.5	4.11	2.54	5.01	1.07	7.2	2.03	4.72	7.59	2.39	3.9	2.67
IM 2 14d 16.12.09	1.55	2.39	9.07	4.36	14	8.21	0.62	9.9	0.94	2.3	0.85	3.3	8.92	4.07	1.52	3.62	0.92	5.02	1.87	2.69	6.09	2.54	2.52	2.81

ID	Ctgf	Notch1	Tnc	Bax	Cdh2	Pde4b	Fn1	Smad7	Mki67	Prom1	Ch25h	Pde4d	Acta2	Pten	Tgfb2	Mmp10	Col4a3	Actb	Col3a1	Cyp2e1	Rps18	Smad6	Col6a6
IM 1 0h 17.12.09	0.59	0.94	0.96	0.65	1.13	1.44	0.6	1.76	0.48	3.33	0.31	1.1	1.96	1.07	0.4	0.002	1.75	0.69	0.86	1.9	0.88	1.13	1.02
IM 2 0h 17.12.09	0.45	0.99	0.5	0.56	1.15	1.07	0.66	1.06	0.43	0.37	0.42	0.48	1.84	1.03	0.45	0.001	2.05	0.61	0.48	1.93	0.75	1.15	0.34
IM 3 0h 06.01.10	0.58	0.85	0.29	0.74	1	0.66	0.69	0.9	0.44	0.33	0.09	1.01	0.18	0.98	0.29	0.002	1.11	0.89	0.5	1.79	1.03	0.9	0.17
IM 4 0h 06.01.10	0.82	0.76	0.74	0.56	0.9	0.54	0.59	1.06	0.38	0.68	0.13	0.49	1.11	0.84	0.6	0.001	1.86	0.48	1.03	1.67	0.69	0.7	1.3
IM 5 0h 06.01.10	0.62	0.7	0.66	0.56	0.96	0.77	0.66	0.78	0.61	0.36	0.87	0.57	2.78	0.87	0.4	0.001	1.57	0.53	0.89	1.22	0.76	0.67	0.37
IM 2 6h 11.12.09	1.7	0.96	0.4	0.65	0.76	0.3	1.21	1.27	0.96	0.23	0.95	1.52	2.52	0.77	0.55	0.01	1.76	0.75	0.51	1.2	0.84	1.02	0.48
IM 3 6h 11.12.09	1.34	0.57	0.22	0.58	0.51	0.48	0.91	1.04	0.23	0.16	0.85	0.86	0.65	0.63	0.59	0.002	0.19	0.78	0.29	1.08	0.79	0.77	0.58
IM 4 6h 11.12.09	1.56	0.61	0.41	0.51	0.59	0.67	0.97	1.91	0.44	0.18	0.75	0.65	1.05	0.7	0.51	0.01	0.99	0.66	0.37	1.23	0.79	0.72	0.77
IM 3 6h 01.12.09	1.28	0.92	0.59	0.66	0.69	0.64	1.16	1.44	0.47	0.89	2.04	1.01	3.07	0.82	1.05	0.17	1.77	0.91	0.23	1.24	0.73	0.83	1.17
IM 4 6h 01.12.09	1.03	0.68	0.14	0.55	0.65	0.52	1.01	0.9	0.34	0.1	0.47	0.94	0.39	0.8	0.37	0.09	1.4	0.74	0.32	1.97	0.81	0.84	0.35
IM 1 12h 08.12.09	0.67	0.79	0.37	1.16	0.84	1.45	1.47	1.31	0.28	0.94	0.67	1.08	0.72	1.04	0.87	0.004	0.98	0.92	0.22	1.12	1.05	1.02	0.72
IM 2 12h 08.12.09	0.71	0.7	0.73	0.88	0.76	1.19	1.13	0.83	0.29	0.49	1.35	0.79	0.79	0.93	0.95	0.01	1.07	0.76	0.41	1.43	1.03	0.65	0.81
IM 1 12h 11.12.09	0.34	0.55	0.07	0.88	0.65	0.54	1.33	1.2	0.16	0.27	0.56	1.13	0.27	0.83	0.5	0.05	0.19	1.18	0.05	0.72	0.89	0.64	0.98
IM 1 12h 14.12.09	0.69	0.8	0.22	0.94	0.89	1.34	1.37	1.3	0.37	0.51	0.96	0.91	1.02	1.06	0.95	0.01	0.63	0.75	0.16	1.39	0.99	0.7	1.09
IM 2 12h 14.12.09	0.61	0.75	0.26	1.13	0.7	0.67	1.67	1.32	0.27	0.43	0.49	0.93	0.23	0.88	0.81	0.05	1.64	1.13	0.15	0.81	0.96	0.79	1.58
IM 1 18h 17.12.09	1.07	1.22	1.09	1.36	1.38	1.99	1.43	1.9	0.75	2.02	3	1.31	2.42	1.3	0.76	382.2	0.0002	1.38	1.13	1.81	1.57	1.32	0.97
IM 2 18h 17.12.09	0.42	0.72	0.19	1.01	1.04	0.68	1.51	0.96	0.15	0.34	0.54	0.63	0.92	0.95	0.39	72	0.63	1.23	0.12	1.29	1.29	0.54	0.63
IM 3 18h 17.12.09	0.33	0.69	0.28	0.86	1.03	0.44	1.21	0.95	0.15	0.19	1.28	1.21	2.16	1.07	0.37	363.5	0.34	1.09	0.12	1.24	0.93	0.65	0.18
IM 4 18h 26.11.09	0.4	0.66	0.24	1.1	0.91	3.98	1.67	1.66	0.17	0.53	1.66	1.18	0.3	1.16	0.45	4971	0.28	1.34	0.15	1.53	1.21	1.28	1.13
IM 5 18h 26.11.09	0.32	0.65	0.18	0.86	1.02	0.52	1.32	0.78	0.18	0.32	0.31	0.98	0.24	1.03	0.34	3286	0.32	1.07	0.09	1.98	1.16	0.57	0.64
IM 1 30h 08.01.10	0.34	0.55	0.6	1.05	1.07	1.45	0.97	0.82	1.28	0.61	0.73	1.05	0.76	1.08	0.42	4.38	0.52	1.04	0.52	1.73	1.15	0.53	0.37
IM 2 30h 08.01.10	1.23	1.16	4.15	1.03	1.15	1.1	0.99	1.35	2.07	2.17	1.88	1.22	2.95	1.1	1.33	0.05	5.39	1.02	1.05	1.4	1.15	0.97	2.27
IM 3 30h 08.01.10	1	0.79	0.88	1.03	1.01	0.87	1.11	2.15	1.01	0.84	0.89	0.64	1.01	1.05	0.85	7.34	0.72	0.88	1.08	1.15	1.19	0.88	1.28
IM 3 30h 15.12.09	1.15	1.1	3.15	1.53	0.99	1.38	1.09	1.47	1.28	1.39	3.61	1.09	1.06	1.2	1.54	2.81	3.46	1.33	2.86	1.37	1.39	1.11	2.43
IM 4 30h 15.12.09	0.23	0.88	0.1	1.01	1.06	0.63	1.13	0.77	1.11	0.41	0.63	1.07	1.1	1.25	0.49	5.86	0.38	0.97	0.44	1.99	1.4	0.65	0.27
IM 1 2d 03.12.09	0.85	1.65	2.29	1.6	1.28	0.54	0.93	1.33	5.72	2.38	1.14	1.33	0.88	1.18	0.71	3.11	1.09	1.26	2.12	0.76	1.05	1.03	1.5
IM 2 2d 03.12.09	1.13	1.27	2.89	1.77	0.93	2.32	1.33	1.53	3.95	2.7	2.82	1.11	1.27	1.2	1.19	24.8	0.92	1.84	3.38	0.82	1.31	1.25	2.01
IM 3 2d 10.12.09	0.45	2.36	2.84	1.02	3.2	1.63	0.91	0	5.35	1.45	0.4	0.45	0.0002	1.12	1.16	27.7	0	1.34	0.81	0.84	1.13	1.28	0.47
IM 4 2d 10.12.09	0.61	0.66	0.61	0.54	0.96	0.55	0.87	0.97	0.38	0.56	0.37	0.87	1.16	0.97	0.68	10.5	1.46	0.58	0.66	1.47	0.79	0.72	0.26
IM 5 2d 10.12.09	0.67	1.11	0.77	1.14	1.17	0.75	1.19	0.99	2.26	1.37	0.33	1.11	0.65	1.27	0.97	22.3	0.27	0.91	1.43	1.78	1.2	0.76	1.01
IM 1 5d 21.04.10	2.1	1.22	4.47	1.38	1	1.26	0.62	1.99	5.3	2.57	1.4	0.8	1.22	0.9	2.04	14.7	2.25	0.9	5.89	0.6	0.79	1.55	2.09
IM 2 5d 21.04.10	3.34	2.5	15.6	2.63	1.52	2.3	1.47	2.85	15.3	14.9	6.52	2.37	6.61	1.59	4.73	25.3	15.3	2.5	19.5	0.42	1.47	2.46	6.33
IM 3 5d 21.04.10	1.39	1.27	6.54	1.32	1.06	1.22	0.7	1.39	5.55	3.24	1.78	1.17	1.86	0.85	2.45	5.21	3.13	1.19	7.83	0.34	0.72	1.34	1.51
IM 4 5d 07.12.09	1.36	1.17	3.33	0.96	0.98	1.05	0.58	1.44	4.53	3.43	1.22	0.82	0.74	0.79	2.23	7.58	6.96	0.87	6.23	0.41	0.74	1.25	2.27
IM 5 5d 21.04.10	2.98	1.92	4.32	1.95	1.3	0.91	1.19	2.31	7.12	2.58	1.99	1.57	5.51	1.14	2.42	2.32	7.26	1.25	7.11	0.69	1.2	1.69	3.36
IM 4 5d 21.04.10	n.m.	n.m.	n.m.	n.m.	n.m.	n.m.	n.m.	n.m.	n.m.	n.m.	n.m.	n.m.	n.m.	n.m.	n.m.	n.m.	n.m.	n.m.	n.m.	n.m.	n.m.	n.m.	n.m.
IM 1 14d 09.12.09	3.57	1.34	4.46	1.01	1.08	1.64	0.67	2.55	1.31	2.92	1.47	1.1	3.44	0.87	4.12	10.6	3.81	0.95	8.55	0.5	1.06	1.96	2.03
IM 2 14d 09.12.09	9.42	1.84	15.2	1.52	1.2	2.45	0.94	3.46	4.41	9.59	5.37	1.35	5.93	1.01	10.1	42.8	20.6	1.54	22.4	0.27	0.97	1.91	4.92
IM 3 14d 09.12.09	3.68	1.41	4.49	1.08	1.22	1.49	0.8	2.08	2.01	4.11	1.91	1.09	2.03	1.06	5.01	13.8	8.77	0.92	7.13	0.5	1.06	1.33	1.45
IM 1 14d 16.12.09	4.73	2.41	15.5	1.95	1.39	2.11	1.04	2.62	9.13	8.2	4.64	2.27	4.5	1.25	6.69	26	14	1.82	20.5	0.39	1.13	2.23	4.93
IM 2 14d 16.12.09	4.71	1.9	10.3	1.79	0.99	1.62	0.74	1.67	7.42	5.86	4.13	1.37	4.46	1.04	5.73	15.6	24.2	1.58	11.4	0.26	0.91	1.64	1.82

4 RNA Zytokine genes

ID	Ccl2	Ifnb1	Cd86	Met	Il28b	Tnfrsf1a	Il1b	Il17a	Cxcl5	Cxcr1	Ifnar1	Osmr	Tgfb1	Ifng	Ccl4	Ccl5	Ccr3	Il1rn	Il13	Tnfrsf1b	Ccl7	Osm	Cd69	Il10rb
IM 1 0h 17.12.09	0.1	0.45	0.44	0.92	33.2	0.52	1.05	0.02	11.7	0.28	0.85	0.21	0.6	0.28	0.29	0.38	0.47	0.29	0.002	0.55	0.12	0.21	0.64	0.58
IM 2 0h 17.12.09	0.27	3.01	0.71	0.94	18.3	0.65	0.59	0.01	0.22	2.25	1.02	0.5	0.82	4.31	0.54	1.3	1.61	0.22	0.001	1.08	0.34	0.44	1.62	0.82
IM 3 0h 06.01.10	0.11	0.05	0.47	0.72	101.9	0.39	0.24	0.03	0.06	0.02	0.77	0.09	0.59	0.67	0.22	0.29	0.26	0.1	0.004	0.83	0.04	0.04	0.49	0.4
IM 4 0h 06.01.10	0.05	0.25	0.38	0.64	122.2	0.4	0.2	0.02	0.01	0.24	0.58	0.19	0.6	0.36	0.18	0.31	0.37	0.11	0.002	0.64	0.07	0.1	0.19	0.47
IM 5 0h 06.01.10	0.05	2.69	0.41	0.72	59.6	0.46	0.37	0.02	0.02	1.8	0.49	0.18	0.55	0.2	0.09	0.29	1.27	0.1	0.002	0.6	0.02	0.11	0.41	0.47
IM 2 6h 11.12.09	0.1	1.82	0.45	0.5	0.004	0.96	0.28	0.01	0.03	1.26	0.71	0.66	0.53	0.2	0.22	0.29	1	0.14	0.002	0.56	0.14	0.15	0.22	0.5
IM 3 6h 11.12.09	0.17	0.53	0.49	0.42	0.004	1.04	0.27	0.01	0.03	0.31	0.68	1.02	0.43	0.24	0.45	0.23	0.26	0.32	0.002	0.4	0.17	0.23	0.18	0.48
IM 4 6h 11.12.09	0.15	0.46	0.32	0.56	0.004	0.99	0.3	0.01	0.05	0.22	0.62	0.63	0.43	0.15	0.21	0.37	0.24	0.2	0.002	0.69	0.13	0.39	0.15	0.35
IM 3 6h 01.12.09	0.21	2.51	0.68	0.69	0.01	1.22	0.39	0.02	0.65	2.14	0.86	1	0.57	0.32	0.4	0.25	1.48	0.49	0.003	0.79	0.31	0.34	0.32	0.49
IM 4 6h 01.12.09	0.16	0.75	0.46	0.96	0.004	1.1	0.32	0.01	0.22	0.38	0.75	0.97	0.47	0.004	0.28	0.3	0.36	0.27	0.002	0.48	0.39	0.25	0.29	0.47
IM 1 12h 08.12.09	1.83	1.39	0.81	0.99	0.004	0.86	1.51	0.01	1.12	1.18	1.03	1.04	0.76	1.13	1.46	1.16	0.67	1.66	240.5	0.87	1.49	2.05	0.74	0.98
IM 2 12h 08.12.09	1.03	0.55	0.57	0.98	0.01	0.62	1.19	0.02	1.16	0.56	0.8	0.46	0.59	0.9	0.66	0.4	0.74	1.22	14.4	0.71	0.98	1.06	0.38	0.74
IM 1 12h 11.12.09	0.36	0.26	0.26	0.82	0.004	0.75	0.29	0.01	0.12	0.36	0.68	0.51	0.31	0.06	0.28	0.18	0.17	0.88	5.84	0.69	0.32	0.38	0.15	0.55
IM 1 12h 14.12.09	0.51	0.45	0.47	1.41	0.004	0.77	0.86	0.01	0.21	0.35	0.84	0.51	0.39	0.38	0.5	0.34	0.39	0.97	10.5	0.75	0.56	0.67	0.36	0.62
IM 2 12h 14.12.09	0.39	1.05	0.41	1.13	0.01	0.8	0.4	0.02	0.22	0.12	0.9	1.35	0.43	0.34	0.22	0.24	0.11	0.82	8.41	0.63	0.44	0.19	0.26	0.7
IM 1 18h 17.12.09	1.97	0.91	0.55	1.21	0.01	0.86	5.31	0.03	3.74	1.05	1.23	0.52	0.69	0.5	5.03	0.61	0.93	1.1	29.1	0.78	3.59	1.56	0.58	0.85
IM 2 18h 17.12.09	0.94	1.33	0.53	0.91	0.005	1.02	0.66	0.02	0.48	0.99	1.02	0.72	0.5	0.47	0.71	0.36	0.72	1.08	6.89	0.74	1.48	0.57	0.23	0.67
IM 3 18h 17.12.09	0.57	1.88	0.39	0.81	0.01	0.72	0.65	0.03	0.2	1.82	0.87	0.23	0.43	0.32	0.4	0.32	1.53	0.47	1.41	0.76	0.55	0.71	0.37	0.49
IM 4 18h 26.11.09	2.67	0.21	0.77	1.22	0.01	1.37	1.48	0.02	1.22	0.45	1.16	1.16	0.64	0.24	0.97	0.38	0.32	7.51	16.3	1.22	1.38	2.07	0.74	0.85
IM 5 18h 26.11.09	0.29	0.14	0.41	0.86	0.01	0.94	0.31	0.02	0.19	0.29	0.85	0.55	0.4	0.35	0.33	0.3	0.24	0.71	7.98	0.82	0.29	0.28	0.32	0.5
IM 1 30h 08.01.10	1.04	0.69	0.87	1.02	0.01	0.99	0.76	0.02	1.23	0.67	0.82	0.35	0.76	0.18	0.52	0.56	0.42	0.84	0.003	0.8	0.67	1.08	0.37	0.74
IM 2 30h 08.01.10	0.97	1.22	1.06	1.03	0.01	0.96	2.14	0.02	3.08	1.47	0.84	0.57	0.86	0.19	0.74	0.81	1.12	0.99	0.003	0.93	1.15	0.31	1.04	0.94
IM 3 30h 08.01.10	1.48	0.37	0.77	0.92	0.004	0.95	0.68	0.01	0.9	0.28	0.79	0.75	0.78	0.15	0.58	0.47	0.37	1.21	0.002	0.9	1.39	0.46	0.14	0.71
IM 3 30h 15.12.09	3.71	0.28	0.82	1.28	0.01	1.18	1.06	0.03	2.21	0.28	1.12	1.46	1.44	0.44	1.2	0.87	0.23	2.3	0.004	1.1	3.09	0.29	0.33	1.3
IM 4 30h 15.12.09	0.97	1.69	0.68	0.87	0.01	1.18	0.41	0.02	0.89	1.26	1.01	0.28	0.74	0.42	0.39	0.5	0.73	0.94	0.002	0.79	0.81	0.09	0.43	0.74
IM 1 2d 03.12.09	1.05	1.09	0.97	0.95	0.01	1.24	0.69	0.04	1.04	0.78	0.98	1.07	1.5	0.42	0.39	0.88	0.76	0.83	0.005	0.85	0.63	0.35	0.74	1.16
IM 2 2d 03.12.09	3.93	0.12	1.06	0.79	0.01	1.62	1.38	0.03	3.09	0.31	1.09	1.81	1.62	0.21	2.22	1.04	0.31	6.52	0.003	1.19	2.46	1.75	0.36	1.36
IM 3 2d 10.12.09	2.45	0.15	1.48	1.25	0.002	0.88	1.02	0.01	2.89	0.62	1.01	1.01	1.47	2.7	1.57	1.96	1.41	1.51	0.001	1.09	1.97	1.17	2.58	1.19
IM 4 2d 10.12.09	0.61	0.75	0.34	0.88	0.004	0.69	0.38	0.01	0.74	0.46	0.61	0.29	0.47	0.15	0.17	0.4	0.35	0.76	0.002	0.6	0.49	0.2	0.2	0.47
IM 5 2d 10.12.09	0.86	0.04	1	1.11	0.01	1.21	0.43	0.03	0.6	0.1	0.93	0.75	0.81	0.32	0.39	0.82	0.45	0.9	0.003	0.88	0.5	0.32	0.56	0.83
IM 1 5d 21.04.10	1.98	0.05	1.69	0.86	$1.3\cdot10^5$	0.9	0.82	28068	4.63	0.22	0.84	1.48	1.57	0.6	1.18	0.67	0.36	1.59	3590	1.02	1.43	1.28	1.07	1.38
IM 2 5d 21.04.10	5.9	0.11	3.57	0.96	$1.6\cdot10^5$	1.29	3.84	$5.6\cdot10^5$	7.53	0.48	1.92	2.39	2.93	0.38	4.58	1.63	1.23	3.32	4384	1.57	4.51	2.52	2.43	2.84
IM 3 5d 21.04.10	7.99	10.6	6.04	1.62	42785	1.67	4.21	$1.7\cdot10^5$	16.4	13.6	1.72	6.13	4.36	85.4	13.7	13.4	11.6	4.42	21087	2.27	13	27.9	27.3	3.72
IM 4 5d 07.12.09	7.66	12.4	5.73	1.94	52813	1.67	3.55	$1.9\cdot10^5$	13.5	14.3	1.51	6.54	3.99	97.2	11.3	14.5	11.4	3.6	25447	2.07	11.2	30.4	28.4	3.31
IM 5 5d 21.04.10	6.64	26.7	5.89	1.73	51020	2.15	5.06	$2.3\cdot10^5$	12.1	26.4	1.8	7.87	3.79	100.9	11.3	13.2	15.3	3.2	29188	2.57	9.71	31.5	28.5	3.44
IM 4 5d 21.04.10	n.m.	n.m.	n.m.	n.m.	n.m.	n.m.	n.m.	n.m.	n.m.	n.m.	n.m.	n.m.	n.m.	n.m.	n.m.	n.m.	n.m.	n.m.	n.m.	n.m.	n.m.	n.m.	n.m.	n.m.
IM 1 14d 09.12.09	9.53	29.3	6.04	1.95	77189	1.71	6.13	$2.9\cdot10^5$	21.3	27.5	1.92	9.15	4.61	163	14.1	19	20.1	3.89	42653	2.44	15.1	45.7	39.4	4.2
IM 2 14d 09.12.09	13.3	25.1	7.82	1.95	$1.3\cdot10^5$	1.95	9.86	$3.8\cdot10^5$	25.4	31.8	1.98	11.8	6.05	195	23.5	32	24.8	5.13	52699	3.12	19.7	56.2	53.7	5
IM 3 14d 09.12.09	7.65	14.6	4.08	1.41	35968	1.34	3.05	$1.5\cdot10^5$	16	16.2	1.49	5.63	3.05	67.5	8.54	10.4	10.5	2.45	17743	2.37	7.87	19.6	19.3	2.86
IM 1 14d 16.12.09	11.4	19.9	7.91	1.49	65051	1.68	7.76	$2.6\cdot10^5$	22.2	21	2.23	8.51	5.33	115.4	18.7	35.9	15	4.53	31449	3.09	13.9	41.6	39	4.79
IM 2 14d 16.12.09	11.3	11.4	6.47	1.1	25608	1.61	6.57	$1.1\cdot10^5$	16.5	12.2	1.69	5.04	4.75	67.3	13.6	21.4	10.2	3.89	12399	2.8	12.4	17.4	19.3	3.69

ID	Il2	Cd14	Cxcr2	Cxcl1	Tnf	Il10ra	Il10	Ccr2	Egf	Hgf	Ifna1	Ifnar2	Cxcl2	Mrc1	Ccr5	Ccl3	Il6	Actb	Il4	Egfr	Ccl8	Cxcl3	Il6st
IM 1 0h 17.12.09	4.15	0.17	0.39	0.04	0.48	0.62	0.16	0.19	0.39	0.6	0.61	0.93	0.06	0.77	0.34	0.3	0.22	0.61	0.23	0.76	0.44	0.09	0.63
IM 2 0h 17.12.09	181.4	0.12	0.27	0.11	0.63	0.91	2.16	0.41	1.49	1.3	3.12	0.91	0.09	1.2	1.13	0.57	0.48	0.6	2.81	1.73	2.65	0.44	0.81
IM 3 0h 06.01.10	29.6	0.06	0.06	0.02	0.16	0.55	0.28	0.1	0.45	0.56	0.02	0.8	0.01	0.68	0.4	0.1	0.11	0.74	0.36	0.88	0.76	0.13	0.56
IM 4 0h 06.01.10	14.6	0.1	0.17	0.06	0.17	0.52	0.19	0.13	0.31	0.46	0.31	0.72	0.02	0.94	0.35	0.11	0.08	0.42	0.38	1.14	0.65	0.04	0.53
IM 5 0h 06.01.10	4.38	0.09	0.24	0.46	0.26	0.49	0.15	0.21	0.5	0.46	3.3	0.71	0.04	0.66	0.46	0.23	0.14	0.46	0.27	1.15	0.05	0.09	0.57
IM 2 6h 11.12.09	8.23	0.21	0.23	1.09	0.19	0.42	0.19	0.17	0.67	0.81	2.68	0.74	0.18	0.96	0.46	0.21	0.2	0.69	0.31	1.94	0.11	0.04	0.97
IM 3 6h 11.12.09	2.77	0.25	0.36	1.19	0.25	0.26	0.32	0.14	0.41	0.67	0.64	0.68	0.36	0.59	0.29	0.54	0.22	0.71	0.22	1.64	0.08	0.07	1.02
IM 4 6h 11.12.09	6.51	0.26	0.51	1.56	0.2	0.29	0.31	0.13	0.63	0.53	0.39	0.69	0.93	0.59	0.28	0.11	0.18	0.63	0.17	1.75	0.08	0.07	0.84
IM 3 6h 01.12.09	0.23	0.37	0.6	2.38	0.3	0.55	0.41	0.23	0.89	0.58	2.76	0.68	0.38	0.81	0.45	0.63	0.44	0.81	0.001	1.94	0.14	0.22	1.06
IM 4 6h 01.12.09	0.29	0.19	0.31	3.25	0.2	0.39	0.3	0.19	0.6	0.92	0.43	0.83	0.66	0.83	0.32	0.34	0.33	0.73	0.55	1.64	0.1	0.53	1.09
IM 1 12h 08.12.09	46.6	1.4	1.87	1.71	1.42	0.86	1.05	0.93	1.18	0.84	1.28	0.85	5.45	0.66	0.96	1.44	1.47	0.84	1.64	1.05	1.06	1.86	0.91
IM 2 12h 08.12.09	51.2	1.1	1.29	1	0.78	0.42	0.13	0.62	0.33	0.68	1.05	0.89	4.59	0.71	0.4	0.55	0.65	0.78	0.13	0.98	0.36	0.85	0.87
IM 1 12h 11.12.09	13.9	0.65	0.5	1.27	0.35	0.26	0.17	0.19	0.43	0.3	0.36	0.63	0.81	0.41	0.24	0.26	0.16	1.04	0.33	1.04	0.12	0.47	0.69
IM 1 12h 14.12.09	7.09	0.57	0.71	0.84	0.54	0.41	0.18	0.37	0.34	0.34	0.63	0.91	2.39	0.54	0.33	0.46	0.41	0.73	0.7	1.09	0.11	0.48	0.9
IM 2 12h 14.12.09	42.8	0.42	0.48	2.89	0.23	0.29	0.07	0.27	0.4	0.58	0.13	0.79	0.83	0.61	0.35	0.21	0.47	1.26	0.64	1.42	0.32	0.31	1.07
IM 1 18h 17.12.09	0.001	1.4	0.96	3.43	11.2	0.65	6.91	0.67	0.3	0.52	1.6	1.27	6.91	0.58	0.36	5.48	19.8	1.37	0.56	0.74	1.68	4.48	1
IM 2 18h 17.12.09	0.001	1.58	1.03	1.56	0.56	0.44	0.28	0.36	0.5	0.32	1.18	0.78	1.29	0.42	0.4	0.71	0.71	1.19	0.33	1.05	0.04	1.76	0.9
IM 3 18h 17.12.09	0.001	0.7	0.52	0.63	0.63	0.34	0.41	0.27	0.3	0.29	2.99	0.78	0.62	0.35	0.22	0.63	0.97	1.04	0.39	0.74	0.08	1.33	0.67
IM 4 18h 26.11.09	0.001	4.55	2.98	6.37	0.8	0.55	0.28	1.03	0.82	0.59	0.54	1.26	11.7	0.45	0.39	0.91	0.3	1.37	0.2	1.57	0.31	2.03	1.3
IM 5 18h 26.11.09	0.001	0.59	0.45	2.57	0.15	0.34	0.08	0.4	0.8	0.33	0.36	0.9	0.33	0.41	0.26	0.43	0.15	0.93	0.48	1.07	0.47	0.18	0.8
IM 1 30h 08.01.10	0.001	0.93	0.61	2	0.46	0.53	0.05	0.92	0.35	0.53	0.88	1.17	1.7	0.6	0.58	0.45	0.4	1.06	0.26	1.23	0.12	0.91	0.95
IM 2 30h 08.01.10	0.001	2.24	1.13	1.44	0.76	0.94	1.96	1.11	0.29	0.5	2.01	1.06	1.03	1.13	0.86	0.8	3.08	0.96	0.2	0.97	1.54	0.84	0.92
IM 3 30h 08.01.10	0.001	1.52	1.04	1.17	0.82	0.53	0.58	0.71	0.28	0.62	0.67	0.96	2.17	0.59	0.47	0.83	0.44	0.83	0.25	0.77	0.37	1.01	0.8
IM 3 30h 15.12.09	0.001	2.34	1.25	2.46	0.95	0.65	0.25	2.24	0.3	1.77	0.39	1.32	2.55	0.87	0.54	0.98	1.05	1.39	0.33	0.65	0.95	1.12	1.43
IM 4 30h 15.12.09	0.001	0.94	0.44	1.38	0.78	0.54	0.26	0.46	0.58	0.39	2.29	1.09	0.47	0.46	0.77	0.34	0.38	0.93	0.23	0.82	0.36	0.28	0.83
IM 1 2d 03.12.09	0.001	0.94	0.59	1.89	0.5	0.66	0.03	1.48	0.41	1.61	1.97	1.16	0.39	1.08	0.8	0.42	0.42	1.31	0.32	0.83	0.16	0.31	1.15
IM 2 2d 03.12.09	0.001	5.22	1.8	2.2	1.36	1	0.5	2.55	0.43	0.94	0.16	1.09	4.64	0.92	1.03	2.35	0.64	1.52	0.65	0.77	0.1	1.88	1.27
IM 3 2d 10.12.09	0.0003	2.05	1.96	0.57	1.07	1.67	3.86	4.56	1.08	1.12	0.71	1.2	1.72	1.6	2.22	1.13	1.21	1.51	2.84	0.9	3.73	2.05	1.18
IM 4 2d 10.12.09	0.001	0.94	0.51	0.75	0.22	0.4	0.27	0.6	0.61	0.42	0.78	0.99	0.46	0.68	0.24	0.29	0.33	0.61	0.07	0.57	0.06	0.22	0.6
IM 5 2d 10.12.09	0.001	0.94	0.45	2.4	0.32	0.65	0.34	0.93	0.49	0.69	0.2	1.3	0.41	1.07	0.81	0.33	0.18	0.96	0.27	1.26	0.07	0.2	1.2
IM 1 5d 21.04.10	0.6	2.2	0.75	0.4	0.91	1.62	0.55	2.56	0.57	1.81	0.02	0.94	0.65	1.58	0.98	1.24	1.5	0.97	0.32	0.52	0.31	1.11	0.96
IM 2 5d 21.04.10	33.4	4.89	3.1	2.2	2.77	3.1	1.1	7.28	1.02	2.16	0.003	1.64	2.86	2.22	2.3	3.11	1.52	2.62	0.96	0.74	3.97	1.86	1.62
IM 3 5d 21.04.10	6111	4.41	7.38	0.84	11.4	8.32	66.7	12.3	12.1	5.16	5.35	1.23	4.97	3.04	11.2	10.6	18.4	1.35	84.7	1.02	125.3	23.9	1.5
IM 4 5d 07.12.09	6301	2.65	5.65	1.09	12.3	8.31	79	11.1	14	5.34	7.15	1.22	5.11	3.42	12.9	10.9	18.2	1.2	106.2	0.74	149.3	24.4	1.55
IM 5 5d 21.04.10	8093	3.36	6.09	1.05	11.2	7.93	92.2	10.5	16.9	6.57	21.1	1.37	4.72	3.19	15.2	10.8	24.7	1.45	115.8	1.33	193.3	31.9	1.72
IM 4 5d 21.04.10	n.m.	n.m.	n.m.	n.m.	n.m.	n.m.	n.m.	n.m.	n.m.	n.m.	n.m.	n.m.	n.m.	n.m.	n.m.	n.m.	n.m.	n.m.	n.m.	n.m.	n.m.	n.m.	n.m.
IM 1 14d 09.12.09	12244	5.1	8.58	1.32	17.8	11	123.8	15.3	23.5	7.17	22	1.46	6.61	4.24	18.5	30.5	29.9	1.34	170	0.85	233.1	42	1.6
IM 2 14d 09.12.09	14869	6.05	11	1.62	25.1	15	165.8	20.2	29.8	7.75	14.9	1.57	8.31	4.62	25.8	22.1	38.8	1.57	207.5	0.75	312.3	47	1.58
IM 3 14d 09.12.09	4975	3.22	4.43	0.83	9.04	6.07	54.8	8.6	11.2	4.39	11.1	1.07	3.29	2.49	10.4	8.14	14.1	1.02	69.3	0.69	92	16.4	1.16
IM 1 14d 16.12.09	9207	6	8.41	0.9	17	13.4	84.3	16.6	15.6	6.24	11.6	1.49	4.8	3.72	19.6	16	23.2	1.69	117.2	0.94	182	26.8	1.63
IM 2 14d 16.12.09	3243	6.16	4.66	1.12	11.9	9.82	46.7	15.9	7.42	3.59	8.49	1.26	3.28	3.13	14.1	10.4	13.2	1.77	54	0.72	73.3	13.5	1.16
