# Supplementary material for: Pathobiochemical signatures of cholestatic liver disease in bile duct ligated mice
Source: BMC Syst Biol. 2015 Nov 20;9:83. doi: 10.1186/s12918-015-0229-0 (PMC4654904; doi:10.1186/s12918-015-0229-0)
Supplement: Additional file 2: — Dataset S2. Statistical analysis. (PDF 943 kb) [file 12918_2015_229_MOESM2_ESM.pdf]

Statistical Analysis of Pathobiochemical Signatures in Bile Duct Ligated Mice

Matthias Koenig (2015-10-22)

Contents

Introduction **2**

Explorative data analysis **3**

Data import 3
Gene Probes 4
Data visualization 7
Actb quality control 11

Dimension reduction **12**

ANOVA for single factor 13
ANOVA for all factors 14
Filter factors 17
t-test for initial phase 20

Correlation analysis **21**

Pearson & Spearman correlation 22
YS & YR correlation 25

Hierarchical clustering **27**

Clustering 27
Correlations for non-transcript factors 31
Mean cluster time course 36
Top cluster representatives 39
YS3 cluster summary 42

Decision trees **45**

Trainings data 46
Fit regression tree 48
Leave-one-out cross validation 51
Prediction on trainings data 52
Test data for evaluation 54
Prediction on test data 56

Best factor combinations 57
Prediction errors 61
Predictive performance 62

Introduction

This document contains the statistical analysis performed in the publication *Pathobiochemical signatures of cholestatic liver disease in bile duct ligated mice.*

A comprehensive data set of serum markers, histological parameters and transcript profiles was compiled at 8 time points after bile duct ligation (BDL) in mice, comprising different stages of the disease. The data set consists of $N_r = 5$ repeats ($N_r = 3$ for CTGF, α-SMA and S100a4) for $N_t = 8$ time points denoted by $t_1, ..., t_{N_t}$ consisting of a total $N_f = 153$ measured parameters in the following referred to as factors (Fluidigm gene expression, biochemical markers, and (immuno-)histochemical measurements).

The following naming conventions are used

- **factor** : one of the measured quantities/parameters over time, i.e. either
  - gene expression of a single gene (e.g. Actb);
  - one of the bichemical markers (e.g. ALT, albumin, bilirubin)
  - one of the (immuno-)histochemical markers (e.g. BrdU-positive Kupffer cells, CTGF)
- **time point** : a single value t_i from the measured time points 0h (control), 6h, 12h, 18h, 30h, 2d, 5d and 14d
- **sample** : one of the $N_t N_r = 40$ mice, i.e. a one of the repeats for a given time point

The main steps of the analysis comprise

- **Explorative data anaysis**
- **Dimension reduction via ANOVA**
- **Correlation analysis**
- **Hierarchical clustering**
- **Decision trees**

The HTML version of this document is available from
http://matthiaskoenig.github.io/bdl-analysis/
All data sets, source code and documentation are provided at
https://github.com/matthiaskoenig/bdl-analysis

All results of this analysis are written to the results directory defined via the `BDL_RESULTS` environment variable. To reproduce this analysis create the respective variable and run the `Koenig_BDL_analysis.Rmd` file.

```
# read results directory from environment variable
resultsPath <- Sys.getenv("BDL_RESULTS")
if (identical(resultsPath, "")){
  stop("No results directory defined, set the BDL_RESULTS environment variable")
}
# create directory to store data sets of analysis
dir.create(file.path(resultsPath, 'data'), showWarnings=FALSE)
```

Explorative data analysis

Data import

In a first step the processed data sets are loaded from the `data` folder: These are the time course data for all factors (`BDLdata`), additional information for the factors (`BDLfactors`), the sample definition, i.e. the assignment of sample ids to respective time point and repeat (`BDLsamples`), and a mapping of the Fluidigm (gene) probe ids to UniProt identifiers and names (`BDLprobes`).

```
suppressPackageStartupMessages(library(BDLanalysis))
suppressPackageStartupMessages(library(calibrate))
suppressPackageStartupMessages(library(pander))
dir.create(file.path(resultsPath, 'control'), showWarnings=FALSE)
# path definition
baseLoc <- system.file(package="BDLanalysis")
extPath <- file.path(baseLoc, "extdata")
# load data
data(BDLdata)
data(BDLsamples)
data(BDLfactors)
data(BDLprobes)
# counters
Nr <- 5  # repeats
Nt <- length(levels(BDLsamples$time_fac))  # Nt=8 time points
# store all data sets in the results folder
save(BDLdata, file=file.path(resultsPath, "data", "BDLdata.Rdata"))
save(BDLsamples, file=file.path(resultsPath, "data", "BDLsamples.Rdata"))
save(BDLfactors, file=file.path(resultsPath, "data", "BDLfactors.Rdata"))
save(BDLprobes, file=file.path(resultsPath, "data", "BDLprobes.Rdata"))
```

In addition to the individual sample data, the mean data averaged over the N_r repeats per time points is used in parts of the analysis. The mean factor data set is calculated once via

```
BDLmean <- bdl_mean_data(BDLdata, BDLsamples)
BDLmean.time <- as.numeric(levels(as.factor(BDLsamples$time)))
```

In total 153 factors were measured in the this BDL study falling in the categories: Biochemistry, GE_ADME, GE_Cytokines, GE_Fibrosis, Histochemistry. The majority of factors belongs hereby to the 3 fluidigm chips with 47 probes per chip (one probe was filtered from GE fibrosis during preprocessing of the chips).

An overview of the number of factors per category is provided in the following table

```
cat_table <- as.data.frame(table(BDLfactors$ftype))
colnames(cat_table) <- c("Category", "Freq")
set.caption(sub(".", " ", "Factors per category", fixed = TRUE))
pander(cat_table)
```

Table 1: Factors per category

Category	Freq
Biochemistry	4
GE_ADME	47

Category	Freq
GE_Cytokines	47
GE_Fibrosis	46
Histochemistry	9

```
rm(cat_table)
```

Gene Probes

In the following an overview of the gene probes is given providing full names and links to UniProt.

```
# create data frame with probe information
create_probe_df <- function() {
  # get the gene factors
  f_names <- colnames(BDLdata)[BDLfactors$ftype.short == ""]
  # get the probe information
  Nf = length(f_names)
  probe_names <- rep(NA, length=Nf)
  probe_uniprot <- rep(NA, length=Nf)
  probe_genes <- rep(NA, length=Nf)
  names(probe_names) <- f_names
  names(probe_uniprot) <- f_names
  names(probe_genes) <- f_names
  for (name in colnames(BDLdata)){
      probe_info <- ProbeInformation(name)
      if (!is.null(probe_info)){
        if (!is.null(probe_info$Protein.name)){
          probe_names[name] <- probe_info$Protein.name
          probe_uniprot[name] <- probe_info$Entry
          probe_genes[name] <- probe_info$Gene.name
        }
      }
  }
  probe.df <- data.frame(name=probe_names, genes=probe_genes, uniprot=probe_uniprot)
  # sort
  probe.df <- probe.df[order(rownames(probe.df)), ]
  # remove the double Act probes
  probe.df <- probe.df[!(rownames(probe.df) %in% c("Actb.x", "Actb.y")) , ]
  return(probe.df)
}
# print probe information
probe.df <- create_probe_df()
```

```
options(width=200)
print(probe.df)
```

```
                                          name                                      genes uniprot
Abcb1a          Multidrug resistance protein 1A  Abcb1a, Abcb4, Mdr1a, Mdr3, Pgy-3, Pgy3  P21447
Abcc2    Canalicular multispecific organic a...                                     Abcc2  Q8VI47
Abcg2    ATP-binding cassette sub-family G m...                       Abcg2, Abcp, Bcrp1  Q7TMS5
Acta2                Actin, aortic smooth muscle                      Acta2, Actsa, Actvs  P62737
```

Actb	Actin, cytoplasmic 1	Actb	P60710
Ahr	Aryl hydrocarbon receptor	Ahr	P30561
Bad	Bcl2-associated agonist of cell dea...	Bad, Bbc6	Q61337
Bak1	Bcl-2 homologous antagonist/killer	Bak1, Bak	O08734
Bax	Apoptosis regulator BAX	Bax	Q07813
Bcl2l11	Bcl-2-like protein 11	Bcl2l11, Bim	O54918
Birc5	Baculoviral IAP repeat-containing p...	Birc5, Api4, Iap4	O70201
Ccl2	C-C motif chemokine 2	Ccl2, Je, Mcp1, Scya2	P10148
Ccl3	C-C motif chemokine 3	Ccl3, Mip1a, Scya3	P10855
Ccl4	C-C motif chemokine 4	Ccl4, Mip1b, Scya4	P14097
Ccl5	C-C motif chemokine 5	Ccl5, Scya5	P30882
Ccl7	C-C motif chemokine 7	Ccl7, Fic, Mcp3, Scya7	Q03366
Ccl8	C-C motif chemokine 8	Ccl8, Mcp2, Scya8	Q9Z121
Ccr2	C-C chemokine receptor type 2	Ccr2, Cmkbr2	P51683
Ccr3	Probable C-C chemokine receptor typ...	Ccr3, Cmkbr1l2, Cmkbr3	P51678
Ccr5	C-C chemokine receptor type 5	Ccr5, Cmkbr5	P51682
Cd14	Monocyte differentiation antigen CD...	Cd14	P10810
Cd69	Early activation antigen CD69	Cd69	P37217
Cd86	T-lymphocyte activation antigen CD8...	Cd86	P42082
Cdh1	Cadherin-1	Cdh1	P09803
Cdh2	Cadherin-2	Cdh2	P15116
Cebpa	CCAAT/enhancer-binding protein alph...	Cebpa, Cebp	P53566
Cebpb	CCAAT/enhancer-binding protein beta	Cebpb	P28033
Cebpd	CCAAT/enhancer-binding protein delt...	Cebpd, Crp3	Q00322
Ch25h	Cholesterol 25-hydroxylase	Ch25h	Q9Z0F5
Col1a1	Collagen alpha-1(I) chain	Col1a1, Cola1	P11087
Col3a1	Collagen alpha-1(III) chain	Col3a1	P08121
Col4a3	Collagen alpha-3(IV) chain	Col4a3	Q9QZS0
Col6a6	Collagen alpha-6(VI) chain	Col6a6	Q8C6K9
Col8a1	Collagen alpha-1(VIII) chain	Col8a1	Q00780
Ctgf	Connective tissue growth factor	Ctgf, Ccn2, Fisp-12, Fisp12, Hcs24	P29268
Cxcl1	Growth-regulated alpha protein	Cxcl1, Gro, Gro1, Mgsa, Scyb1	P12850
Cxcl15	C-X-C motif chemokine 15	Cxcl15, Scyb15	Q9WVL7
Cxcl2	C-X-C motif chemokine 2	Cxcl2, Mip-2, Mip2, Scyb2	P10889
Cxcl3	C-X-C motif chemokine 3	Cxcl3, Dcip1, Gm1960	Q6W5C0
Cxcl5	C-X-C motif chemokine 5	Cxcl5, Scyb5	P50228
Cxcr1	C-X-C chemokine receptor type 1	Cxcr1, Il8ra	Q810W6
Cxcr2	C-X-C chemokine receptor type 2	Cxcr2, Cmkar2, Gpcr16, Il8rb	P35343
Cyp1a2	Cytochrome P450 1A2	Cyp1a2, Cyp1a-2	P00186
Cyp24a1	1,25-dihydroxyvitamin D(3) 24-hydro...	Cyp24a1, Cyp-24, Cyp24	Q64441
Cyp2b10	Cytochrome P450 2B10	Cyp2b10, Cyp2b-10, Cyp2b20	P12791
Cyp2c29	Cytochrome P450 2C29	Cyp2c29	Q64458
Cyp2c37	Cytochrome P450 2C37	Cyp2c37	P56654
Cyp2c39	Cytochrome P450 2C39	Cyp2c39	P56656
Cyp2d22			
Cyp2e1	Cytochrome P450 2E1	Cyp2e1, Cyp2e, Cyp2e-1	Q05421
Cyp3a11	Cytochrome P450 3A11	Cyp3a11, Cyp3a-11	Q64459
Cyp4a10	Cytochrome P450 4A10	Cyp4a10, Cyp4a-10	O88833
Cyp7a1	Cholesterol 7-alpha-monooxygenase	Cyp7a1, Cyp7	Q64505
Dpyd	Dihydropyrimidine dehydrogenase [NA...	Dpyd	Q8CHR6
Edn1	Endothelin-1	Edn1	P22387
Egf	Pro-epidermal growth factor	Egf	P01132
Egfr	Epidermal growth factor receptor	Egfr	Q01279
Fasl	Tumor necrosis factor ligand superf...	Faslg, Apt1lg1, Cd95l, Fasl, gld, Tnfsf6	P41047
Fn1	Fibronectin	Fn1	P11276
Gdf2	Growth/differentiation factor 2	Gdf2, Bmp9	Q9WV56
Gsta2	Glutathione S-transferase A2	Gsta2	P10648
Gstm1	Glutathione S-transferase Mu 1	Gstm1	P10649
Gstp1	Glutathione S-transferase P 1	Gstp1, Gstpib	P19157

Hgf	Hepatocyte growth factor receptor	Met	P16056
Hk2	Hexokinase-2	Hk2	O08528
Hmox1	Heme oxygenase 1	Hmox1	P14901
Hnf4a	Hepatocyte nuclear factor 4-alpha	Hnf4a, Hnf-4, Hnf4, Nr2a1, Tcf14	P49698
Ifna1	Interferon alpha-1	Ifna1	P01572
Ifnar1	Interferon alpha/beta receptor 1	Ifnar1, Ifar, Ifnar	P33896
Ifnar2	Interferon alpha/beta receptor 2	Ifnar2	O35664
Ifnb1	Interferon beta	Ifnb1, Ifb, Ifnb	P01575
Ifng	Interferon gamma	Ifng	P01580
Igf1	Insulin-like growth factor I	Igf1, Igf-1	P05017
Il10	Interleukin-10	Il10, Il-10	P18893
Il10ra	Interleukin-10 receptor subunit alp...	Il10ra, Il10r	Q61727
Il10rb	Interleukin-10 receptor subunit bet...	Il10rb, Crfb4	Q61190
Il13	Interleukin-13	Il13, Il-13	P20109
Il17a	Interleukin-17A	Il17a, Ctla8, Il17	Q62386
Il1b	Interleukin-1 beta	Il1b	P10749
Il1rn	Interleukin-1 receptor antagonist p...	Il1rn, Il-1ra	P25085
Il2	Interleukin-2	Il2, Il-2	P04351
Il28b	Interferon lambda-3	Ifnl3, Il28, Il28b	Q8CGK6
Il4	Interleukin-4	Il4, Il-4	P07750
Il6	Interleukin-6	Il6, Il-6	P08505
Il6st	Interleukin-6 receptor subunit beta	Il6st	Q00560
Lama1	Laminin subunit alpha-1	Lama1, Lama, Lama-1	P19137
Met	Hepatocyte growth factor receptor	Met	P16056
Mki67	MKI67 FHA domain-interacting nucleo...	Nifk, Mki67ip	Q91VE6
Mrc1	Macrophage mannose receptor 1	Mrc1	Q61830
Nes	Nestin	Nes	Q6P5H2
Nfkbia	NF-kappa-B inhibitor alpha	Nfkbia, Ikba	Q9Z1E3
Nos2	Nitric oxide synthase, inducible	Nos2, Inosl	P29477
Notch1	Neurogenic locus notch homolog prot...	Notch1, Motch	Q01705
Notch3	Neurogenic locus notch homolog prot...	Notch3	Q61982
Nr0b2	Nuclear receptor subfamily 0 group ...	Nr0b2, Shp	Q62227
Nr1h3	Oxysterols receptor LXR-alpha	Nr1h3, Lxra	Q9Z0Y9
Nr1i2	Nuclear receptor subfamily 1 group ...	Nr1i2, Pxr	O54915
Nr1i3	Nuclear receptor subfamily 1 group ...	Nr1i3, Car	O35627
Nr2f1	COUP transcription factor 1	Nr2f1, Erbal3, Tfcoup1	Q60632
Nr2f2	COUP transcription factor 2	Nr2f2, Aporp1, Arp-1, Arp1, Tfcoup2	P43135
Nr3c1	Glucocorticoid receptor	Nr3c1, Grl, Grl1	P06537
Osm	Oncostatin-M	Osm	P53347
Osmr	Oncostatin-M-specific receptor subu...	Osmr, Osmrb	O70458
Pde4a	cAMP-specific 3',5'-cyclic phosphod...	Pde4a	O89084
Pde4b	cAMP-specific 3',5'-cyclic phosphod...	Pde4a	O89084
Pde4d	cAMP-specific 3',5'-cyclic phosphod...	Pde4d	Q01063
Pdgfb	Platelet-derived growth factor subu...	Pdgfb, Sis	P31240
Por	NADPH--cytochrome P450 reductase	Por	P37040
Ppara	Peroxisome proliferator-activated r...	Ppara, Nr1c1, Ppar	P23204
Pparg	Peroxisome proliferator-activated r...	Pparg, Nr1c3	P37238
Prom1	Prominin-1	Prom1, Prom, Proml1	O54990
Pten	Phosphatidylinositol 3,4,5-trisphos...	Pten, Mmac1	O08586
Ptgs2	Prostaglandin G/H synthase 2	Ptgs2, Cox-2, Cox2, Pghs-b, Tis10	Q05769
Rarres1	Cytosolic carboxypeptidase 2	Agbl2, Ccp2	Q8CDK2
Rps18	40S ribosomal protein S18	Rps18	P62270
Rxra	Retinoic acid receptor RXR-alpha	Rxra, Nr2b1	P28700
Slc10a1	Sodium/bile acid cotransporter	Slc10a1, Ntcp	O08705
Smad6	Mothers against decapentaplegic hom...	Smad6, Madh6, Madh7, Msmad6	O35182
Smad7	Mothers against decapentaplegic hom...	Smad7, Madh7, Madh8	O35253
Socs1	Suppressor of cytokine signaling 1	Socs1, Cish1, Ssi1	O35716
Socs3	Suppressor of cytokine signaling 3	Socs3, Cis3, Cish3	O35718
Sod2	Superoxide dismutase [Mn], mitochon...	Sod2, Sod-2	P09671

```
Sparc                                     SPARC                                 Sparc  P07214
Sult1a1                       Sulfotransferase 1A1          Sult1a1, St1a1, Stp, Stp1  P52840
Sult1b1  Sulfotransferase family cytosolic 1...                               Sult1b1  Q9QWG7
Tgfb1         Transforming growth factor beta-1                                 Tgfb1  P04202
Tgfb2         Transforming growth factor beta-2                                 Tgfb2  P27090
Tgfbr2                 TGF-beta receptor type-2                                Tgfbr2  Q62312
Timp1             Metalloproteinase inhibitor 1                  Timp1, Timp, Timp-1  P12032
Timp2             Metalloproteinase inhibitor 2                        Timp2, Timp-2  P25785
Tnc                                    Tenascin                             Tnc, Hxb  Q80YX1
Tnf                       Tumor necrosis factor                   Tnf, Tnfa, Tnfsf2  P06804
Tnfrsf1a Tumor necrosis factor receptor supe...             Tnfrsf1a, Tnfr-1, Tnfr1  P25118
Tnfrsf1b Tumor necrosis factor receptor supe...             Tnfrsf1b, Tnfr-2, Tnfr2  P25119
Ugt1a1          UDP-glucuronosyltransferase 1-1                        Ugt1a1, Ugt1  Q63886
Vdr                          Vitamin D3 receptor                          Vdr, Nr1i1  P48281
Wisp1    WNT1-inducible-signaling pathway pr...                  Wisp1, Ccn4, Elm1  O54775
Xiap           E3 ubiquitin-protein ligase XIAP      Xiap, Aipa, Api3, Birc4, Miha  Q60989
```

```
options(width=75)
```

Data visualization

Single chip data

Create heatmap of the mean chip data

```
suppressPackageStartupMessages(library(gplots))
suppressPackageStartupMessages(library(RColorBrewer))

data_ADME = t(as.matrix(BDLmean[BDLfactors$ftype == "GE_ADME"]))
data_ADME <- data_ADME[rownames(data_ADME) != "Actb.x", ]

data_Cytokines = t(as.matrix(BDLmean[BDLfactors$ftype == "GE_Cytokines"]))
data_Cytokines <- data_Cytokines[rownames(data_Cytokines) != "Actb.y", ]

data_Fibrosis = t(as.matrix(BDLmean[BDLfactors$ftype == "GE_Fibrosis"]))
data_Fibrosis <- data_Fibrosis[rownames(data_Fibrosis) != "Actb", ]

# fold changes relative to sham mice (0 h) displayed in log2 scale
log2_to_control <- function(data){
  data <- data[sort(rownames(data)), ]  # sort by name
  reldata <- data[, 2:ncol(data)]/data[, 1]
  log2_reldata <- log2(reldata)
}

data_list <- list(ADME=data_ADME,
                  Fibrosis=data_Fibrosis,
                  Cytokines=data_Cytokines)
for (name in names(data_list)){
  data <- data_list[[name]]
  cor_colors <- HeatmapColors() # color palette for correlation (red - white - blue)
  hmap_colors <- cor_colors(100)
  # plot to file
  pdf(file.path(resultsPath, "control", paste("chip_heatmap_", name, ".pdf", sep="")),
      width=8, height=10, pointsize=12)
```

```
  heatmap.2(log2_to_control(data), col=hmap_colors, breaks=seq(from=-2, to=2, length.out=length(hmap_col
            scale="none",
            key=TRUE, symkey=FALSE, key.xlab = "log2",
            trace="none", cexRow=0.8, cexCol=0.8,
            symm=FALSE,
            density.info="none", dendrogram="none",
            Rowv=NULL, Colv=NULL,
            keysize=0.8,
            sepwidth=c(0.01,0.01),
            sepcolor="white")
  invisible(dev.off())
}
```

Time course of single factors

To get an overview over the BDL data set plots of the raw and mean data for all individual factors over time were generated. These are available in the `resultsPath/factors` folder under the respective names of the factors

```
# Create figures for all factors
factors_path <- file.path(resultsPath, 'factors')
dir.create(factors_path, showWarnings=FALSE)
plot_all_factors(path=factors_path)
rm(factors_path)
```

An example plot for a single factor is depicted for `bilirubin`. A continous increase in bilirubin can be observed after BDL.

```
plot_single_factor('bilirubin', path=NULL)
```

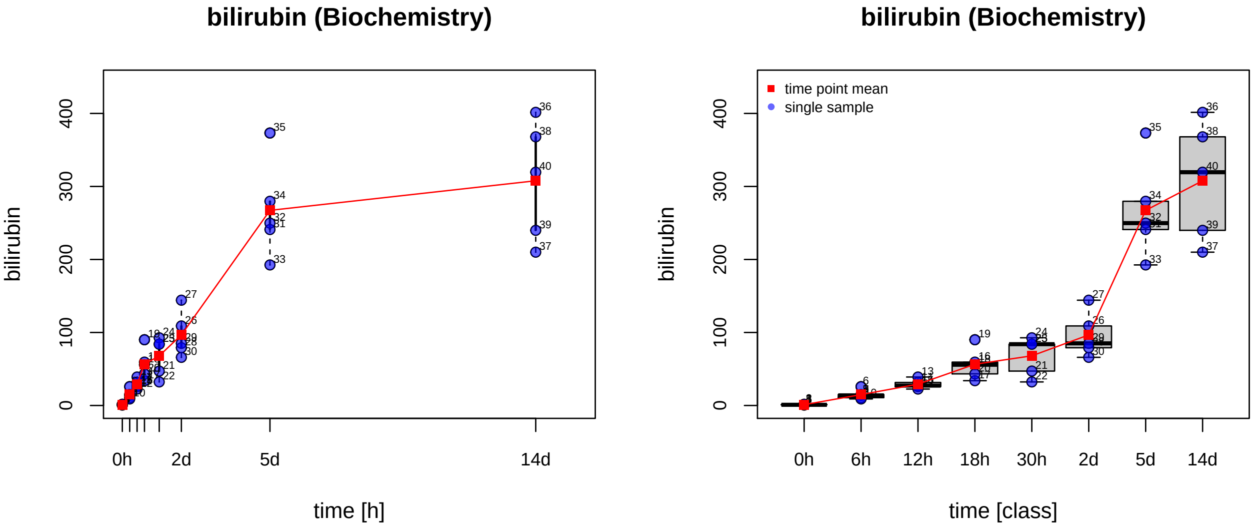

Figure: Bilrubin timecourse. Plot of the raw time course data for bilirubin. On the left the data is plotted against the time [h], on the right against the different time classes. Individual data points are depecticed in blue with the respective sample number shown next to the data points. The mean averaged of the repeats per time point are depicted in red.

Time course of all factors

Next the heatmap overview of the complete data set is generated, i.e. the data of all factors for of all time points and repeats. This provides a first overview over the complete data set.

```
# define horizontal and vertical helper lines
v_lines <- ((1:Nt)*Nr+0.5)
factor_types <- c("Histochemistry", "Biochemistry",
                  "GE_Fibrosis", "GE_Cytokines", "GE_ADME")
factor_table <- table(BDLfactors$ftype)
h_lines <- 0.5 + cumsum(factor_table[factor_types])

timecourse_heatmap <- function(){
  # create better row names
  dtmp <- BDLdata
  rownames(dtmp) <- paste(rownames(BDLsamples), BDLsamples$time_fac, sep=" ")
  # heatmap colors
  hmap_colors <- HeatmapColors()
  # colors for factor groups
  colorset <- brewer.pal(length(factor_types), "Set2")
  color.map <- function(factor_id) {
    return(
      colorset[which(factor_types==BDLfactors$ftype[which(BDLfactors$id==factor_id)])]
    )
  }
  factorColors <- unlist(lapply(BDLfactors$id, color.map))
  # heatmap
  heatmap.2(t(as.matrix(dtmp)), col=hmap_colors(100), scale="row",
            dendrogram="none", Rowv=NULL, Colv=NULL,
            key=TRUE, trace="none", cexRow=0.5, keysize=0.8, density.info="none",
            RowSideColors=factorColors,
            add.expr=abline(v=v_lines, h=h_lines, col="black", lwd=0.5),
            main="Heatmap of BDL time course data")
            # xlab="sample", ylab="factor")
  # legend
  legend("left",
      inset=c(-0.03,0),
      legend = rev(factor_types), # category labels
      col = rev(colorset),  # color key
      lty= 1, lwd = 10, cex = 0.7, bty="n"
  )
}
# plot to file
pdf(file.path(resultsPath, "control", "timecourse_heatmap.pdf"),
    width=10, height=10, pointsize=12)
timecourse_heatmap()
invisible(dev.off())
```

```
timecourse_heatmap()
```

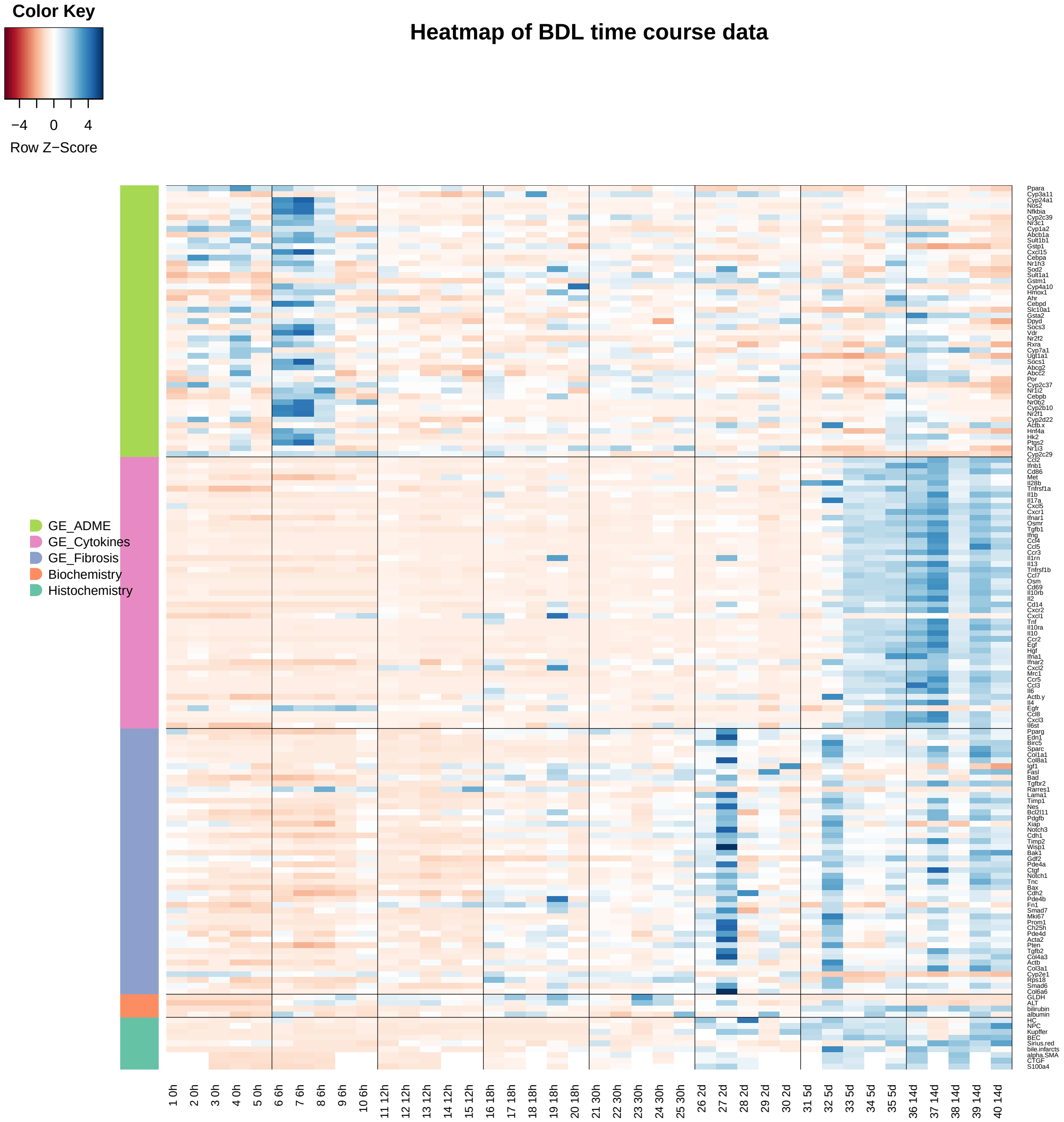

Figure: Timecourse heatmap of all factors. Rows correspond to individual factors, with factor order corresponding to the original data set: `GE_ADME`, `GE_Cytokines`, `GE_Fibrosis`, `Biochemistry`, `Histochemistry`. Columns correspond to the 40 samples with 5 subsequent samples belonging to one of the 8 time points. The data is row scaled, i.e. every individual factor is scaled to have mean zero and standard deviation one.

Results: Various patterns are visible in the plotted raw data set:

- **Two main classes of time course response are observed**. One class with an increase in the early phase up to 6h after BDL (many of the ADME genes fall in this class) and a second class increasing in the later stage after 2-5 days after BDL. Many of the genes on the Cytokines and Fibrosis chips as well as some of the biochemical and (immuno-)histochemical factors fall in this second class.
- **The individual animals show heterogeneous responses to BDL**. Within one time point the 5 repeats can show very different patterns. For instance at time 6h after BDL 3/5 of the mice show

a marked increase in the ADME genes, whereas 2/5 do not show such a marked increase. Another example is the mice sample 27 at time 2d, with a high increase in the genes on the Fibrosis chip, which is not observed in the other 4 samples at time 2d.

Actb quality control

Actb (Actin, cytoplasmic 1) probes were included on all Fluidigm chips (`GE_ADME`, `GE_Cytokines`, `GE_Fibrosis`) and not used in the normalization of the transcription data. Hence, ActB can serve as quality control for the technical reproducibility of the Fluidigm chips. If the data is reproducible between chips the pairwise correlation between all individual Actb measurements should have high correlation coefficients close to 1. Plotting the data of the Actb measurements of two chips against each other should lie on a straight line.

```
# Actb control figure
plot_actb_control <- function(){
  par(mfrow=c(2,3))
  plot_single("Actb")
  plot_single("Actb.x")
  plot_single("Actb.y")
  plot_cor_pair("Actb", "Actb.x", single_plots=FALSE)
  plot_cor_pair("Actb", "Actb.y", single_plots=FALSE)
  plot_cor_pair("Actb.x", "Actb.y", single_plots=FALSE)
  par(mfrow=c(1,1))
}

# plot to file
pdf(file.path(resultsPath, "control", "Actb_control.pdf"),
    width=10, height=6, pointsize=12)
plot_actb_control()
invisible(dev.off())

# calculate Spearman and Pearson correlation coefficients on N=8*5=40 data points
actb.spearman <- cor(data.frame(Actb=BDLdata$Actb,
                                Actb.x=BDLdata$Actb.x,
                                Actb.y=BDLdata$Actb.y), method="spearman")
actb.pearson <- cor(data.frame(Actb=BDLdata$Actb,
                               Actb.x=BDLdata$Actb.x,
                               Actb.y=BDLdata$Actb.y), method="pearson")

# table of correlation coefficients
set.caption(sub(".", " ", "Spearman correlation of Actb controls", fixed = TRUE))
pander(round(actb.spearman, digits=3))
```

Table 2: Spearman correlation of Actb controls

	Actb	Actb.x	Actb.y
**Actb**	1	0.908	0.944
**Actb.x**	0.908	1	0.917
**Actb.y**	0.944	0.917	1

```
set.caption(sub(".", " ", "Pearson correlation of Actb controls", fixed = TRUE))
pander(round(actb.pearson, digits=3))
```

Table 3: Pearson correlation of Actb controls

	Actb	Actb.x	Actb.y
**Actb**	1	0.945	0.938
**Actb.x**	0.945	1	0.935
**Actb.y**	0.938	0.935	1

```
plot_actb_control()
```

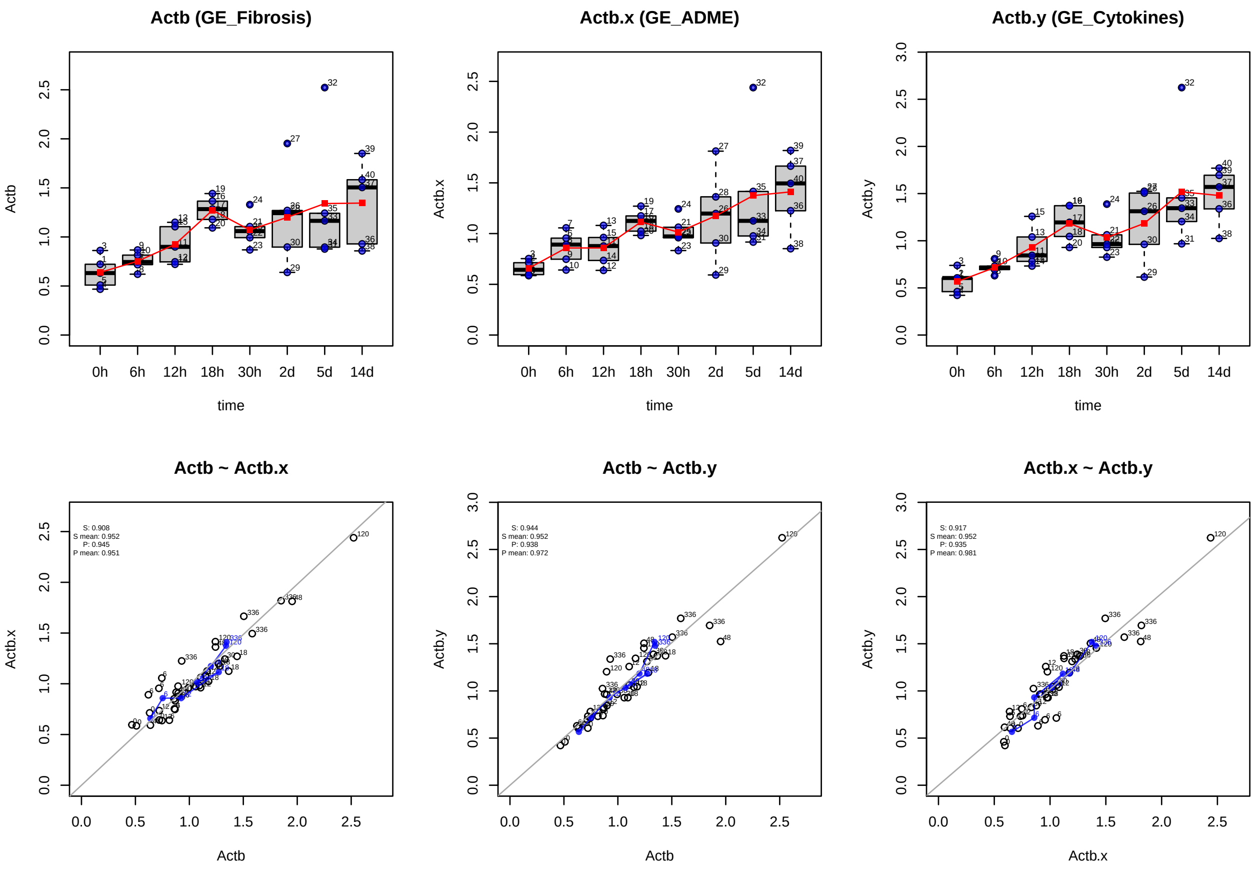

Figure: Actb control. Correlation plot of the Actb probes from the 3 Fluidigm chips: Actb (fibrosis), Actb.x (ADME), Actb.y (Cytokines). The top row shows the individual time courses, the bottom row the pair wise plot of individual data points.

Results: The Actb Fluidigm gene expression measurements are highly reproducible for the measured chips, with Spearman as well as Pearson correlation coefficients all > 0.9 for pairwise Actb comparison.

Dimension reduction

A one-way analysis of variance (ANOVA) was applied to reduce the factors to the subset showing significant ($p_{adj} < 0.05$) changes during the time course. In its simplest form, ANOVA provides a statistical test of whether or not the means of several groups are equal, and therefore generalizes the t-test to more than two groups, with the groups being the sampled time points. The Holm's procedure was used to correct the p-values for any artificial p-value inflation due to multiple testing.

In a first step, an ANOVA was calculated with the groups corresponding to the different time points for every factor in the BDL data set. Dimension reduction was than performed by filtering out those factors which did not significantly change over time.

The `BDLdata` data set is reshaped into matrix format for the ANOVA calculation, with time points in rows and repeats as columns for every factor.

```
BDLmatrices <- bdl_matrix_data(BDLdata, BDLsamples)
```

ANOVA for single factor

The following shows the ANOVA calculation for a single factor. We use again the example `bilirubin` to demonstrate the method.

```
# example ANOVA for one factor
mat.anova <- t(BDLmatrices[['bilirubin']])
colnames(mat.anova) <- levels(BDLsamples$time_fac)
print(mat.anova)
```

```
     0h    6h   12h   18h   30h     2d    5d   14d
R1 1.49 25.80 31.51 59.40 47.11 108.95 241.0 401.5
R2 0.93 13.87 22.51 33.95 32.26 144.20 249.8 210.0
R3 1.65 10.70 38.99 55.80 83.98  79.10 192.5 368.0
R4 0.85 15.08 25.81 90.05 92.72  85.10 279.7 239.9
R5 0.53  8.96 27.21 43.30 84.02  65.95 373.2 319.5
```

```
# concatenate the data rows of df1 into a single vector r .
r = c(t(as.matrix(mat.anova)))  # response data

# assign new variables for the treatment levels and number of observations.
f = levels(BDLsamples$time_fac)   # treatment levels
k = Nt                             # number of treatment levels (time points Nt=8)
n = Nr                             # observations per treatment (repeats Nr=5)

# vector of treatment factors that corresponds to each element of r
# in step 3 with the gl function.
tm <- gl(k, 1, n*k, factor(f))   # matching treatments

# apply the function aov to a formula that describes the response r
# by the treatment factor tm (fit ANOVA model)
av <- aov(r ~ tm)

# print ANOVA summary
summary(av)
```

```
            Df Sum Sq Mean Sq F value   Pr(>F)    
tm           7 479470   68496      41 3.27e-14 ***
Residuals   32  53463    1671                     
---
Signif. codes:  0 '***' 0.001 '**' 0.01 '*' 0.05 '.' 0.1 ' ' 1
```

```
# print p-value
p.value <- summary(av)[[1]][["Pr(>F)"]][[1]]
```

Results: The time course fo bilirubin is highly altered after BDL, resulting in a high significance of the ANOVA. This confirms what we saw in the initial visual inspection of bilirubin (see Figure above).

ANOVA for all factors

The ANOVA calculation for all individual factors is performed analog to the calculate for bilirubin. As a consequence of the large number of factors, a multitude of tests are performed, namely an ANOVA for every single factor. Consequently, the reported p-values of the ANOVA have to be adjusted for multiple testing. This is done using the `p.adjust` function which given a set of p-values, returns p-values adjusted using one of several methods. We selected the Holm's method designed to give strong control of the family-wise error rate (*Holm, S. (1979). A simple sequentially rejective multiple test procedure. Scandinavian Journal of Statistics 6, 65-70.*).

```
# Calculation of ANOVA for all factors
df.anova <- all_factor_anova()
df.anova$sig <- sapply(df.anova$p.value, significant_code)  # add significant codes

df.anova$p.holm <- p.adjust(df.anova$p.value, method ="holm",
                            n = length(df.anova$p.value))
df.anova$sig.holm <- sapply(df.anova$p.holm, significant_code)
df.anova$ftype <- BDLfactors$ftype
df.anova$fshort <- BDLfactors$ftype.short

# order factors by adjusted p-values, and cleanup for printing
df.anova.ordered <- df.anova[with(df.anova, order(p.holm)), ]
rownames(df.anova.ordered) <- df.anova.ordered$factors
df.anova.ordered <- df.anova.ordered[c("p.holm", "sig.holm", "ftype", "fshort")]
df.anova.ordered$p.holm <- as.numeric(sprintf("%1.2E", df.anova.ordered$p.holm))

# save results
write.table(df.anova.ordered, file=file.path(resultsPath, "data", 'BDLanova.csv'),
            sep="\t", quote=FALSE)
save(df.anova, file=file.path(resultsPath, "data", "BDLanova.Rdata"))
```

```
print(df.anova.ordered)
```

```
                p.holm sig.holm         ftype fshort
Cyp1a2        2.93e-14      ***       GE_ADME
bilirubin     4.98e-12      ***  Biochemistry      B
Il10rb        1.15e-11      ***  GE_Cytokines
Tgfb1         3.30e-11      ***  GE_Cytokines
Ccl2          3.46e-11      ***  GE_Cytokines
Cd86          6.56e-11      ***  GE_Cytokines
Ccr2          6.94e-11      ***  GE_Cytokines
Mrc1          6.95e-11      ***  GE_Cytokines
Tnfrsf1b      7.89e-11      ***  GE_Cytokines
Cxcl5         6.26e-10      ***  GE_Cytokines
CTGF          7.82e-10      *** Histochemistry      H
Il10ra        1.64e-09      ***  GE_Cytokines
Gstm1         9.18e-09      ***       GE_ADME
```

Ccl7	3.40e-08	***	GE_Cytokines	
Ccr5	4.36e-08	***	GE_Cytokines	
Hgf	5.80e-08	***	GE_Cytokines	
Osmr	1.01e-07	***	GE_Cytokines	
Ccl4	1.04e-07	***	GE_Cytokines	
Nr0b2	1.30e-07	***	GE_ADME	
Tgfbr2	1.71e-07	***	GE_Fibrosis	
BEC	2.09e-07	***	Histochemistry	H
Ccl5	2.78e-07	***	GE_Cytokines	
Col1a1	4.40e-07	***	GE_Fibrosis	
Ifnar1	7.71e-07	***	GE_Cytokines	
S100a4	9.19e-07	***	Histochemistry	H
Sparc	1.07e-06	***	GE_Fibrosis	
Cyp2e1	1.50e-06	***	GE_Fibrosis	
Cxcr2	1.75e-06	***	GE_Cytokines	
Ccr3	1.91e-06	***	GE_Cytokines	
Cd69	2.73e-06	***	GE_Cytokines	
Cyp2c29	2.93e-06	***	GE_ADME	
Gsta2	3.88e-06	***	GE_ADME	
Tnf	4.36e-06	***	GE_Cytokines	
Gdf2	5.95e-06	***	GE_Fibrosis	
Il1b	7.29e-06	***	GE_Cytokines	
Ifng	7.51e-06	***	GE_Cytokines	
Osm	7.51e-06	***	GE_Cytokines	
Ccl3	8.53e-06	***	GE_Cytokines	
Il13	9.29e-06	***	GE_Cytokines	
Cxcr1	1.07e-05	***	GE_Cytokines	
Cyp2c37	1.09e-05	***	GE_ADME	
Cd14	1.15e-05	***	GE_Cytokines	
Col3a1	2.02e-05	***	GE_Fibrosis	
Tnfrsf1a	3.33e-05	***	GE_Cytokines	
Il2	4.84e-05	***	GE_Cytokines	
Ifnb1	4.86e-05	***	GE_Cytokines	
Egf	4.88e-05	***	GE_Cytokines	
Il28b	4.88e-05	***	GE_Cytokines	
Il10	4.88e-05	***	GE_Cytokines	
Il4	4.88e-05	***	GE_Cytokines	
Slc10a1	5.26e-05	***	GE_ADME	
Timp2	6.24e-05	***	GE_Fibrosis	
Cxcl3	6.82e-05	***	GE_Cytokines	
Ccl8	1.23e-04	***	GE_Cytokines	
Ctgf	1.32e-04	***	GE_Fibrosis	
Gstp1	1.38e-04	***	GE_ADME	
Ppara	1.63e-04	***	GE_ADME	
Ifnar2	1.82e-04	***	GE_Cytokines	
Il6	2.23e-04	***	GE_Cytokines	
Il17a	2.43e-04	***	GE_Cytokines	
Bad	3.84e-04	***	GE_Fibrosis	
Timp1	4.30e-04	***	GE_Fibrosis	
Cdh1	4.48e-04	***	GE_Fibrosis	
Cebpa	4.93e-04	***	GE_ADME	
alpha.SMA	5.02e-04	***	Histochemistry	H
NPC	5.28e-04	***	Histochemistry	H
Cdh2	5.38e-04	***	GE_Fibrosis	
Sirius.red	7.59e-04	***	Histochemistry	H
Pdgfb	7.68e-04	***	GE_Fibrosis	
Il6st	8.71e-04	***	GE_Cytokines	
Fn1	1.21e-03	**	GE_Fibrosis	
Mki67	1.46e-03	**	GE_Fibrosis	

Ifna1	1.46e-03	**	GE_Cytokines	
Egfr	1.59e-03	**	GE_Cytokines	
Kupffer	1.95e-03	**	Histochemistry	H
Tnc	2.04e-03	**	GE_Fibrosis	
Ugt1a1	2.75e-03	**	GE_ADME	
Sult1a1	2.98e-03	**	GE_ADME	
GLDH	4.28e-03	**	Biochemistry	B
Notch1	4.45e-03	**	GE_Fibrosis	
Met	4.97e-03	**	GE_Cytokines	
Cyp7a1	9.83e-03	**	GE_ADME	
Cyp24a1	9.88e-03	**	GE_ADME	
Tgfb2	1.08e-02	*	GE_Fibrosis	
Birc5	1.69e-02	*	GE_Fibrosis	
Actb.y	1.91e-02	*	GE_Cytokines	
Bak1	2.73e-02	*	GE_Fibrosis	
Bax	2.89e-02	*	GE_Fibrosis	
Rarres1	3.57e-02	*	GE_Fibrosis	
bile.infarcts	3.78e-02	*	Histochemistry	H
Cyp3a11	5.30e-02	.	GE_ADME	
Sult1b1	5.74e-02	.	GE_ADME	
Cyp4a10	6.79e-02	.	GE_ADME	
Pparg	6.79e-02	.	GE_Fibrosis	
Hk2	7.50e-02	.	GE_ADME	
ALT	8.79e-02	.	Biochemistry	B
Smad6	9.49e-02	.	GE_Fibrosis	
Cyp2b10	1.17e-01		GE_ADME	
HC	1.27e-01		Histochemistry	H
Il1rn	1.35e-01		GE_Cytokines	
Nes	1.37e-01		GE_Fibrosis	
Nfkbia	1.57e-01		GE_ADME	
Rps18	1.68e-01		GE_Fibrosis	
Cxcl15	2.18e-01		GE_ADME	
Socs3	2.43e-01		GE_ADME	
Vdr	2.43e-01		GE_ADME	
Edn1	2.43e-01		GE_Fibrosis	
Nr2f1	2.63e-01		GE_ADME	
Abcg2	2.82e-01		GE_ADME	
Nr1i3	2.82e-01		GE_ADME	
Hnf4a	2.88e-01		GE_ADME	
Ptgs2	3.43e-01		GE_ADME	
Bcl2l11	3.43e-01		GE_Fibrosis	
Socs1	3.46e-01		GE_ADME	
Pten	3.66e-01		GE_Fibrosis	
Actb.x	3.90e-01		GE_ADME	
Cxcl2	4.06e-01		GE_Cytokines	
Xiap	4.06e-01		GE_Fibrosis	
Pde4a	4.42e-01		GE_Fibrosis	
Dpyd	4.43e-01		GE_ADME	
Cxcl1	4.43e-01		GE_Cytokines	
Lama1	4.57e-01		GE_Fibrosis	
Col8a1	5.07e-01		GE_Fibrosis	
Prom1	5.54e-01		GE_Fibrosis	
Actb	5.54e-01		GE_Fibrosis	
Ahr	5.57e-01		GE_ADME	
Nr2f2	5.57e-01		GE_ADME	
Nos2	5.82e-01		GE_ADME	
Notch3	7.26e-01		GE_Fibrosis	
Cebpd	7.29e-01		GE_ADME	
Hmox1	7.69e-01		GE_ADME	

```
Cyp2d22      8.36e-01            GE_ADME
Igf1         9.78e-01        GE_Fibrosis
Fasl         9.78e-01        GE_Fibrosis
Cyp2c39      1.00e+00            GE_ADME
Nr3c1        1.00e+00            GE_ADME
Abcb1a       1.00e+00            GE_ADME
Nr1h3        1.00e+00            GE_ADME
Sod2         1.00e+00            GE_ADME
Rxra         1.00e+00            GE_ADME
Abcc2        1.00e+00            GE_ADME
Por          1.00e+00            GE_ADME
Nr1i2        1.00e+00            GE_ADME
Cebpb        1.00e+00            GE_ADME
Wisp1        1.00e+00        GE_Fibrosis
Pde4b        1.00e+00        GE_Fibrosis
Smad7        1.00e+00        GE_Fibrosis
Ch25h        1.00e+00        GE_Fibrosis
Pde4d        1.00e+00        GE_Fibrosis
Acta2        1.00e+00        GE_Fibrosis
Col4a3       1.00e+00        GE_Fibrosis
Col6a6       1.00e+00        GE_Fibrosis
albumin      1.00e+00       Biochemistry      B
```

Filter factors

The factors are now filtered based on the respective acceptance level of the ANOVA, with the cutoff for the adjusted p-value, i.e. all factors with a ANOVA with $p_{adj} \geq p_{accept}$ are filtered out. The filtered raw data is available as `BDLdata.fil`, the filtered mean data is stored as `BDLmean.fil`.

```
p.accept = 0.05  # acceptance level
anova.accept = (df.anova$p.holm < p.accept)  # accepted subset
# subset of filtered data
BDLdata.fil <- BDLdata[, anova.accept]
BDLmean.fil <- BDLdata[, anova.accept]

# accepted
table(anova.accept)  # 64 rejected / 90 accepted (adjusted)
```

```
anova.accept
FALSE  TRUE
   63    90
```

```
# which factors are accepted in which category
fil_tab <- data.frame(
  table(BDLfactors$ftype[anova.accept]),
  table(BDLfactors$ftype),
  round(table(BDLfactors$ftype[anova.accept])/table(BDLfactors$ftype), 2)
)
fil_tab <- fil_tab[, c('Var1', 'Freq', 'Freq.1', 'Freq.2')]
names(fil_tab) <- c('Category', 'Accepted', 'All', 'Percent')
# overview of filtered factors
print(fil_tab)
```

```
        Category Accepted All Percent
```

```
1   Biochemistry        2   4    0.50
2        GE_ADME       14  47    0.30
3   GE_Cytokines       44  47    0.94
4    GE_Fibrosis       22  46    0.48
5 Histochemistry        8   9    0.89
```

```
rm(fil_tab)
```

Results: Based on the adjusted p-values the data set was reduced from original 153 factors to 90. Almost all `Cytokines` genes (inflammation panel) were retained in the data set whereas many of the `ADME` and `Fibrosis` genes are filtered. All subsequent analyses are performed on the filtered data set, which is depicted in the following heatmap.

Heatmap of filtered time course data

We now plot the heatmap of time courses only for the subset of filtered factors.

```
# vertical time separators
v_lines <- ((1:Nt)*Nr+0.5)

timecourse_heatmap_filtered <- function(){
  # prepare data with row names
  dtmp <- BDLdata.fil
  rownames(dtmp) <- paste(rownames(BDLsamples), BDLsamples$time_fac, sep=" ")

  # color definitions
  hmap_colors <- HeatmapColors()
  colorset <- brewer.pal(length(factor_types), "Set2")
  color.map <- function(factor_id) {
    return(
      colorset[which(factor_types==BDLfactors$ftype[which(BDLfactors$id==factor_id)])]
  )}
  factorColors <- unlist(lapply(colnames(BDLdata.fil), color.map))
  # heatmap
  heatmap.2(t(as.matrix(dtmp)), col=hmap_colors(100), scale="row", dendrogram="none",
            Rowv=NULL, Colv=NULL,
            key=TRUE, trace="none", cexRow=0.5, keysize=0.8, density.info="none",
            RowSideColors=factorColors,
            add.expr=abline(v=v_lines, col="black", lwd=0.5),
            main="ANOVA Filtered BDL factors")
            # xlab="sample", ylab="factor")
  # legend
  legend("left",
      inset=c(-0.03,0),
      legend = rev(factor_types), # category labels
      col = rev(colorset),   # color key
      lty= 1,                # line style
      lwd = 10,              # line width
      cex = 0.7,
      bty="n"
  )
}
```

```r
# plot to file
pdf(file.path(resultsPath, "control", "timecourse_heatmap_filtered.pdf"),
    width=10, height=10, pointsize=12)
timecourse_heatmap_filtered()
invisible(dev.off())
```

```r
# plot to report
timecourse_heatmap_filtered()
```

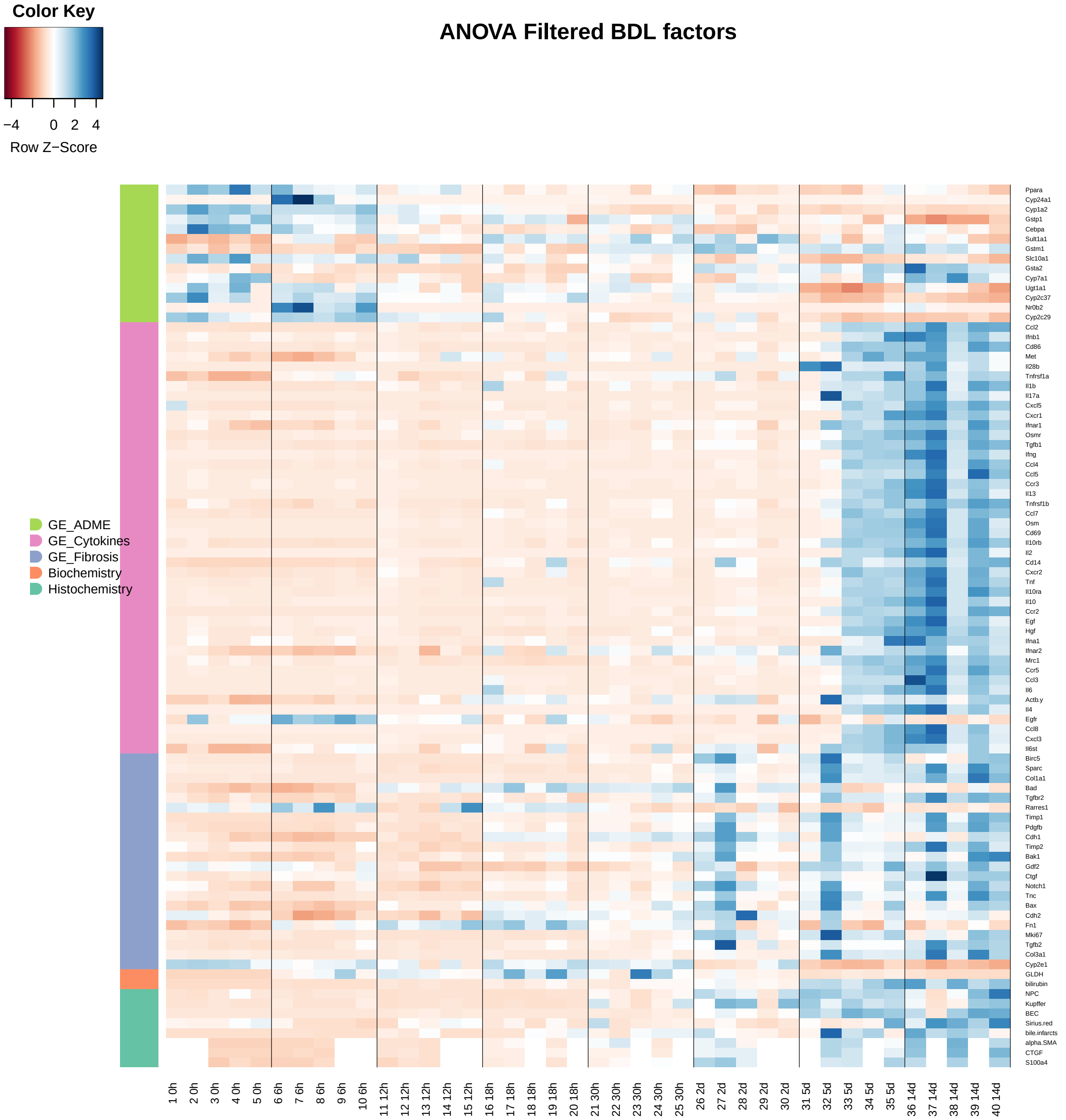

Figure: Filtered timecourse data. Plot of ANOVA filtered data set with processing analog to Figure above.

t-test for initial phase

We were in addition interested in the factors changed only in the initial phase, i.e. between control and the 6h time point. Therefore, a t-test was performed to find significantly changed factors in the initial phase after BDL.

```
# t-test for the initial phase changes
calculate_inital_phase_changes <- function(){
  # init vectors
  p.t_test <- rep(NA, ncol(BDLdata))
  names(p.t_test) <- colnames(BDLdata)
  up_down <- rep(NA, ncol(BDLdata))
  names(up_down) <- colnames(BDLdata)

  for (name in colnames(BDLdata)){
    # data for control (0h) and initial response (6h)
    d0 <- BDLdata[BDLsamples$time_fac == "0h", name]
    d6 <- BDLdata[BDLsamples$time_fac == "6h", name]
    # remove NA (for immunostainings Nr=3)
    d0 <- d0[!is.na(d0)]
    d6 <- d6[!is.na(d6)]
    # unpaired two.sided t-test
    t.test.res <- t.test(d0, d6, alternative="two.sided", var.equal=FALSE)
    p.t_test[name] <- t.test.res$p.value
    # going up or down
    up_down[name] <- "-"
    if (mean(d6) > mean(d0)){
      up_down[name] <- "up"
    } else if (mean(d6) < mean(d0)){
      up_down[name] <- "down"
    }
  }
  # data.frame for t-test results
  p.df <- data.frame(p.value=p.t_test, up_down=up_down)
  p.df$sig <- sapply(p.df$p.value, significant_code)
  rownames(p.df) <- colnames(BDLdata)

  # sort by p.value
  p.df.ordered <- p.df[order(p.df$p.value),]

  return(p.df.ordered)
}

# top up and down regulated in initial phase
p.df.ordered <- calculate_inital_phase_changes()
p.df.up <- p.df.ordered[p.df.ordered$p.value<0.05 & p.df.ordered$up_down=="up", ]
p.df.down <- p.df.ordered[p.df.ordered$p.value<0.05 & p.df.ordered$up_down=="down", ]
```

The top up- and down-regulated factors in the initial phase (independent from the ANOVA analysis, i.e. of all factors in data set) are

```
# top up
print(p.df.up)
```

```
               p.value up_down sig
Tnfrsf1a  2.418075e-05      up ***
Il6st     4.777944e-04      up ***
Osmr      5.635117e-04      up ***
Fn1       2.057930e-03      up  **
Cd14      4.500421e-03      up  **
ALT       5.488831e-03      up  **
bilirubin 9.221295e-03      up  **
Nr0b2     9.693272e-03      up  **
Ctgf      1.026407e-02      up   *
Cxcl1     1.159292e-02      up   *
Timp1     1.196340e-02      up   *
Egfr      1.444607e-02      up   *
Cyp4a10   2.288889e-02      up   *
Cxcl2     2.507493e-02      up   *
GLDH      2.896681e-02      up   *
Hmox1     2.913971e-02      up   *
Socs3     3.604221e-02      up   *
Sult1a1   3.632014e-02      up   *
```

```
# top down
print(p.df.down)
```

```
               p.value up_down sig
Cdh2       0.005994558    down  **
Pde4a      0.010806348    down   *
Sirius.red 0.014252420    down   *
Pten       0.014853870    down   *
Col3a1     0.017104924    down   *
Nes        0.026669560    down   *
Il28b      0.027571476    down   *
Cdh1       0.027687939    down   *
Tgfb1      0.032921400    down   *
Il10ra     0.035845273    down   *
Cyp2e1     0.038398167    down   *
Cyp7a1     0.038785457    down   *
Xiap       0.041507273    down   *
```

Results: In the initial phase more more up-regulations than down-regulations are observed.

Correlation analysis

For the correlation analysis between factors and the subsequent cluster analysis a correlation measure for time series data (Son2008) in combination with Complete-Linkage hierarchical clustering was used. This combination of methods provided the best enrichments on gene-expression time-series in a recent comparisons of methods (Jaskowiak2014, Jaskowiak2013).

The calculation of correlation coefficients between factors i and j ($i, j = 1, ..., N_p$) was performed using the slightly modified correlation coefficient based similarity measure developed for clustering of time-course data. $Y_{i,j}^{S2}$ and $Y_{i,j}^{R2}$ are hereby linear combinations of (i) a classical correlation part based on Spearman correlation $S_{i,j}^*$ in case of $Y_{i,j}^{S2}$ or Pearson $R_{i,j}^*$ in case of $Y_{i,j}^{R2}$, (ii) a component $A_{i,j}^*$ accounting for the similarity in changes between two time courses, (iii) a component $M_{i,j}^*$ comparing the location of minimum and maximum values of the time course (see (Son2008) for definitions)

$$Y_{i,j}^{S2} = w_1 S_{i,j}^* + w_2 A_{i,j}^* + w_3 M_{i,j}^*$$

$$Y_{i,j}^{R2} = w_1 R_{i,j}^* + w_2 A_{i,j}^* + w_3 M_{i,j}^*$$

$R_{i,j}^*$ and $S_{i,j}^*$ are hereby calculated on the individual data points for the factors i and j, $A_{i,j}^*$ and $M_{i,j}^*$ on the mean time courses averaged over the N_r repeated measurements. Throughout the analysis the following weights were used $w_1 = 0.5$, $w_2 = 0.3$, $w_3 = 0.2$.

In the calculation of the change component we used a Spearman correlation based measure (A^{**}) instead of the originally proposed Pearson measure (A^*) resulting in the correlation scores $Y_{i,j}^{S3}$ and $Y_{i,j}^{S3}$

$$Y_{i,j}^{S3} = w_1 S_{i,j}^* + w_2 A_{i,j}^{**} + w_3 M_{i,j}^*$$

$$Y_{i,j}^{R3} = w_1 R_{i,j}^* + w_2 A_{i,j}^{**} + w_3 M_{i,j}^*$$

Herein, $A_{i,j}^{**}$ calculates the correlation of changes between factors i and j based on Spearman correlation analog $A_{i,j}^*$ as

$$A_{i,j}^{**} = (S(d_i, d_j) + 1)/2$$
$$A_{i,j}^{*} = (R(d_i, d_j) + 1)/2$$

The reason for this adaption was that initial analysis showed a strong dependency of the change components on outliers.

All calculated correlation scores Y^S and Y^R are transformed from [0, 1] to [-1, 1] via

$$Y_{norm}^S = 2(Y^S - 0.5)$$

$$Y_{norm}^R = 2(Y^R - 0.5)$$

In addition Y^S and Y^R Pearson (R) and Spearman (S) correlations were calculated for comparison.

```
suppressPackageStartupMessages(library(corrplot))
dir.create(file.path(resultsPath, 'correlation'), showWarnings=FALSE)

# list of calculated correlation methods
correlation_methods <- c("pearson", "spearman", "ys1", "ys2", "ys3", "yr1", "yr2", "yr3")

# calculated correlation matrices are stored in cor.matrices
cor.matrices <- vector("list", length=length(correlation_methods))
names(cor.matrices) <- correlation_methods
```

Pearson & Spearman correlation

To get an overview over the correlation structure of the data set, we first calculated Pearson $R_{i,j}$ and Spearman $S_{i,j}$ correlation were calculated for the subset of filtered factors.

```
# Spearman and Pearson on individual data points
cor.matrices$pearson <- cor(BDLdata.fil, method="pearson", use="pairwise.complete.obs")
cor.matrices$spearman <- cor(BDLdata.fil, method="spearman", use="pairwise.complete.obs")
```

Heatmap plot of the correlation matrices with factors in original order

```
# Heatmap of correlation matrix
correlation_heatmap <- function(method){
  data <- cor.matrices[[method]]
  if(is.null(data)){
    stop("Correlation matrix does not exist for method: ", method)
```

```
  }
  cor_colors <- HeatmapColors() # color palette for correlation (red - white - blue)
  heatmap.2(data, col=cor_colors(10), scale="none",
          key=TRUE, symkey=FALSE, trace="none", cexRow=0.8, cexCol=0.8,
          main=method,
          density.info="none", dendrogram="none",
          Rowv=NULL, Colv=NULL,
          keysize=0.8, key.xlab = method,
          #revC=TRUE,
          sepwidth=c(0.01,0.01),
          sepcolor="black",
          colsep=1:ncol(data),
          rowsep=1:nrow(data))
}
```

```
# Pearson correlation (no clustering)
correlation_heatmap(method="pearson")
```

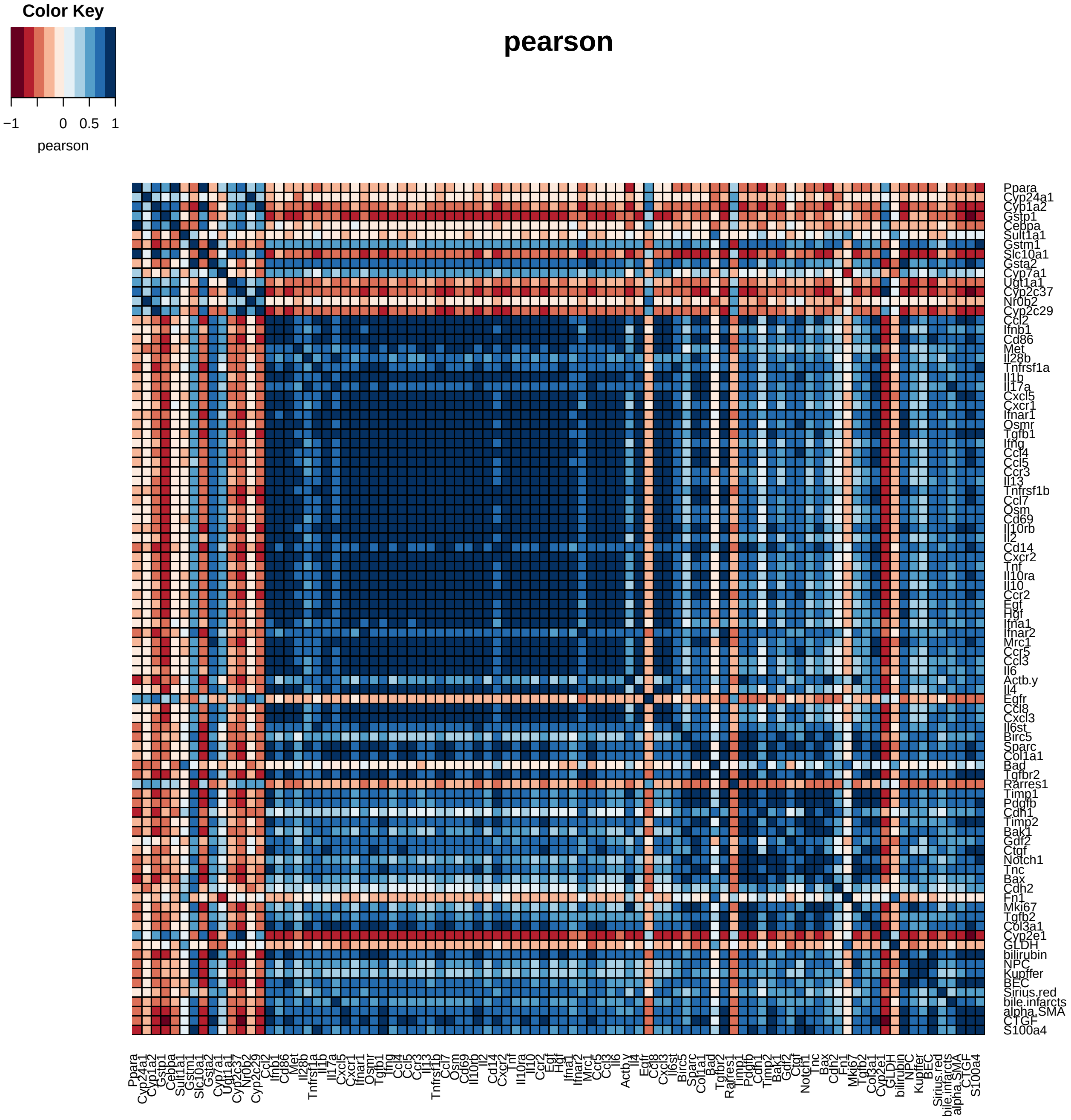

Figure: Heatmap of Pearson correlation. Correlation matrix based on Pearson correlation for filtered data set. No clustering was applied.

```r
# Spearman correlation (no clustering)
correlation_heatmap(method="spearman")
```

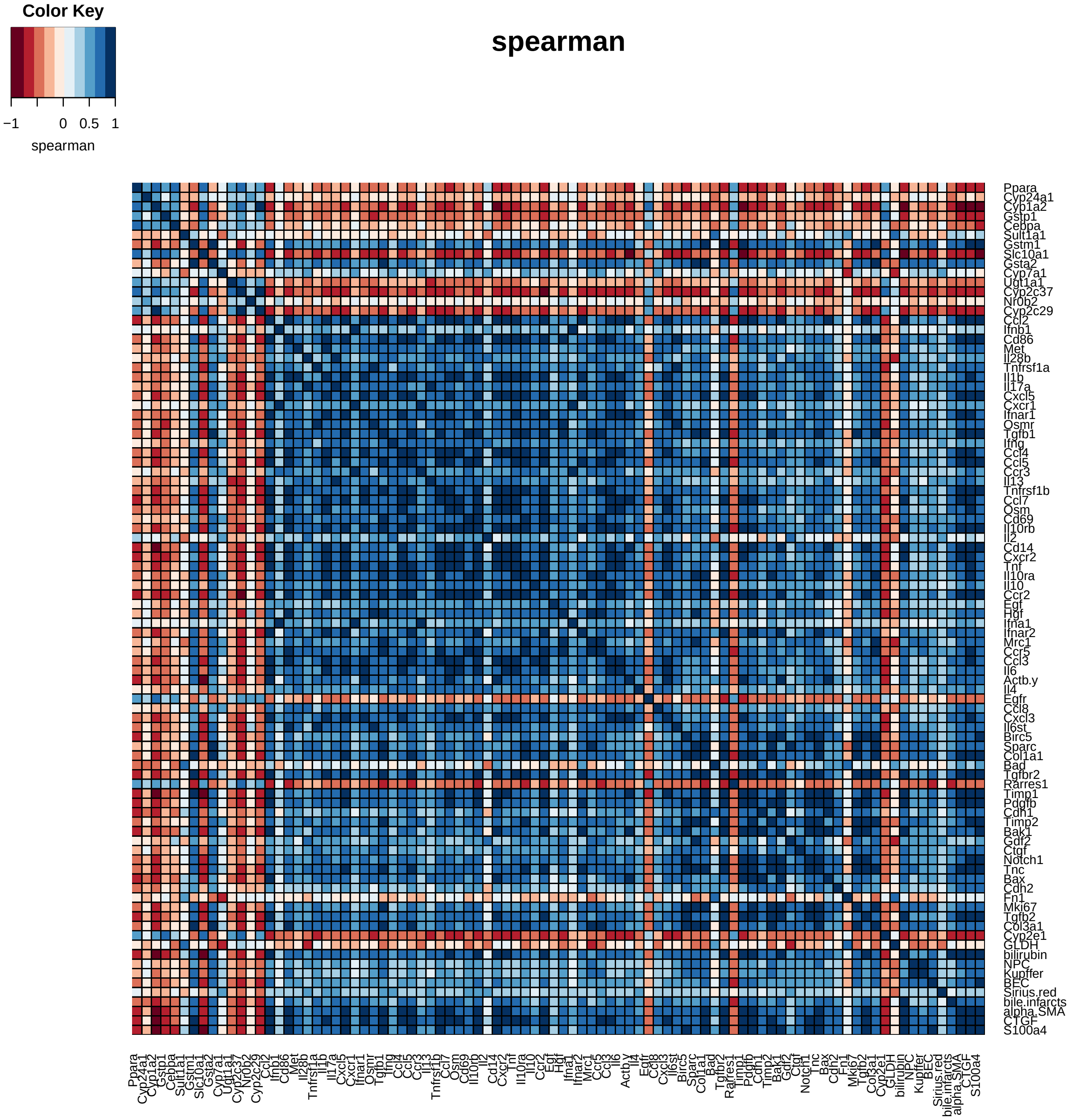

Figure: Heatmap of Spearman correlation. Correlation matrix based on Spearman correlation for filtered data set. No clustering was applied.

Results: The general correlation structure is similar between Pearson and Spearman, but the pearson correlation showed much more sensitive to outliers resulting in a large block of very high correlated factors. Spearman based correlatino coped much better with these outliers. Prelimary analysis of A^* showed similar problems, so we used A^{**} in Y^{S3} instead.

YS & YR correlation

Now the time-course based correlation measurements Y^{S1}, Y^{S2}, Y^{S3}, Y^{R1}, Y^{R2} and Y^{R3} are calculated for all factors in the filtered data set. For the calculation of the correlation matrix all pairwise correlations

between the factors were calculated. In the later analysis only the outlier robust Y^{S3} is used.

```
# weighting factors
w <- list(w1=0.5, w2=0.3, w3=0.2)

# calculate the YSR component matrices on the filtered data set (A, A*, A**, M, M*)
# all components are calculated on the mean data
ysr.res <- ysr.matrices(BDLmean.fil, BDLmean.time, use="pairwise.complete.obs")

# S* and R* (Pearson & Spearman correlation on individual data points)
cor.S_star <- (cor.matrices$spearman + 1)/2
cor.R_star <- (cor.matrices$pearson + 1)/2

# Calculate YS and YR scores based on the components
ysr_methods <- c("ys1", "ys2", "ys3", "yr1", "yr2", "yr3")
cor.ysr <- vector("list", length(ysr_methods))
names(cor.ysr) <- ysr_methods

# unnormalized correlations in [0, 1] as combination of weighted (S/R, A/A*/A**, M/M*)
cor.ysr$ys1 <- w$w1*cor.S_star + w$w2*ysr.res$A       + w$w3*ysr.res$M
cor.ysr$ys2 <- w$w1*cor.S_star + w$w2*ysr.res$A_star  + w$w3*ysr.res$M_star
cor.ysr$ys3 <- w$w1*cor.S_star + w$w2*ysr.res$A_star2 + w$w3*ysr.res$M_star

cor.ysr$yr1 <- w$w1*cor.R_star + w$w2*ysr.res$A       + w$w3*ysr.res$M
cor.ysr$yr2 <- w$w1*cor.R_star + w$w2*ysr.res$A_star  + w$w3*ysr.res$M_star
cor.ysr$yr3 <- w$w1*cor.R_star + w$w2*ysr.res$A_star2 + w$w3*ysr.res$M_star

# scaling of ysr correlation coefficient to interval [-1,1]
for (method in ysr_methods){
  cor.matrices[[method]] <- 2*(cor.ysr[[method]]-0.5)
}

# Save correlation matrices
save(cor.matrices, file=file.path(resultsPath, "data", "cor.matrices.Rdata"))
rm(cor.ysr, cor.R_star, cor.S_star, ysr.res, ysr_methods)
```

Create heatmap plots for all calculated correlation matrices

```
# create all correlation heatmaps on disk
hmap.settings <- list(width=10, height=10, pointsize=12)
for (method in correlation_methods){
  fname <- sprintf('cor.%s.pdf', method)
  pdf(file.path(resultsPath, "correlation", fname),
      width=hmap.settings$width, height=hmap.settings$height,
      pointsize=hmap.settings$pointsize)
  correlation_heatmap(method=method)
invisible(dev.off())
rm(method, fname)
}
```

```
# ys3 heatmap in report
correlation_heatmap(method="ys3")
```

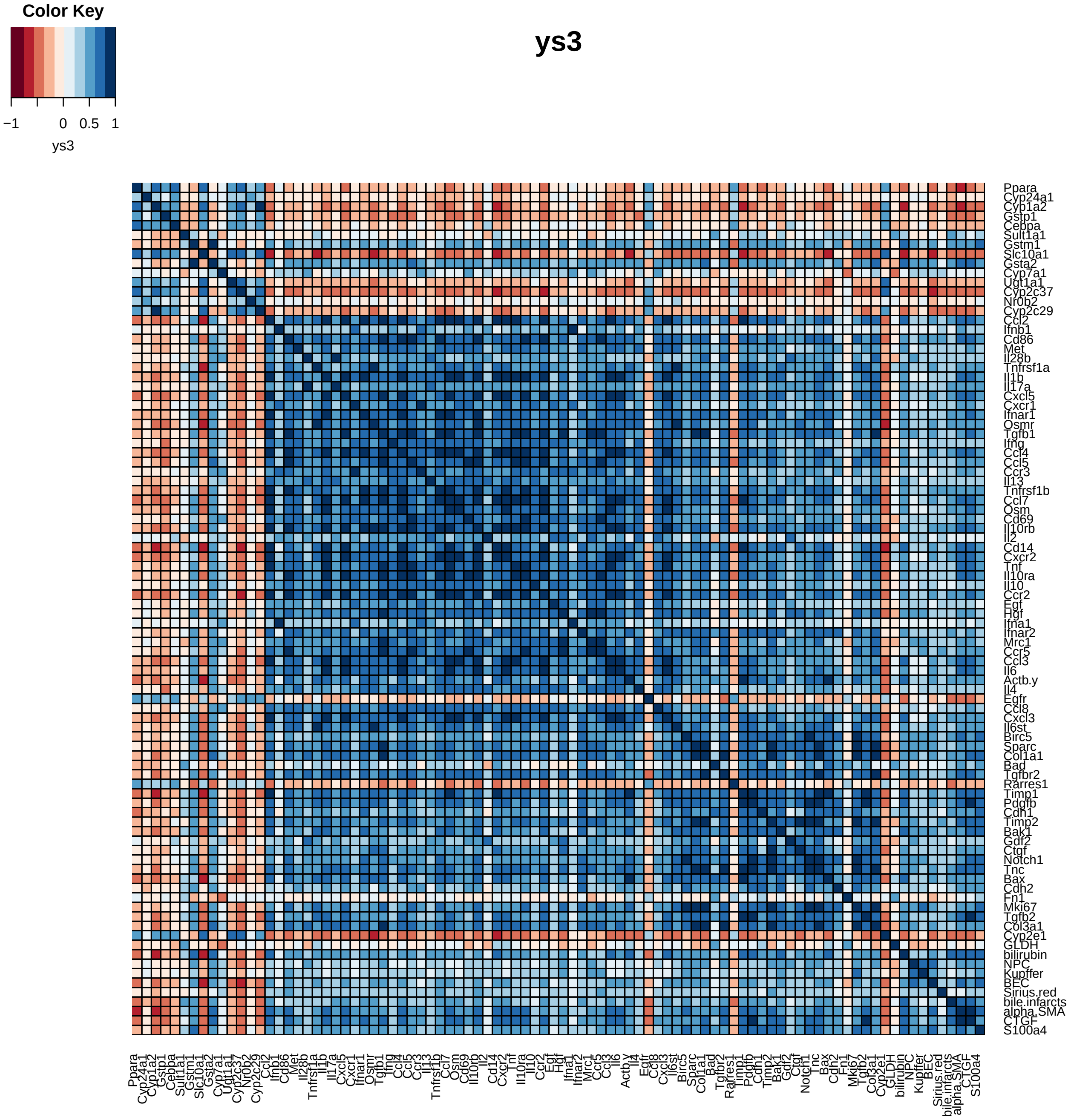

Figure: Heatmap of YS3 correlation. Correlation matrix based on YS3 correlation for filtered data set. No clustering was applied.

Hierarchical clustering

Clustering

Now clusters are calculated based on the calculated correlation matrices using hierarchical clustering with complete linkage. Hereby, the factors are grouped into a number of clusters with the correlation within each cluster being larger than between clusters. For a time course based correlation measure like Y^{S3} this effectivly

finds groups of factors which have similar time courses. The complete linkage method defines the cluster distance between two clusters to be the maximum distance between their individual components.

The resulting hierarchical clustering based on the given correlation matrix is split into `Ngroups=6` clusters (prelimenary analysis showed that a higher number of clusters results in clusters with only one member). The number of clusters was choosen, so that at least `>1` members exist per cluster.

```
hclust.res <- vector("list", length=length(correlation_methods))
names(hclust.res) <- correlation_methods

# Calculate hierarical clustering: N clusters for correlation based on method
calculate_clusters <- function(cor_method, N){
  # get correlation matrix
  cor.cluster <- cor.matrices[[cor_method]]
  # perform hierarchical clustering (complete linkage & Euclidion distance measure)
  hc <- hclust(dist(cor.cluster, method="euclidian"), method="complete")
  # cut into the clusters
  groups <- cutree(hc, k=Ngroups)
  groups.hc.order <- groups[hc$order]
  # store results
  return(list(hc=hc,
              groups=groups,
              groups.hc.order=groups.hc.order))
}

Ngroups <- 6   # number of clusters
for (method in correlation_methods){
  hclust.res[[method]] <- calculate_clusters(cor_method=method, N=Ngroups)
}
# save clustering
save(hclust.res, file=file.path(resultsPath, "data", "hclust.res.Rdata"))
rm(method)
```

In the next step the correlation matrices are plotted in combination with the clustering results (dendrogram) and the clusters as side colors.

```
# Create correlation heatmap with hierachical clustering results
correlation_heatmap_cluster <- function(method){
  # matrix and cluster results
  cor.cluster <- cor.matrices[[method]]
  hc.res <- hclust.res[[method]]
  hc <- hc.res$hc
  groups <- hc.res$groups
  # colors
  hmap_colors <- HeatmapColors()
  colorset <- brewer.pal(Ngroups, "Set1")
  color.map <- function(cluster_id) {return(colorset[cluster_id])}
  clusterColors <- unlist(lapply(groups, color.map))

  heatmap.2(cor.cluster, col=hmap_colors(10), scale="none",
          key=TRUE, symkey=FALSE, trace="none", cexRow=0.8, cexCol=0.8,
          main=method,
          density.info="none", dendrogram="column",
          Rowv=as.dendrogram(hc), Colv=as.dendrogram(hc),
```

```
          keysize=0.8,
          key.xlab=method,
          ColSideColors=clusterColors, revC=TRUE,
          sepwidth=c(0.01,0.01),
          sepcolor="black",
          colsep=1:ncol(cor.cluster),
          rowsep=1:nrow(cor.cluster),
          margins=c(12,8))
  legend("left", legend=paste("c", 1:6, sep=""),
         col= unlist(lapply(1:6, color.map)), pch=15, bty="n")
}

# plot to files
for(method in correlation_methods){
  pdf(file.path(resultsPath, "correlation", sprintf("cor.%s.hclust.pdf", method)),
      width=10, height=10, pointsize=12)
  correlation_heatmap_cluster(method=method)
  invisible(dev.off())
}
rm(method)
```

```
# plot ys3 clustering to report
correlation_heatmap_cluster(method="ys3")
```

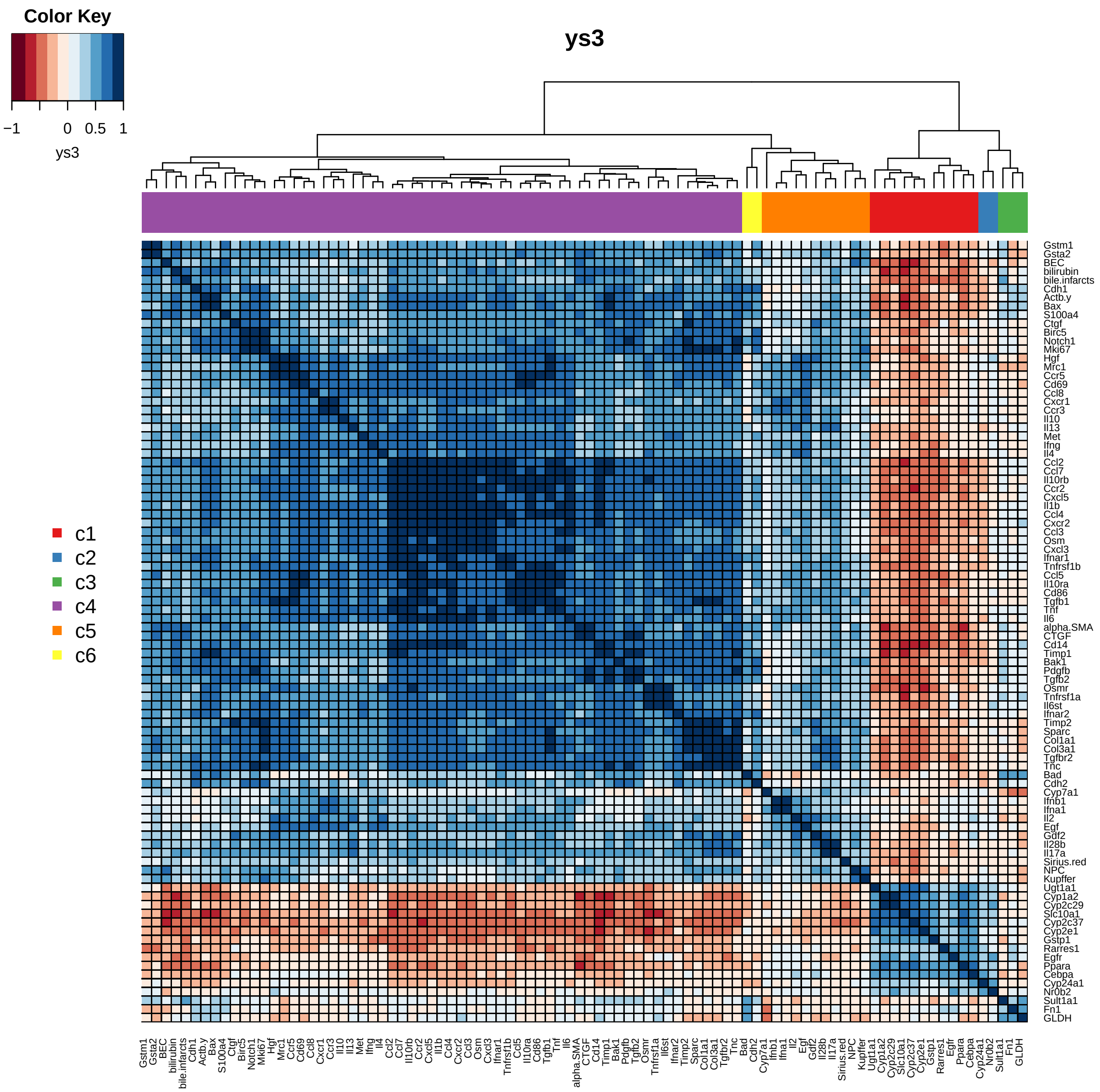

Figure: Heatmap of YS3 clustering. Correlation matrix based on YS3 correlation for filtered data set with hierarchical clustering results based on complete linkage.

The following factors are in the `ys3` time course clusters.

```
# print representatives of cluster
list_cluster_members <- function(method){
  groups <- hclust.res[[method]]$groups
  cat(sprintf("------------------------------------------\n"))
  cat(sprintf("Correlation method: *** %s ***\n", method))
  cat(sprintf("------------------------------------------\n"))
  for (k in 1:Ngroups){
    g <- groups[groups==k]
    cat(sprintf("Cluster %s (N=%s)\n", k, length(g)))
    print(names(g))
```

```r
    cat("\n")
    # print(paste(names(g), sep=", ", collapse =", " ))
  }
}
```

```r
list_cluster_members(method="ys3")
```

```
------------------------------------------
Correlation method: *** ys3 ***
------------------------------------------
Cluster 1 (N=11)
 [1] "Ppara"   "Cyp1a2"  "Gstp1"   "Cebpa"   "Slc10a1" "Ugt1a1"  "Cyp2c37"
 [8] "Cyp2c29" "Egfr"    "Rarres1" "Cyp2e1"

Cluster 2 (N=2)
[1] "Cyp24a1" "Nr0b2"

Cluster 3 (N=3)
[1] "Sult1a1" "Fn1"     "GLDH"

Cluster 4 (N=61)
 [1] "Gstm1"          "Gsta2"          "Ccl2"           "Cd86"
 [5] "Met"            "Tnfrsf1a"       "Il1b"           "Cxcl5"
 [9] "Cxcr1"          "Ifnar1"         "Osmr"           "Tgfb1"
[13] "Ifng"           "Ccl4"           "Ccl5"           "Ccr3"
[17] "Il13"           "Tnfrsf1b"       "Ccl7"           "Osm"
[21] "Cd69"           "Il10rb"         "Cd14"           "Cxcr2"
[25] "Tnf"            "Il10ra"         "Il10"           "Ccr2"
[29] "Hgf"            "Ifnar2"         "Mrc1"           "Ccr5"
[33] "Ccl3"           "Il6"            "Actb.y"         "Il4"
[37] "Ccl8"           "Cxcl3"          "Il6st"          "Birc5"
[41] "Sparc"          "Col1a1"         "Tgfbr2"         "Timp1"
[45] "Pdgfb"          "Cdh1"           "Timp2"          "Bak1"
[49] "Ctgf"           "Notch1"         "Tnc"            "Bax"
[53] "Mki67"          "Tgfb2"          "Col3a1"         "bilirubin"
[57] "BEC"            "bile.infarcts" "alpha.SMA"      "CTGF"
[61] "S100a4"

Cluster 5 (N=11)
 [1] "Cyp7a1"     "Ifnb1"      "Il28b"      "Il17a"      "Il2"
 [6] "Egf"        "Ifna1"      "Gdf2"       "NPC"        "Kupffer"
[11] "Sirius.red"

Cluster 6 (N=2)
[1] "Bad"  "Cdh2"
```

Correlations for non-transcript factors

In the following steps we look at specific parts of the correlation matrix. First we are interested in the correlations between transcripts and classical factors, i.e. biochemical and (immuno-)histochemical factors. The genes are filtered based on a cutoff for the correlation, i.e. columns in which no correlation coefficient `abs(value) >= cor.cutoff` exists are filtered out. Consequently, only transcripts are retained with a absolute correlation coeffient above this threshold.

```r
get_histological_correlations <- function(method="ys3", cor.cutoff=0.6){
  # correlation matrix
  cor_mat <- cor.matrices[[method]]

  # non-transcipt factors accepted by ANOVA
  hist_facs <- BDLfactors$id[anova.accept &
                (BDLfactors$ftype %in% c("Biochemistry", "Histochemistry"))]

  # get indices of these factors in correlation matrix
  hist_idx <- rep(NA, length(hist_facs))
  for (k in 1:length(hist_facs)){
    hist_idx[k] <- which(colnames(cor_mat) == hist_facs[k])
  }
  # subset of correlation matrix for histological markers (corresponding rows)
  hist_data <-  cor_mat[hist_idx, ]

  # filter columns by correlation cutoff
  col.accept <- rep(NA, ncol(hist_data))
  for (k in 1:ncol(hist_data)){
    # keep column if any abs(correlation) >= cutoff in the column
    col.accept[k] <- any( abs(hist_data[,k])>=cor.cutoff )
  }
  # accepted factors
  # print(table(col.accept))
  hist_accept <- hist_data[, col.accept]

  # sort by the hierarchical cluster ordering
  hist_gene_names <- colnames(hist_accept)[1:(ncol(hist_accept)-nrow(hist_accept))]

  # correlation based cluster for ordering
  hc.res <- hclust.res[[method]]

  # create sort index for gene names based on clustering
  sort_idx <- rep(NA, length(hist_gene_names))
  for (k in 1:length(hist_gene_names)){
    sort_idx[k] <- which(names(hc.res$groups.hc.order) == hist_gene_names[k])
  }
  # first the sort genes, at the end the self correlation of the histological factors
  hist_sorted <- hist_accept[, c(hist_gene_names[order(sort_idx)], rownames(hist_data))]
  return(hist_sorted)
}

histological_correlations <- get_histological_correlations(method="ys3")

# plot subset of correlations matrix to file
plot_histological_correlations <- function(hist_data){
  hmap_colors <- HeatmapColors()
  corrplot(hist_data, method="circle", type="full",
           tl.cex=0.7, tl.col="black", col=hmap_colors(10))
}

# plot to file
pdf(file.path(resultsPath, "correlation", "histological_correlations.pdf"),
```

```r
    width=10, height=4, pointsize=12)
plot_histological_correlations(histological_correlations)
invisible(dev.off())
```

```r
# print correlation values
options(width=200)
print(round(t(histological_correlations), digits=2))
```

```
               GLDH bilirubin   NPC Kupffer   BEC Sirius.red bile.infarcts alpha.SMA  CTGF S100a4
Gstm1         -0.02      0.61  0.42    0.40  0.43       0.10          0.59      0.55  0.57   0.70
Gsta2         -0.21      0.61  0.51    0.41  0.46       0.19          0.59      0.64  0.62   0.59
Cdh1           0.24      0.46  0.25    0.34  0.35       0.18          0.42      0.54  0.54   0.61
Actb.y         0.22      0.61  0.29    0.33  0.47       0.29          0.50      0.46  0.60   0.52
Bax            0.23      0.61  0.31    0.35  0.42       0.38          0.44      0.48  0.55   0.59
Birc5         -0.04      0.57  0.53    0.50  0.42       0.36          0.41      0.61  0.59   0.72
Notch1        -0.04      0.52  0.43    0.50  0.31       0.26          0.39      0.53  0.61   0.71
Mki67         -0.15      0.50  0.59    0.61  0.47       0.35          0.39      0.55  0.57   0.68
Ccl2           0.17      0.66  0.25    0.25  0.52       0.36          0.54      0.61  0.72   0.46
Ccl7           0.15      0.60  0.18    0.22  0.49       0.32          0.48      0.62  0.67   0.48
Il10rb         0.00      0.57  0.31    0.33  0.50       0.40          0.46      0.64  0.70   0.50
Ccr2           0.03      0.56  0.29    0.29  0.53       0.33          0.49      0.59  0.74   0.46
Cxcl5          0.00      0.54  0.26    0.23  0.49       0.34          0.43      0.73  0.75   0.53
Il1b           0.07      0.54  0.18    0.17  0.38       0.37          0.37      0.63  0.71   0.48
Ccl4           0.13      0.55  0.16    0.22  0.45       0.33          0.42      0.61  0.68   0.46
Cxcr2          0.22      0.55  0.15    0.15  0.44       0.33          0.42      0.61  0.71   0.44
Ccl3           0.18      0.62  0.16    0.17  0.45       0.23          0.53      0.62  0.69   0.50
Osm            0.19      0.59  0.15    0.14  0.38       0.37          0.45      0.59  0.63   0.51
Cxcl3          0.18      0.61  0.12    0.18  0.41       0.36          0.44      0.53  0.57   0.47
Ifnar1         0.11      0.56  0.21    0.23  0.36       0.30          0.44      0.56  0.67   0.49
Il10ra        -0.15      0.46  0.31    0.30  0.39       0.33          0.38      0.62  0.68   0.44
Cd86          -0.07      0.45  0.39    0.41  0.46       0.40          0.32      0.62  0.72   0.44
Tgfb1         -0.14      0.48  0.41    0.40  0.46       0.38          0.39      0.60  0.68   0.48
Tnf            0.00      0.49  0.25    0.25  0.34       0.31          0.40      0.60  0.69   0.49
Il6            0.03      0.51  0.13    0.20  0.40       0.25          0.37      0.65  0.61   0.52
Cd14           0.21      0.67  0.29    0.21  0.50       0.37          0.47      0.67  0.77   0.50
Timp1          0.12      0.67  0.32    0.34  0.50       0.27          0.57      0.73  0.73   0.64
Bak1           0.08      0.60  0.43    0.40  0.40       0.35          0.41      0.56  0.67   0.60
Pdgfb          0.08      0.62  0.33    0.37  0.41       0.30          0.48      0.69  0.80   0.72
Tgfb2          0.11      0.58  0.30    0.33  0.38       0.28          0.51      0.73  0.82   0.72
Timp2         -0.24      0.51  0.47    0.39  0.37       0.42          0.38      0.63  0.62   0.68
Col1a1        -0.22      0.55  0.53    0.38  0.48       0.38          0.42      0.59  0.61   0.66
Tgfbr2        -0.10      0.59  0.47    0.31  0.37       0.31          0.46      0.69  0.78   0.59
Tnc           -0.10      0.54  0.43    0.37  0.40       0.33          0.42      0.70  0.73   0.67
Cyp1a2        -0.15     -0.60 -0.11   -0.05 -0.44      -0.20         -0.56     -0.70 -0.60  -0.45
Slc10a1       -0.17     -0.62 -0.38   -0.29 -0.66      -0.37         -0.50     -0.54 -0.55  -0.44
Cyp2c37        0.03     -0.55 -0.41   -0.25 -0.66      -0.41         -0.49     -0.59 -0.55  -0.44
Ppara         -0.22     -0.47 -0.18   -0.05 -0.49      -0.05         -0.41     -0.61 -0.59  -0.37
GLDH           1.00      0.18 -0.30   -0.20 -0.02      -0.10          0.14     -0.04 -0.06   0.08
bilirubin      0.18      1.00  0.39    0.26  0.49       0.35          0.71      0.65  0.69   0.62
NPC           -0.30      0.39  1.00    0.75  0.62       0.32          0.25      0.41  0.46   0.54
Kupffer       -0.20      0.26  0.75    1.00  0.57       0.30          0.10      0.27  0.25   0.47
BEC           -0.02      0.49  0.62    0.57  1.00       0.36          0.39      0.60  0.56   0.62
Sirius.red    -0.10      0.35  0.32    0.30  0.36       1.00          0.06      0.20  0.21   0.38
bile.infarcts  0.14      0.71  0.25    0.10  0.39       0.06          1.00      0.61  0.63   0.44
alpha.SMA     -0.04      0.65  0.41    0.27  0.60       0.20          0.61      1.00  0.84   0.69
CTGF          -0.06      0.69  0.46    0.25  0.56       0.21          0.63      0.84  1.00   0.60
S100a4         0.08      0.62  0.54    0.47  0.62       0.38          0.44      0.69  0.60   1.00
```

```
options(width=75)
```

```
plot_histological_correlations(histological_correlations)
```

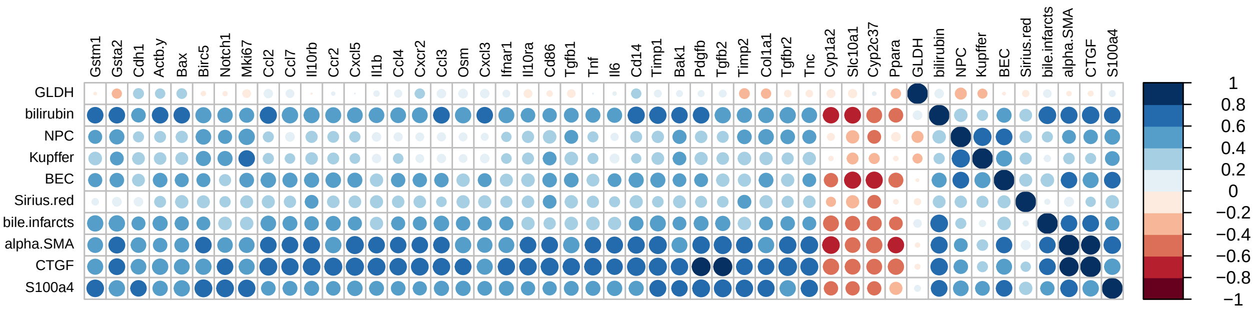

Figure: Correlations to classical factors. Correlations between transcripts and classical factors, i.e. biochemical and (immuno-)histochemical factors. The genes are filtered based on a cutoff for the correlation, i.e. columns in which no correlation coefficient abs(value) >= cor.cutoff exists are filtered out.

Top correlations for classical factors

In the next step the top correlations of every classical factor, i.e. (immuno-)histochemical (H) and biochemical (B) factors, are calculated, with the top correlations being sorted by absolute correlation values.

```
plot_hist_topcors <- function(method="ys3", labels=TRUE, mfrow=c(10,1),
                              only_RNA=FALSE, Ntop=10){
  hist_facs <- rownames(histological_correlations)
  cor.mat <- cor.matrices[[method]]

  # store the results
  top.correlations <- vector("list", length=length(hist_facs))
  names(top.correlations) <- hist_facs

  par(mfrow=mfrow)
  hmap_colors <- HeatmapColors()
  for (name in hist_facs){
    if (only_RNA==TRUE){
      # get which which are not classical factors
      v <- cor.mat[!(colnames(cor.mat) %in% hist_facs), name]
    } else {
      # get all factors (including other non-RNA factors)
      v <- cor.mat[, name]
    }
    # sort by absolute correlation
    v.sorted <- rev(v[order(abs(v))])
    # and get the Ntop values without the self-correlation (idx=1)
    mv <- t(as.matrix(v.sorted[2:(Ntop+1)]))
    rownames(mv) <- c(name)
    # store
    top.correlations[[name]] <- mv

    # plot without labels to have identical size of figure
    if (labels==FALSE){
```

```
      rownames(mv) <- NULL
      colnames(mv) <- NULL
    }
    corrplot(mv, method="pie", type="full",
             tl.cex=1.0, tl.col="black", col=hmap_colors(100), insig="p-value", sig.level=-1,
             p.mat=mv, cl.pos="n")
  }
  par(mfrow=c(1,1))
  return(invisible(top.correlations))
}

# plot to file
# necessary to plot once with and without labels so that all the plots are scaled equally
pdf(file.path(resultsPath, "correlation", "histological_topcors_1.pdf"),
    width=10, height=10, pointsize=12)
plot_hist_topcors(method="ys3", labels=TRUE)
invisible(dev.off())
pdf(file.path(resultsPath, "correlation", "histological_topcors_2.pdf"),
    width=10, height=10, pointsize=12)
plot_hist_topcors(method="ys3", labels=FALSE)
invisible(dev.off())
```

```
top.correlations <- plot_hist_topcors(method="ys3", labels=TRUE, mfrow=c(5,2))
```

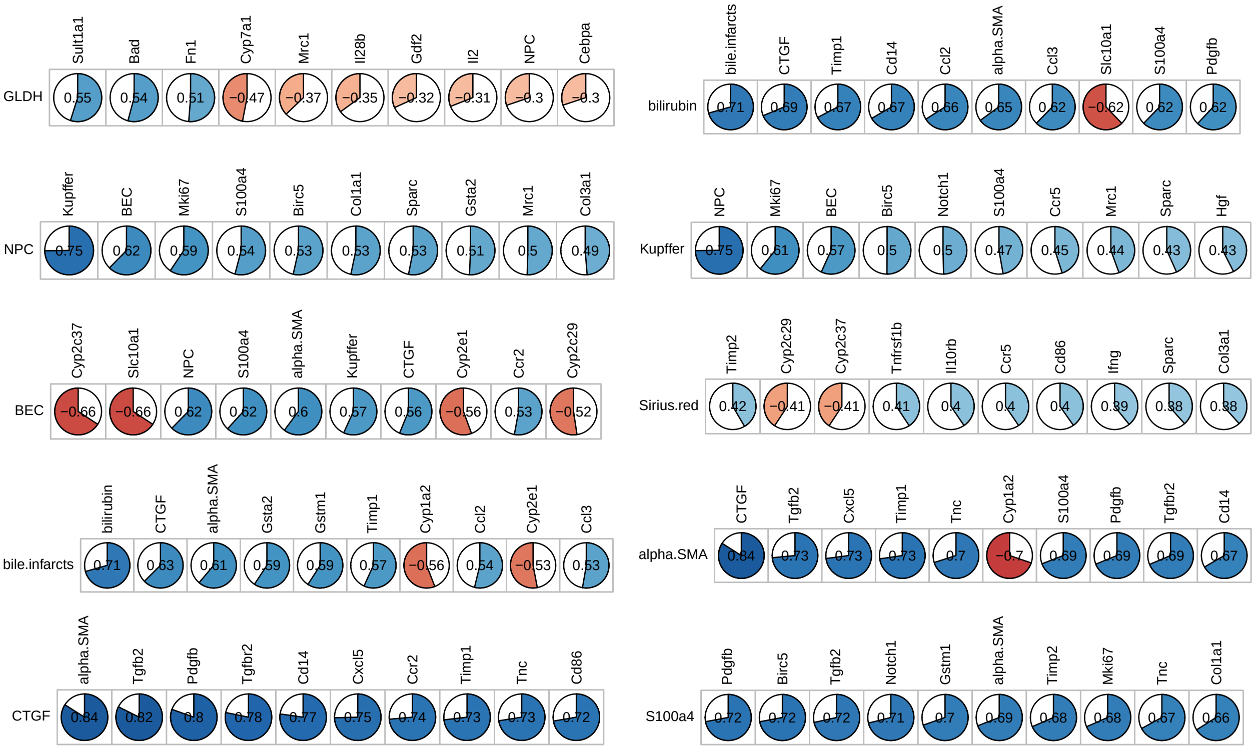

Figure: Top correlations to classical factors.

```
# print top correlations
options(width=200)
for (mv in top.correlations){
```

```
  print(round(mv, digits=2))
  cat("\n")
}
```

```
     Sult1a1  Bad  Fn1 Cyp7a1  Mrc1 Il28b  Gdf2   Il2  NPC Cebpa
GLDH    0.55 0.54 0.51  -0.47 -0.37 -0.35 -0.32 -0.31 -0.3  -0.3

          bile.infarcts CTGF Timp1 Cd14 Ccl2 alpha.SMA Ccl3 Slc10a1 S100a4 Pdgfb
bilirubin          0.71 0.69  0.67 0.67 0.66      0.65 0.62   -0.62   0.62  0.62

    Kupffer  BEC Mki67 S100a4 Birc5 Col1a1 Sparc Gsta2 Mrc1 Col3a1
NPC    0.75 0.62  0.59   0.54  0.53   0.53  0.53  0.51  0.5   0.49

         NPC Mki67  BEC Birc5 Notch1 S100a4 Ccr5 Mrc1 Sparc  Hgf
Kupffer 0.75  0.61 0.57   0.5    0.5   0.47 0.45 0.44  0.43 0.43

    Cyp2c37 Slc10a1  NPC S100a4 alpha.SMA Kupffer CTGF Cyp2e1 Ccr2 Cyp2c29
BEC   -0.66   -0.66 0.62   0.62       0.6    0.57 0.56  -0.56 0.53   -0.52

           Timp2 Cyp2c29 Cyp2c37 Tnfrsf1b Il10rb Ccr5 Cd86 Ifng Sparc Col3a1
Sirius.red  0.42   -0.41   -0.41     0.41    0.4  0.4  0.4 0.39  0.38   0.38

              bilirubin CTGF alpha.SMA Gsta2 Gstm1 Timp1 Cyp1a2 Ccl2 Cyp2e1 Ccl3
bile.infarcts      0.71 0.63      0.61  0.59  0.59  0.57  -0.56 0.54  -0.53 0.53

          CTGF Tgfb2 Cxcl5 Timp1 Tnc Cyp1a2 S100a4 Pdgfb Tgfbr2 Cd14
alpha.SMA 0.84  0.73  0.73  0.73 0.7   -0.7   0.69  0.69   0.69 0.67

     alpha.SMA Tgfb2 Pdgfb Tgfbr2 Cd14 Cxcl5 Ccr2 Timp1  Tnc Cd86
CTGF      0.84  0.82   0.8   0.78 0.77  0.75 0.74  0.73 0.73 0.72

       Pdgfb Birc5 Tgfb2 Notch1 Gstm1 alpha.SMA Timp2 Mki67  Tnc Col1a1
S100a4  0.72  0.72  0.72   0.71   0.7      0.69  0.68  0.68 0.67   0.66
```

```
options(width=75)
rm(mv)
```

Mean cluster time course

In the next step, we are interested in the time courses of the found clusters: What are the typical profiles found in the different clusters and which factors are in these clusters. For the comparison of individual factors against each other and against the mean time course of the cluster the factors were normalized. The normalization was hereby performed for every factor separately based on

$$f_k^{norm}(t_{i,r}) = \frac{f_k(t_{i,r}) - < f_k >}{max(f_k) - min(f_k)}$$

```
suppressPackageStartupMessages(library(matrixStats))
dir.create(file.path(resultsPath, 'cluster'), showWarnings=FALSE)

# normalize single factor
normalize_factor <- function(a, min.a, max.a, mean.a){
  res <- (a - mean.a)/(max.a - min.a)
}
# calculate min, max and mean for all single factors (normalization constants)
```

```
factor.norm <- data.frame(min=apply(BDLdata, 2, min, na.rm=TRUE),
                          max=apply(BDLdata, 2, max, na.rm=TRUE),
                          mean=apply(BDLdata, 2, mean, na.rm=TRUE))
# function for normalizing subset of BDL data with factor normalization constants.
normalize_BDLdata <- function(data, factor.norm){
  dnorm <- data
  for (name in colnames(data)){
    dnorm[, name] <- normalize_factor(a=dnorm[, name],
                                      min.a=factor.norm[name, "min"],
                                      max.a=factor.norm[name, "max"],
                                      mean.a=factor.norm[name, "mean"])
  }
  return(dnorm)
}

# normalize full data set
BDLdata.norm <- normalize_BDLdata(data=BDLdata, factor.norm=factor.norm)
# Calculate the mean of the normalized data set
BDLmean.norm <- bdl_mean_data(BDLdata.norm, BDLsamples)
```

With the normalized factor data the mean time course of the 6 clusters are calculated, i.e. the time course resulting from averaging over all members of the individual clusters. Additionally, the mean time course averaged over the repeats for all factors are plotted.

```
# Plot mean cluster with SD range and the individual representatives in the cluster.
plot_clusters_mean <- function(method){
  # clusters for correlation method
  hc.res <- hclust.res[[method]]
  groups.hc.order <- hc.res$groups.hc.order

  # par(mfrow=c(ceiling(sqrt(Ngroups)),ceiling(sqrt(Ngroups))))
  par(mfrow=c(2,3))
  steps <- 1:Nt  # time points
  for (k in 1:Ngroups){
    # get representatives of cluster
    g <- groups.hc.order[groups.hc.order==k]
    dgroup <- BDLmean.norm[names(g)]  # normalized mean data for cluster members

    # mean and sd for timepoints (i.e. over all factors in the cluster)
    g.mean <- rowMeans(as.matrix(dgroup), na.rm=TRUE)
    g.sd <- rowSds(as.matrix(dgroup), na.rm=TRUE)

    # plot sd range
    plot(factor(levels(BDLsamples$time_fac), levels=levels(BDLsamples$time_fac)),
         rep(-2, 8), type="n", xlab="", ylab="",
         xlim=c(1, Nt), ylim=1.1*c( min(min(dgroup, na.rm=TRUE), na.rm=TRUE),
                                   max(max(dgroup, na.rm=TRUE), na.rm=TRUE) ),
         main=sprintf("%s : Cluster %s (N=%s)", method, k, ncol(dgroup)))
    polygon(c(steps, rev(steps)), c(g.mean+g.sd, rev(g.mean-g.sd)),
            col = rgb(0,0,1,0.2), border = NA)

    # individual data
    for (name in names(g)){
```

```
      points(steps, dgroup[, name], pch=16, col="black")
      lines(steps, dgroup[, name], col=rgb(0.5,0.5,0.5,1.0), lwd=1)
    }
    # mean over factors in cluster
    lines(steps, g.mean, col="blue", lwd=2)
  }
  par(mfrow=c(1,1))
}

# plot mean clusters to file
for (method in correlation_methods){
  pdf(file.path(resultsPath, 'cluster', sprintf("%s_cluster_mean.pdf", method)),
        width=10, height=7.5, pointsize=12)
  plot_clusters_mean(method=method)
  invisible(dev.off())
}
```

```
plot_clusters_mean(method="ys3")
```

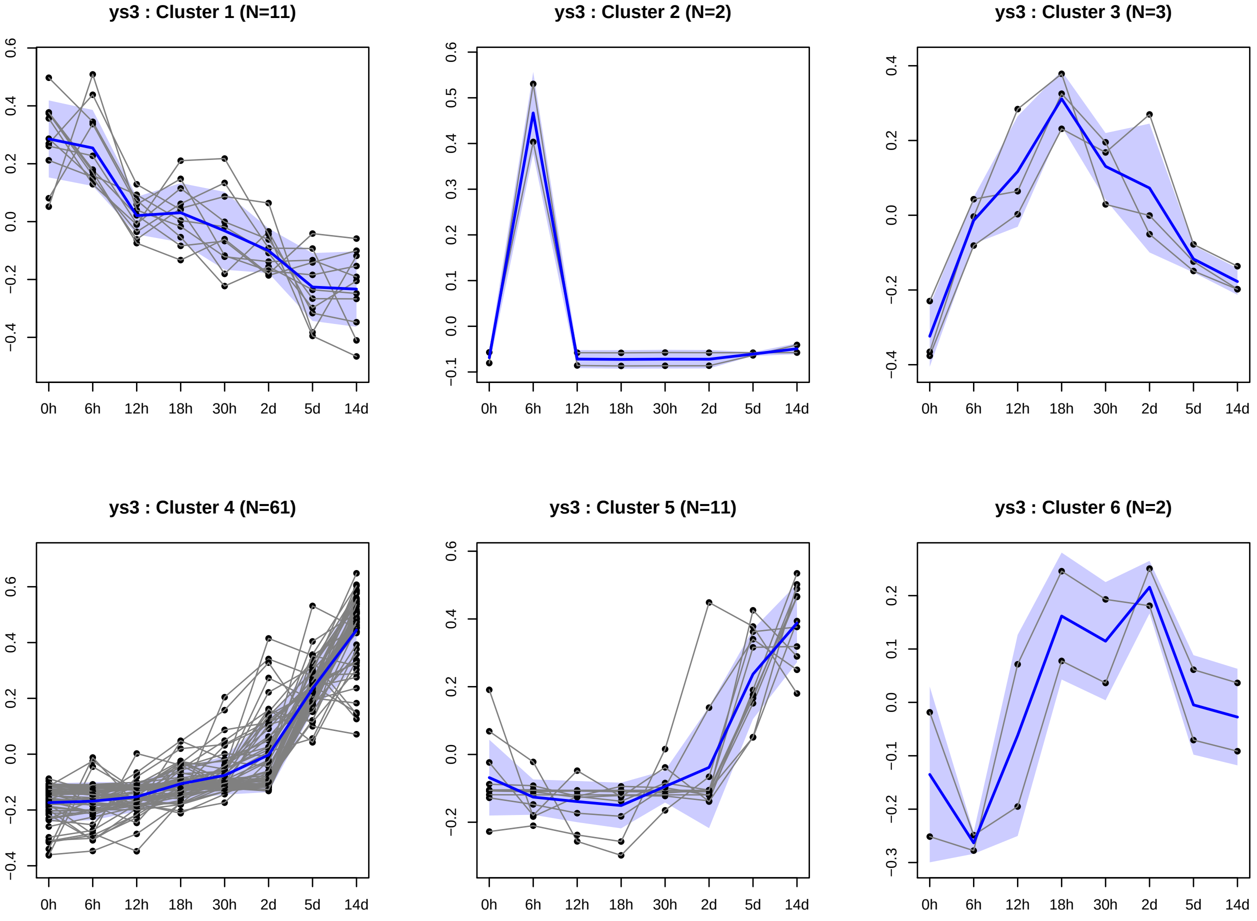

Figure: Mean cluster time courses. Mean time course of clusters in blue, with individual mean factors in cluster in grey. Standard deviation of members in cluster is added as gray shade.

In addition the plots of the individual factors in the clusters are generated. Despite being clustered together still quit large variance exists between the different factors in the clusters.

```r
# Plot individual time courses in cluster
plot_clusters_items <- function(method, toFile=FALSE){
  # get the cluster assignment for the given method
  hc.res <- hclust.res[[method]]
  groups.hc.order <- hc.res$groups.hc.order

  for (k in 1:Ngroups){
    if (toFile==TRUE){
      pdf(file.path(resultsPath, "cluster", sprintf("%s_cluster_%s.pdf", method, k)),
          width=10, height=10, pointsize=8)
    }
    g <- groups.hc.order[groups.hc.order==k]
    N <- ceiling(sqrt(length(g)))
    par(mfrow=c(N,N))
    for (name in names(g)){
      plot_single(name_A=name)
    }
    par(mfrow=c(1,1))
    if (toFile==TRUE){
      invisible(dev.off())
    }
  }
}

# plot individual factors in clusters on disk
for (method in correlation_methods){
  plot_clusters_items(method=method, toFile=TRUE)
}
rm(method)
```

Top cluster representatives

In this step, the top cluster representatives are calculated for all clusters. The higher the correlation between the factor and the cluster mean, the higher the respective factor is scored. The used correlation measure is `YS3`. The result are the correlation matrices for the individual clusters.

```r
hmap_colors <- HeatmapColors()

# correlation between the cluster mean and cluster members
calculate_cluster_correlations <- function(method="ys3"){
  if (!identical(method, "ys3")){
    stop("Only ys3 top correlation is currently supported")
  }
  # get the clusters
  hc.res <- hclust.res[[method]]
  groups.hc.order <- hc.res$groups.hc.order
  # mean cluster time course as additional factor
  name_cluster <- sprintf("cluster.mean")

  # calculate top correlations for every cluster
  cluster.cor <- vector("list", length=Ngroups)
  for (k in 1:Ngroups){
      # get members of cluster
```

```
      g <- groups.hc.order[groups.hc.order==k]
      # get normalized data for members of cluster
      dgroup <- BDLdata.norm[names(g)]
      # cluster mean and add as factor (mean of all factors in cluster
      # for given time point and sample)
      g.mean <- rowMeans(as.matrix(dgroup), na.rm=TRUE)
      dgroup[[name_cluster]] <- g.mean
      # mean averaged over repeats (for A** and M*)
      dgroup.mean <- bdl_mean_data(dgroup, BDLsamples)
      # ys3 correlation for created data.frame with mean cluster factor
      ysr.res <- ysr.matrices(dgroup.mean, BDLmean.time, use="pairwise.complete.obs")
      # Spearman correlation on individual data points
      cor.S_star <- ( cor(dgroup, method="spearman", use="pairwise.complete.obs") + 1 )/2
      # YS3 in [-1,1]
      ys3 <- 2*( (w$w1*cor.S_star + w$w2*ysr.res$A_star2 + w$w3*ysr.res$M_star) - 0.5)
      # store correlation matrix
      cluster.cor[[k]] <- ys3
  }
  return(cluster.cor)
}
cluster.cor <- calculate_cluster_correlations(method="ys3")
```

```
# plot correlations for individual clusters
for (k in 1:Ngroups){
  cluster.cor.mat <- cluster.cor[[k]]
  if (ncol(cluster.cor.mat)<20){
    corrplot(cluster.cor.mat, method="pie", type="full",
           main=sprintf("Cluster %s", k),
             tl.cex=0.8, tl.col="black", col=hmap_colors(100),
             insig="p-value", sig.level=-1, p.mat=cluster.cor.mat,
             cl.pos="n")
  } else {
    corrplot(cluster.cor.mat, method="circle", type="full",
           main=sprintf("Cluster %s", k),
             tl.cex=0.8, tl.col="black", col=hmap_colors(100),
             cl.pos="n")
  }
}
rm(k, cluster.cor.mat)
```

From the cluster correlation matrices the top correlations between cluster mean and the members of the respective cluster can be extracted.

```
plot_top_cluster_representatives <- function(method="ys3", Ntop=11, labels=TRUE){
  par(mfrow=c(Ngroups,1))
  name_cluster <- sprintf("cluster.mean")
  for (k in 1:Ngroups){
      # YS3 in [-1,1]
      ys3 <- cluster.cor[[k]]

      # correlation for the cluster mean
      v <- ys3[, which(colnames(ys3)==name_cluster)]
      # sort by absolute correlation
```

```
      v.sorted <- rev(v[order(abs(v))])

      # reduce to Ntop candidates and fill short clusters with zeros tto Ntop
      if (length(v.sorted)>=(Ntop+1)){
        v.sorted <- v.sorted[2:(Ntop+1)]
      } else {
        v.sorted <- c(v.sorted[2:length(v.sorted)], rep(0,Ntop-length(v.sorted)+1))
      }
      # prepare data for corrplot
      mv <- t(as.matrix(v.sorted))
      rownames(mv) <- c(paste(name_cluster, k))
      if (labels == FALSE){
        colnames(mv) <- NULL
      }
      corrplot(mv, method="pie", type="full",
               tl.cex=1.0, tl.col="black", col=hmap_colors(100),
               insig="p-value", sig.level=-1, p.mat=mv,
               cl.pos="n")
  }
  par(mfrow=c(1,1))
}

# plot to file (with and without names)
pdf(file.path(resultsPath, "cluster", "cluster_top_representatives_01.pdf"),
    width=5, height=5, pointsize=12)
plot_top_cluster_representatives(labels=FALSE)
invisible(dev.off())
pdf(file.path(resultsPath, "cluster", "cluster_top_representatives_02.pdf"),
    width=10, height=10, pointsize=12)
plot_top_cluster_representatives(labels=TRUE)
invisible(dev.off())
```

```
plot_top_cluster_representatives(labels=TRUE)
```

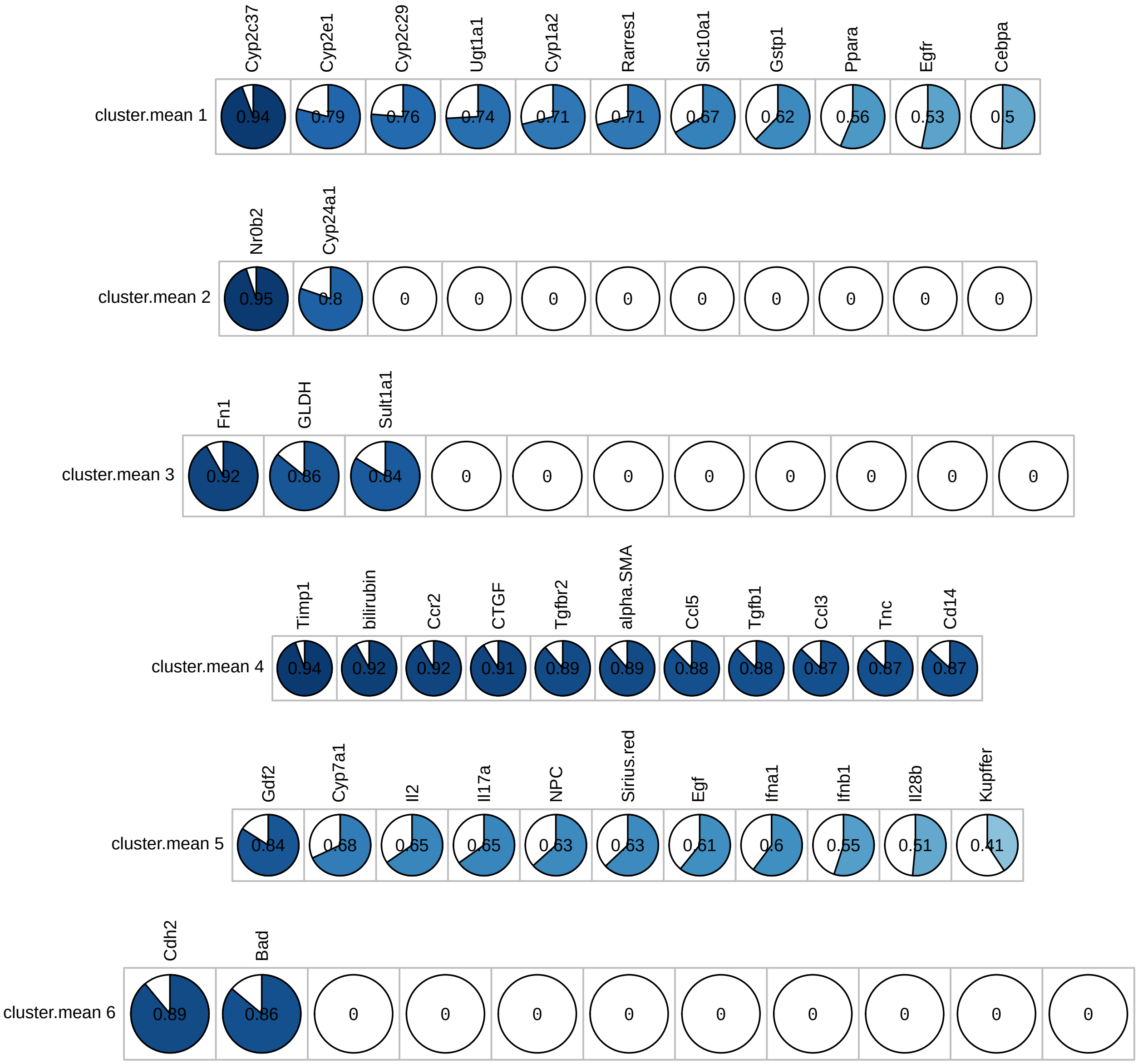

Figure: Top cluster correlations. Top correlations between the respective cluster mean and the members in the cluster sorted by absolute correlation.

YS3 cluster summary

Overview over the members of the 6 clusters with respective YS3 correlation to the cluster mean and ANOVA result.

```
# print representatives of cluster
list_cluster_information <- function(method="ys3"){
  if(!identical(method, "ys3")){
    stop("Cluster correlation only calculated for ys3")
  }
  groups <- hclust.res[[method]]$groups
  cat(sprintf("----------------------------------------\n"))
  cat(sprintf("Correlation method: *** %s ***\n", method))
```

```
  cat(sprintf("------------------------------------------\n"))
  for (k in 1:Ngroups){
    cluster.cor.mat <- cluster.cor[[k]]
    # create data.frame with ANOVA & cluster correlation
    g <- groups[groups==k]
    cat(sprintf("Cluster %s (N=%s)\n", k, length(g)))
    Ng <- length(names(g))
    rows <- vector("list", length=Ng)
    for (kn in 1:Ng){
      name <- names(g)[kn]
      row <- df.anova[which(df.anova$factor==name),]
      row$cluster.cor <- cluster.cor.mat[name, "cluster.mean"]
      rows[[kn]] <- row
    }
    df <- do.call("rbind", rows)
    df <- df[order(-df$cluster.cor), ]

    # cleanup the data.frame
    rownames(df) <- df$factors
    df <- df[, c("p.holm", "sig.holm", "ftype", "fshort", "cluster.cor")]
    df$cluster.cor <- round(df$cluster.cor, digits=2)
    df$p.holm <- sprintf("%1.2E", df$p.holm)
    print(df)

    cat("\n")
    # create list for figure legend
    # print(paste(sprintf("%s (%s)", rownames(df), df$cluster.cor),
    #             sep=", ", collapse =", " ))
  }
}
```

```
# List information in cluster
list_cluster_information(method="ys3")
```

```
------------------------------------------
Correlation method: *** ys3 ***
------------------------------------------
Cluster 1 (N=11)
          p.holm sig.holm       ftype fshort cluster.cor
Cyp2c37 1.09E-05      ***     GE_ADME               0.94
Cyp2e1  1.50E-06      ***  GE_Fibrosis              0.79
Cyp2c29 2.93E-06      ***     GE_ADME               0.76
Ugt1a1  2.75E-03       **     GE_ADME               0.74
Cyp1a2  2.93E-14      ***     GE_ADME               0.71
Rarres1 3.57E-02        *  GE_Fibrosis              0.71
Slc10a1 5.26E-05      ***     GE_ADME               0.67
Gstp1   1.38E-04      ***     GE_ADME               0.62
Ppara   1.63E-04      ***     GE_ADME               0.56
Egfr    1.59E-03       ** GE_Cytokines              0.53
Cebpa   4.93E-04      ***     GE_ADME               0.50

Cluster 2 (N=2)
          p.holm sig.holm   ftype fshort cluster.cor
Nr0b2   1.30E-07      *** GE_ADME               0.95
Cyp24a1 9.88E-03       ** GE_ADME               0.80
```

Cluster 3 (N=3)

	p.holm	sig.holm	ftype	fshort	cluster.cor
Fn1	1.21E-03	**	GE_Fibrosis		0.92
GLDH	4.28E-03	**	Biochemistry	B	0.86
Sult1a1	2.98E-03	**	GE_ADME		0.84

Cluster 4 (N=61)

	p.holm	sig.holm	ftype	fshort	cluster.cor
Timp1	4.30E-04	***	GE_Fibrosis		0.94
bilirubin	4.98E-12	***	Biochemistry	B	0.92
Ccr2	6.94E-11	***	GE_Cytokines		0.92
CTGF	7.82E-10	***	Histochemistry	H	0.91
Tgfbr2	1.71E-07	***	GE_Fibrosis		0.89
alpha.SMA	5.02E-04	***	Histochemistry	H	0.89
Ccl5	2.78E-07	***	GE_Cytokines		0.88
Tgfb1	3.30E-11	***	GE_Cytokines		0.88
Ccl3	8.53E-06	***	GE_Cytokines		0.87
Tnc	2.04E-03	**	GE_Fibrosis		0.87
Cd14	1.15E-05	***	GE_Cytokines		0.87
Ccl2	3.46E-11	***	GE_Cytokines		0.86
Cd86	6.56E-11	***	GE_Cytokines		0.86
Pdgfb	7.68E-04	***	GE_Fibrosis		0.86
Col1a1	4.40E-07	***	GE_Fibrosis		0.86
Cxcl3	6.82E-05	***	GE_Cytokines		0.86
Ccl4	1.04E-07	***	GE_Cytokines		0.85
Cxcl5	6.26E-10	***	GE_Cytokines		0.85
Il10ra	1.64E-09	***	GE_Cytokines		0.85
Col3a1	2.02E-05	***	GE_Fibrosis		0.85
Il10rb	1.15E-11	***	GE_Cytokines		0.84
Ccl7	3.40E-08	***	GE_Cytokines		0.82
Cd69	2.73E-06	***	GE_Cytokines		0.82
Ifnar1	7.71E-07	***	GE_Cytokines		0.82
Tnf	4.36E-06	***	GE_Cytokines		0.82
Osm	7.51E-06	***	GE_Cytokines		0.81
Sparc	1.07E-06	***	GE_Fibrosis		0.80
Il6	2.23E-04	***	GE_Cytokines		0.80
Tnfrsf1b	7.89E-11	***	GE_Cytokines		0.80
Cxcr2	1.75E-06	***	GE_Cytokines		0.78
Il1b	7.29E-06	***	GE_Cytokines		0.78
Timp2	6.24E-05	***	GE_Fibrosis		0.77
Ifnar2	1.82E-04	***	GE_Cytokines		0.77
Ccr5	4.36E-08	***	GE_Cytokines		0.77
Il10	4.88E-05	***	GE_Cytokines		0.76
Osmr	1.01E-07	***	GE_Cytokines		0.75
Gsta2	3.88E-06	***	GE_ADME		0.74
Il4	4.88E-05	***	GE_Cytokines		0.71
Ifng	7.51E-06	***	GE_Cytokines		0.71
Ccl8	1.23E-04	***	GE_Cytokines		0.71
Hgf	5.80E-08	***	GE_Cytokines		0.70
Bak1	2.73E-02	*	GE_Fibrosis		0.70
Mrc1	6.95E-11	***	GE_Cytokines		0.69
Tgfb2	1.08E-02	*	GE_Fibrosis		0.69
Ccr3	1.91E-06	***	GE_Cytokines		0.68
Actb.y	1.91E-02	*	GE_Cytokines		0.68
S100a4	9.19E-07	***	Histochemistry	H	0.66
Il13	9.29E-06	***	GE_Cytokines		0.66
Met	4.97E-03	**	GE_Cytokines		0.66
bile.infarcts	3.78E-02	*	Histochemistry	H	0.65

```
Il6st         8.71E-04      ***   GE_Cytokines              0.63
Tnfrsf1a      3.33E-05      ***   GE_Cytokines              0.63
Mki67         1.46E-03       **    GE_Fibrosis              0.62
Birc5         1.69E-02        *    GE_Fibrosis              0.60
Ctgf          1.32E-04      ***    GE_Fibrosis              0.58
BEC           2.09E-07      *** Histochemistry      H       0.56
Bax           2.89E-02        *    GE_Fibrosis              0.56
Notch1        4.45E-03       **    GE_Fibrosis              0.54
Cxcr1         1.07E-05      ***   GE_Cytokines              0.51
Gstm1         9.18E-09      ***        GE_ADME              0.45
Cdh1          4.48E-04      ***    GE_Fibrosis              0.42

Cluster 5 (N=11)
             p.holm sig.holm          ftype fshort cluster.cor
Gdf2       5.95E-06      ***    GE_Fibrosis              0.84
Cyp7a1     9.83E-03       **        GE_ADME              0.68
Il2        4.84E-05      ***   GE_Cytokines              0.65
Il17a      2.43E-04      ***   GE_Cytokines              0.65
NPC        5.28E-04      *** Histochemistry      H       0.63
Sirius.red 7.59E-04      *** Histochemistry      H       0.63
Egf        4.88E-05      ***   GE_Cytokines              0.61
Ifna1      1.46E-03       **   GE_Cytokines              0.60
Ifnb1      4.86E-05      ***   GE_Cytokines              0.55
Il28b      4.88E-05      ***   GE_Cytokines              0.51
Kupffer    1.95E-03       ** Histochemistry      H       0.41

Cluster 6 (N=2)
       p.holm sig.holm       ftype fshort cluster.cor
Cdh2 5.38E-04      *** GE_Fibrosis               0.89
Bad  3.84E-04      *** GE_Fibrosis               0.86
```

```
#
```

Decision trees

For the prediction of disease progression after BDL a decision tree was used. The regression tree was fitted with the R package `rpart`, being the open-source implementation of CART, providinng algorithms for recursive partitioning for classification following in most details closely Breiman et. al (1984) (*Breiman L., Friedman J. H., Olshen R. A., and Stone, C. J. (1984) Classification and Regression Trees. Wadsworth*).

The predictor variables are the 6 mean time courses of the clusters, the dependent variable is the log transformed time class (to get aprroximately equidistant intervals between the trainings classes in the regression models). The tree is built in a two-step process: First the single variable is found which best splits the data into two groups. The data than separated based on the split, and the splitting process is applied separately to each sub-group, and so on recursively until the subgroups either reach a minimum size (`minbucket`) or until no improvement can be made. The second stage of the procedure consists of using cross-validation to trim back the full tree. The splitting criterion, which is used to decide which variable gives the best split for nodes in the regression trees is $SS_T - (SS_L + SS_R)$, with $SS_T = \sum (y_i - < y >)^2$ the sum of squares for the node and SS_R and SS_L the sums of squares for the left and right son. This is equivalent to choosing the split ot maximize the between-groups sum-of-squares in a simple analysis of variance (see *An Introduction to Recursive Partitioning Using the RPART Routines. T.M. Therneau and E.J. Atkinson, Mayo Foundation, 2015*). Two important parameters controlling the resulting tree are

- `minsplit` : The minimum number of observations in a node for which the routine will even try to compute a split. The default is 20 and is set to 6 in the tree calculation ($N_r = 5$ repeats per time point).

- `minbucket` : The minimum number of observations in a terminal node. This defaults to minsplit/3.

```
suppressPackageStartupMessages(library(rpart))
suppressPackageStartupMessages(library(rpart.plot))
suppressPackageStartupMessages(library(caret))
dir.create(file.path(resultsPath, 'decision_tree'), showWarnings=FALSE)
```

To generate approximatly equally distant time classes for training the regression tree the log transformed time values are used based on the transformation

$$\tilde{t_i} = log(t_i + 1)$$

resulting in the following transformation of the time points

```
# transform data to log scale
log_transform <- function(data){
  log(data+1)
}
# back transformation
log_transform_back <- function(log_data){
  exp(log_data)-1
}
# resulting time transformations
time_transformation <- data.frame(time=BDLmean.time,
                                  log_time=log_transform(BDLmean.time))
print(round(time_transformation, 2))
```

```
  time log_time
1    0     0.00
2    6     1.95
3   12     2.56
4   18     2.94
5   30     3.43
6   48     3.89
7  120     4.80
8  336     5.82
```

Trainings data

In a first step the mean cluster trainings data for fitting the decision tree is prepared. The predictor variables are the mean samples of the clusters, the dependent variable is the log transformed time of the respective sample. The trainings set consists of the $N_t * N_r = 40$ samples.

```
  prepare_treedata_mean <- function(method="ys3"){
  # Hierarchical clusters based on ys3 to fit the regression tree
  hc.res <- hclust.res[[method]]
  groups <- hc.res$groups

  # Prepare training set for fitting the decision trees (mean cluster data set,
  # i.e. mean over normalized factors in cluster).
  na.vec <- rep(NA, Nt*Nr)
  treedata.mean <- data.frame(c1=na.vec, c2=na.vec, c3=na.vec,
```

```
                                c4=na.vec, c5=na.vec, c6=na.vec)
  # for every sample
  for (ks in 1:(Nt*Nr)){
    # create the mean over the cluster
    for (kgroup in 1:Ngroups){
      # get factors in the cluster
      factors <- names(groups[groups==kgroup])
      # calculate mean over normalized data
      treedata.mean[ks, kgroup] <- mean(as.numeric(BDLdata.norm[ks, factors]), na.rm=TRUE)
    }
  }
  # add log transformed time [h] for regression
  treedata.mean$logtime <- log_transform(BDLsamples$time)
  # add experimental time class
  treedata.mean$class <- BDLsamples$time_fac
  return(treedata.mean)
}
treedata.mean <- prepare_treedata_mean()

# save the trainings data
save(treedata.mean, file=file.path(resultsPath, "data", "treedata.mean.Rdata"))
```

In the following an overview over the resulting trainings data set is given

```
# mean cluster data set for model fitting
options(width=200)
print(round(treedata.mean[, -8], digits=2))
```

```
      c1    c2    c3    c4    c5    c6 logtime
1   0.22 -0.07 -0.36 -0.14 -0.11 -0.03    0.00
2   0.50 -0.06 -0.29 -0.14 -0.07 -0.07    0.00
3   0.26 -0.07 -0.32 -0.19 -0.10 -0.17    0.00
4   0.34 -0.07 -0.31 -0.19 -0.04 -0.23    0.00
5   0.12 -0.07 -0.33 -0.19 -0.02 -0.18    0.00
6   0.31  0.69 -0.01 -0.17 -0.09 -0.29    1.95
7   0.21  0.93 -0.02 -0.18 -0.13 -0.36    1.95
8   0.24  0.27  0.03 -0.18 -0.15 -0.30    1.95
9   0.19  0.12  0.05 -0.15 -0.14 -0.25    1.95
10  0.33  0.33 -0.12 -0.14 -0.11 -0.12    1.95
11  0.01 -0.07  0.23 -0.13 -0.15 -0.01    2.56
12  0.08 -0.07  0.01 -0.15 -0.11 -0.07    2.56
13 -0.07 -0.07  0.12 -0.17 -0.15 -0.18    2.56
14  0.07 -0.07  0.09 -0.15 -0.14  0.03    2.56
15  0.01 -0.07  0.13 -0.15 -0.15 -0.08    2.56
16  0.13 -0.07  0.30 -0.05 -0.16  0.17    2.94
17 -0.03 -0.07  0.39 -0.12 -0.15  0.24    2.94
18  0.02 -0.07  0.22 -0.13 -0.15  0.07    2.94
19  0.02 -0.07  0.43 -0.07 -0.15  0.20    2.94
20  0.01 -0.07  0.22 -0.15 -0.14  0.12    2.94
21  0.05 -0.07 -0.01 -0.09 -0.04  0.14    3.43
22 -0.02 -0.07 -0.03 -0.07 -0.10  0.08    3.43
23 -0.11 -0.07  0.41 -0.11 -0.16  0.03    3.43
24 -0.11 -0.07  0.13 -0.03 -0.12  0.09    3.43
25  0.03 -0.07  0.17 -0.08 -0.05  0.23    3.43
26 -0.13 -0.07  0.01  0.02 -0.04  0.11    3.89
27 -0.17 -0.07  0.26  0.13  0.00  0.43    3.89
```

```
28 -0.09 -0.07 -0.15  0.00 -0.04  0.28    3.89
29 -0.08 -0.07  0.12 -0.11 -0.14  0.15    3.89
30 -0.03 -0.07  0.12 -0.08 -0.02  0.12    3.89
31 -0.23 -0.07 -0.27 -0.01  0.14 -0.09    4.80
32 -0.26 -0.07  0.11  0.27  0.17  0.27    4.80
33 -0.28 -0.07 -0.27  0.29  0.19 -0.13    4.80
34 -0.26 -0.07 -0.23  0.28  0.23 -0.13    4.80
35 -0.10 -0.01  0.07  0.33  0.44  0.06    4.80
36 -0.16  0.03 -0.17  0.45  0.44 -0.06    5.82
37 -0.22 -0.06 -0.14  0.64  0.49 -0.01    5.82
38 -0.23 -0.07 -0.13  0.23  0.26 -0.07    5.82
39 -0.24 -0.07 -0.17  0.54  0.44  0.12    5.82
40 -0.32 -0.07 -0.27  0.36  0.30 -0.11    5.82
```

```
options(width=75)
```

Fit regression tree

Here the regression tree is fitted (log transformed time ~ mean cluster data for the time points c1, c2, c3, c4, c5, c6). Control parameters for rpartare `minsplit=6` and `minbucket=2` to account for the low number of samples (`N=40`). In the following the regression tree is fitted and the information for the resulting tree is provided.

```
# formula for regression tree
formula.reg = paste("logtime ~ c1 + c2 + c3 + c4 + c5 + c6")

# fit regression tree with mean cluster data
tree.reg <- rpart(formula=formula.reg,
                  data=treedata.mean,
                  method="anova",
                  control=rpart.control(minsplit=6, minbucket=2, cp=0.01))

# pretty plot of tree to file
pdf(file.path(resultsPath, 'decision_tree', "regression_tree.pdf"),
    width=10, height=5, pointsize=12)
prp(tree.reg, type=0, extra=101, yesno=TRUE)
invisible(dev.off())
```

```
# plot of regression tree
prp(tree.reg, type=0, extra=101, yesno=TRUE)
```

```
# visualize cross-validation results
# rsq.rpart(tree.reg)
```

Figure: Regression tree on mean clusters. Fitted regression tree on the mean cluster data, predicting the log time classes. Splitting points are on the mean cluster values.

Detailed information about the fitted tree is provided below

```
# detailed overview of the resulting tree
options(width=200)
print(tree.reg)
```

```
n= 40
```

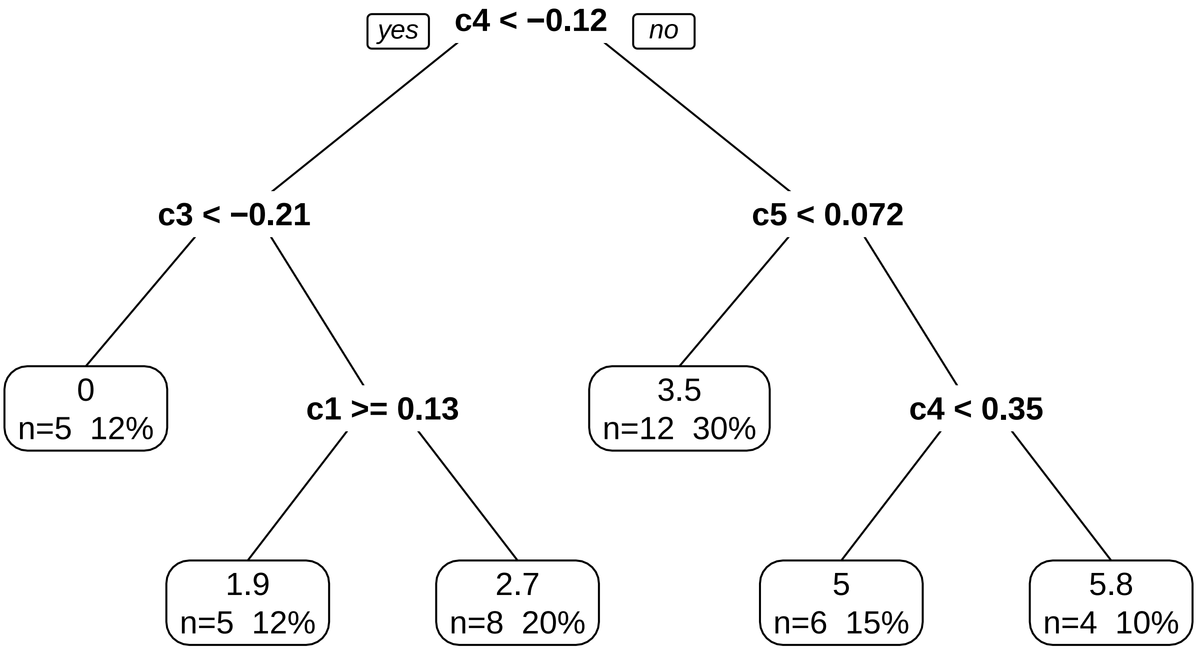

Figure 1: Figure: Regression tree on mean clusters. Fitted regression tree on the mean cluster data, predicting the log time classes. Splitting points are on the mean cluster values.

```
node), split, n, deviance, yval
      * denotes terminal node

 1) root 40 1.111047e+02 3.174622
   2) c4< -0.1174939 18 2.310447e+01 1.743756
     4) c3< -0.2081362 5 0.000000e+00 0.000000 *
     5) c3>=-0.2081362 13 2.053563e+00 2.414432
      10) c1>=0.1331057 5 2.465190e-31 1.945910 *
      11) c1< 0.1331057 8 2.700232e-01 2.707258 *
   3) c4>=-0.1174939 22 2.099513e+01 4.345331
     6) c5< 0.07192204 12 1.384347e+00 3.543160 *
     7) c5>=0.07192204 10 2.622937e+00 5.307937
      14) c4< 0.3463259 6 8.743124e-01 4.966506 *
      15) c4>=0.3463259 4 0.000000e+00 5.820083 *
```

```r
summary(tree.reg)
```

```
Call:
rpart(formula = formula.reg, data = treedata.mean, method = "anova",
    control = rpart.control(minsplit = 6, minbucket = 2, cp = 0.01))
  n= 40

          CP nsplit  rel error     xerror        xstd
1 0.60308067      0 1.00000000 1.02423310 0.205452411
2 0.18946910      1 0.39691933 0.43756577 0.062438655
3 0.15289945      2 0.20745023 0.26253022 0.051583089
4 0.01605279      3 0.05455078 0.06328074 0.008363276
5 0.01573853      4 0.03849799 0.07744295 0.013686407
6 0.01000000      5 0.02275946 0.04970344 0.013909474

Variable importance
c4 c5 c1 c3 c6 c2
27 19 19 15 12  8

Node number 1: 40 observations,    complexity param=0.6030807
  mean=3.174622, MSE=2.777617
  left son=2 (18 obs) right son=3 (22 obs)
  Primary splits:
      c4 < -0.1174939   to the left,  improve=0.6030807, (0 missing)
      c1 < -0.07537026  to the right, improve=0.5729499, (0 missing)
      c5 < -0.008508656 to the left,  improve=0.5487412, (0 missing)
      c3 < -0.2829117   to the left,  improve=0.5183388, (0 missing)
      c6 < -0.149531    to the left,  improve=0.3184694, (0 missing)
  Surrogate splits:
      c1 < -0.07537026  to the right, agree=0.850, adj=0.667, (0 split)
      c5 < -0.0625688   to the left,  agree=0.800, adj=0.556, (0 split)
      c6 < -0.149531    to the left,  agree=0.750, adj=0.444, (0 split)
      c2 < 0.07279386   to the right, agree=0.675, adj=0.278, (0 split)
      c3 < -0.2829117   to the left,  agree=0.675, adj=0.278, (0 split)

Node number 2: 18 observations,    complexity param=0.1894691
  mean=1.743756, MSE=1.283582
  left son=4 (5 obs) right son=5 (13 obs)
  Primary splits:
      c3 < -0.2081362   to the left,  improve=0.9111184, (0 missing)
```

```
      c5 < -0.1122942   to the right, improve=0.5991729, (0 missing)
      c1 < 0.09730379   to the right, improve=0.5785906, (0 missing)
      c4 < -0.1838753   to the left,  improve=0.4737815, (0 missing)
      c6 < -0.01908181  to the left,  improve=0.3296556, (0 missing)
  Surrogate splits:
      c4 < -0.1838753   to the left,  agree=0.889, adj=0.6, (0 split)
      c5 < -0.1122942   to the right, agree=0.889, adj=0.6, (0 split)
      c1 < 0.3351088    to the right, agree=0.833, adj=0.4, (0 split)

Node number 3: 22 observations,    complexity param=0.1528994
  mean=4.345331, MSE=0.9543241
  left son=6 (12 obs) right son=7 (10 obs)
  Primary splits:
      c5 < 0.07192204   to the left,  improve=0.8091327, (0 missing)
      c4 < 0.1840943    to the left,  improve=0.7540271, (0 missing)
      c1 < -0.1437557   to the right, improve=0.6642991, (0 missing)
      c3 < -0.08194432  to the right, improve=0.6127738, (0 missing)
      c6 < 0.007982308  to the right, improve=0.5246106, (0 missing)
  Surrogate splits:
      c4 < 0.1840943    to the left,  agree=0.955, adj=0.9, (0 split)
      c1 < -0.1936876   to the right, agree=0.909, adj=0.8, (0 split)
      c3 < -0.08194432  to the right, agree=0.864, adj=0.7, (0 split)
      c6 < 0.007982308  to the right, agree=0.864, adj=0.7, (0 split)
      c2 < -0.07256249  to the right, agree=0.727, adj=0.4, (0 split)

Node number 4: 5 observations
  mean=0, MSE=0

Node number 5: 13 observations,    complexity param=0.01605279
  mean=2.414432, MSE=0.1579664
  left son=10 (5 obs) right son=11 (8 obs)
  Primary splits:
      c1 < 0.1331057    to the right, improve=0.8685099, (0 missing)
      c2 < 0.02364916   to the right, improve=0.8685099, (0 missing)
      c3 < 0.06868251   to the left,  improve=0.7242745, (0 missing)
      c6 < -0.09746977  to the left,  improve=0.7242745, (0 missing)
      c5 < -0.1383548   to the right, improve=0.4701461, (0 missing)
  Surrogate splits:
      c2 < 0.02364916   to the right, agree=1.000, adj=1.0, (0 split)
      c3 < 0.06868251   to the left,  agree=0.923, adj=0.8, (0 split)
      c6 < -0.2167742   to the left,  agree=0.923, adj=0.8, (0 split)
      c4 < -0.153395    to the left,  agree=0.846, adj=0.6, (0 split)
      c5 < -0.1383548   to the right, agree=0.846, adj=0.6, (0 split)

Node number 6: 12 observations
  mean=3.54316, MSE=0.1153623

Node number 7: 10 observations,    complexity param=0.01573853
  mean=5.307937, MSE=0.2622937
  left son=14 (6 obs) right son=15 (4 obs)
  Primary splits:
      c4 < 0.3463259    to the left,  improve=0.6666667, (0 missing)
      c5 < 0.2491244    to the left,  improve=0.6666667, (0 missing)
      c6 < -0.1217972   to the left,  improve=0.2500000, (0 missing)
      c3 < -0.0328633   to the right, improve=0.2500000, (0 missing)
      c1 < -0.247735    to the left,  improve=0.1666667, (0 missing)
  Surrogate splits:
      c5 < 0.2821139    to the left,  agree=0.9, adj=0.75, (0 split)
      c1 < -0.2249667   to the left,  agree=0.7, adj=0.25, (0 split)
      c2 < -0.06371928  to the left,  agree=0.7, adj=0.25, (0 split)
      c3 < -0.1376999   to the right, agree=0.7, adj=0.25, (0 split)
      c6 < -0.06444851  to the left,  agree=0.7, adj=0.25, (0 split)

Node number 10: 5 observations
  mean=1.94591, MSE=4.930381e-32

Node number 11: 8 observations
  mean=2.707258, MSE=0.0337529

Node number 14: 6 observations
  mean=4.966506, MSE=0.1457187

Node number 15: 4 observations
  mean=5.820083, MSE=0
```

```r
printcp(tree.reg)
```

```
Regression tree:
rpart(formula = formula.reg, data = treedata.mean, method = "anova",
    control = rpart.control(minsplit = 6, minbucket = 2, cp = 0.01))

Variables actually used in tree construction:
[1] c1 c3 c4 c5

Root node error: 111.1/40 = 2.7776

n= 40

        CP nsplit rel error   xerror      xstd
1 0.603081      0  1.000000 1.024233 0.2054524
2 0.189469      1  0.396919 0.437566 0.0624387
3 0.152899      2  0.207450 0.262530 0.0515831
4 0.016053      3  0.054551 0.063281 0.0083633
5 0.015739      4  0.038498 0.077443 0.0136864
6 0.010000      5  0.022759 0.049703 0.0139095
```

```r
tree.reg$frame
```

```
      var  n wt          dev     yval  complexity ncompete nsurrogate
1      c4 40 40 1.111047e+02 3.174622 0.603080672        4          5
```

```
2       c3 18 18 2.310447e+01 1.743756 0.189469098        4         3
4  <leaf>  5  5 0.000000e+00 0.000000 0.010000000        0         0
5       c1 13 13 2.053563e+00 2.414432 0.016052788        4         5
10 <leaf>  5  5 2.465190e-31 1.945910 0.010000000        0         0
11 <leaf>  8  8 2.700232e-01 2.707258 0.010000000        0         0
3       c5 22 22 2.099513e+01 4.345331 0.152899447        4         5
6  <leaf> 12 12 1.384347e+00 3.543160 0.007176239        0         0
7       c4 10 10 2.622937e+00 5.307937 0.015738533        4         5
14 <leaf>  6  6 8.743124e-01 4.966506 0.010000000        0         0
15 <leaf>  4  4 0.000000e+00 5.820083 0.010000000        0         0
```

```r
options(width=75)
```

Only a subset of all mean clusters is used in the regression tree:

```r
# variables used for splitting in the decision tree
tree.nodes <- (tree.reg$frame)$var
tree.nodes <- tree.nodes[tree.nodes != "<leaf>"]
tree.vars <- as.character(sort(unique(tree.nodes)))
rm(tree.nodes)
# variables used for splitting decisions in tree
print(tree.vars)
```

```
[1] "c1" "c3" "c4" "c5"
```

Leave-one-out cross validation

A leave-one-out approach was used to test the robustness of the predicted time classes and predictive performance: For each sample ($N_s = 40$ mice), the regression tree was generated under the exclusion of data from the sample, with subsequent prediction on the left out test data

```r
# in total 40 cross validations (individual mice are not followed through time)
trees.test <- vector("list", length=Nr*Nt)  # fitted trees
pred.test.log <- rep(NA, length=Nr*Nt)
for (k in 1:(Nr*Nt)){
  # delete the k index
  idx.subset <- 1:(Nr*Nt)
  idx.subset <- idx.subset[-k]

  # fit tree with the subset
  t.test <- rpart(formula=formula.reg,
                  data=treedata.mean[idx.subset, ],
                  method="anova",
                  control=rpart.control(minsplit=6, minbucket=2, cp=0.01))
  trees.test[[k]] <- t.test

  # prediction on left out sample
  pred.test.log[k] <- predict(t.test, newdata=treedata.mean[k,], type="vector")
}
# transformation to time in [h]
pred.test <- log_transform_back( pred.test.log )

plot_cross_validation <- function(){
# plot the log prediction and error
  par(mfrow=c(1,2))
  plot(log_transform(BDLsamples$time), log_transform(pred.test),
       main="Leave one out cross validation",
```

```
        xlim=c(0,7), ylim=c(0,7),
        xlab="log(time_exp)",
        ylab="log(time_pre)")
  abline(a=0, b=1, col="gray")
  textxy(log_transform(BDLsamples$time), log_transform(pred.test),
          1:(Nr*Nt), col="black", cex=0.6)

  plot(log_transform(BDLsamples$time),
        log_transform(BDLsamples$time)-log_transform(pred.test),
        main="Prediction error",
        xlab="log(time_exp)",
        ylab="log(time_exp)-log(time_pre)",
        xlim=c(0,7), ylim=c(-1.5, 1.5))
  abline(a=0, b=0, col="gray")
  textxy(log_transform(BDLsamples$time),
          log_transform(BDLsamples$time)-log_transform(pred.test),
          1:(Nr*Nt), col="black", cex=0.6)
  par(mfrow=c(1,1))
}
```

```
plot_cross_validation()
```

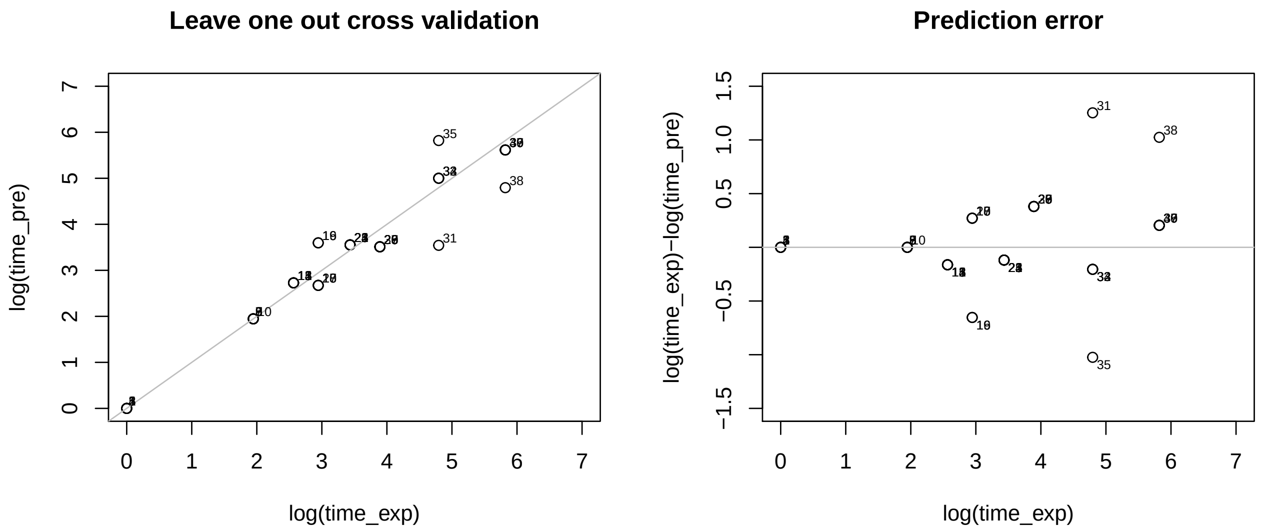

Figure: Cross validation predictions. Predictions on the left out data set with the fitted tree.

Results: All time points of the left-out data are predicted close to their actual time.

Prediction on trainings data

Predict data with the tree: Here the time class leaves based on the mean cluster data. The regression tree predicts log classes, which are back-transformed to time in [h]. Here we test how good the tree performing on the trainings data set, i.e. the mean cluster data. Predictons are evaluated based on the distance between the predicted and the experimental time classes based on the following distance measure on log scale

```
# L2 (euclidian) distance measurement on the log transformed data.
# Analog to the distance measurement in fitting the regression tree.
log_distance <- function(d1, d2){
```

```
  # sums over all the distances of the samples in log space
  log_rmsd <- sqrt(sum( (log_transform(d1)-log_transform(d2) )^2 ))/length(d1)
  return(log_rmsd)
}
```

Prediction on trainings data

```
# mean cluster predictions
pred.mean.log <- predict(tree.reg, newdata=treedata.mean, type="vector")
# transformation to time in [h]
pred.mean <- log_transform_back( pred.mean.log )

# Distance calculation (predicted to experimental)
dist.mean.all <- treedata.mean$logtime - pred.mean.log
dist.mean <- log_distance(pred.mean, BDLsamples$time)

# plot predicted ~ experimentell
plot_mean_prediction <- function(){
  par(mfrow=c(1,2))
  plot(BDLsamples$time, pred.mean, pch=15, col=rgb(0,0,1, 0.2),
       main="Regression Tree:\nPredicted ~ experimentell time",
       xlab="experimentell time [h]", ylab="predicted time [h]")
  abline(a=0, b=1, col=rgb(0.5,0.5,0.5,0.5))
  hist(dist.mean.all, breaks=seq(from=-1.05, to=1.05, by=0.1),
       main="Histogram prediction error:\nmean cluster data",
       col=rgb(0.5,0.5,0.5,0.5),
       xlab="logtime(exp)-logtime(pred)")
  par(mfrow=c(1,1))
}
# plot to file
pdf(file.path(resultsPath, 'decision_tree', "prediction_mean.pdf"),
    width=10, height=5, pointsize=12)
plot_mean_prediction()
invisible(dev.off())
```

```
plot_mean_prediction()
```

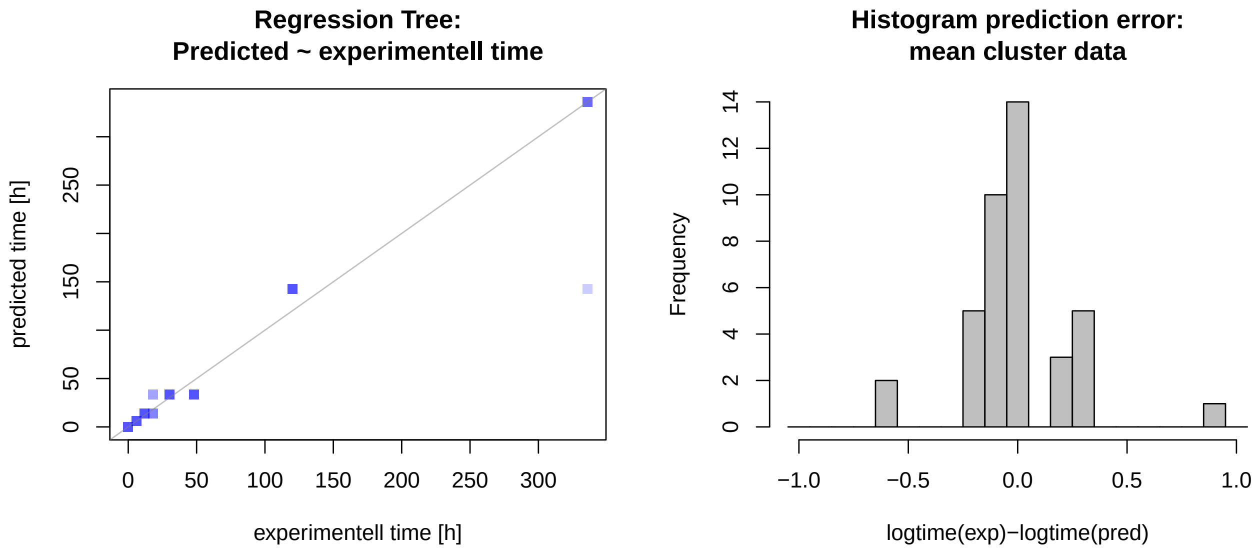

Figure: Predictions on mean cluster data. Predictions using the mean cluster data. This is the trainings data and results in the best performing tree.

The ranges of the predicted classes by the regression trees can be calculated based on the split points on log scale. These provides the information which time points would be classified in which class in the regression tree.

```
calculate_node_ranges <- function(){
  # classes via predicted classes for cluster data
  node_levels <- levels(as.factor(pred.mean))
  # These are the predicted classes
  node_classes <- round(as.numeric(node_levels), digits=1)

  # get the intervals of the time classes
  node_mean <- as.numeric(levels(as.factor(pred.mean.log)))
  node_midpoints <- (node_mean[2:length(node_mean)]+node_mean[1:(length(node_mean)-1)])/2
  # minimum of range
  node_min <- node_mean
  node_min[2:length(node_min)] <- node_midpoints
  # maximum of range
  node_max <- node_mean
  node_max[1:(length(node_min)-1)] <- node_midpoints
  # ranges in log scale
  node_ranges.log <- data.frame(node_mean, node_min, node_max)

  node_ranges <- data.frame(mean=log_transform_back(node_mean),
                            min=log_transform_back(node_min),
                            max=log_transform_back(node_max))
  rownames(node_ranges) <- paste("class", 1:nrow(node_ranges))
  return(node_ranges)
}

# predicted time classes by decision tree
node_ranges <- calculate_node_ranges()
print(round(node_ranges, digits=1))
```

```
         mean   min   max
class 1   0.0   0.0   1.6
class 2   6.0   1.6   9.2
class 3  14.0   9.2  21.8
class 4  33.6  21.8  69.4
class 5 142.5  69.4 218.9
class 6 336.0 218.9 336.0
```

Test data for evaluation

Single factor per cluster

Now the data set consisting of all single factor combinations from the clusters is created. These are used to evaluate the fitted regression tree.

```
# names of factors in the clusters
cluster_names <- paste('c', 1:Ngroups, sep="")
```

```r
cluster.factors <- vector("list", length=Ngroups)
groups <- (hclust.res$ys3)$groups  # get the ys3 groups
for (k in 1:Ngroups){
  cluster.factors[[k]] <- as.character(names(groups[groups==k]))
}
names(cluster.factors) <- cluster_names

# create data.frame of all single combinations from clusters
single.combinations <- expand.grid(cluster.factors, stringsAsFactors=TRUE)
names(single.combinations) <- names(cluster.factors)
# number of single combinations
Nsingle <- nrow(single.combinations)

# create all single factor data
print("Calculating single factor data (~ 3min) ... ")
```

```
[1] "Calculating single factor data (~ 3min) ... "
```

```r
ptm <- proc.time()
treedata.single <- vector("list", Nsingle)  # list for all combinations
for (k in 1:Nsingle){
  # ---------------------------------------------------------------
  # THIS HAS TO BE FAST (<0.005 s)
  # ---------------------------------------------------------------
  # ptm <- proc.time() # Start the clock!
  # get factor data
  tmp <- BDLdata.norm[, t(single.combinations[k, ]) ]
  # add regression values
  tmp[c("class", "logtime")] <- treedata.mean[ c("class", "logtime")]
  # add factor fields
  tmp[, paste(cluster_names, '.id', sep="")] <- single.combinations[k,]
  colnames(tmp) <- c(cluster_names, 'class', 'logtime', paste(cluster_names, '.id', sep=""))
  # store data
  treedata.single[[k]] <- tmp
  # if (k%%500 == 0){print(k)}
  # print(proc.time()-ptm)  # Stop the clock
}
# Stop the clock
rm(tmp,k)
print(proc.time() - ptm)
```

```
   user  system elapsed
121.133   0.132 121.277
```

```r
# which factor combinations only use genes
factor_is_gene <- BDLfactors$ftype %in% c("GE_ADME", "GE_Cytokines", "GE_Fibrosis")
names(factor_is_gene) <- BDLfactors$id
# vector for lookup if only genes were used
gene_only.single <- vector("logical", Nsingle)
for (k in 1:Nsingle){
  gene_only.single[k] <- all(factor_is_gene[t(single.combinations[k,])])
}
rm(k)
```

The single factor data was created consisting of 88572 combinations.

Double factor per cluster

Create a sample of double combinations from the various clusters.

```
print("Calculating double factor data (~ 1min) ... ")
```

```
[1] "Calculating double factor data (~ 1min) ... "
```

```
ptm <- proc.time()
set.seed(123456)
Ndouble <- 10000
treedata.double <- vector("list", Ndouble)  # list for sampled double combinations
for (k in 1:Ndouble){
  # sample from the 4 clusters without replacement
  n1 = sample(cluster.factors[[1]], 2, replace=FALSE)
  n2 = sample(cluster.factors[[2]], 2, replace=FALSE)
  n3 = sample(cluster.factors[[3]], 2, replace=FALSE)
  n4 = sample(cluster.factors[[4]], 2, replace=FALSE)
  n5 = sample(cluster.factors[[5]], 2, replace=FALSE)
  n6 = sample(cluster.factors[[6]], 2, replace=FALSE)
  # The mean of the combination is used (handle NAs)
  tmp <- 0.5 * (  BDLdata.norm[, c(n1[1], n2[1], n3[1], n4[1], n5[1], n6[1])]
              + BDLdata.norm[, c(n1[2], n2[2], n3[2], n4[2], n5[2], n6[2])] )
   # add class and regression values
  tmp[c("class", "logtime")] <- treedata.mean[ c("class", "logtime")]
  # add factor fields
  tmp[ , paste(cluster_names, '.id', sep="")] <- data.frame(paste(n1, collapse="__"),
                                                       paste(n2, collapse="__"),
                                                       paste(n3, collapse="__"),
                                                       paste(n4, collapse="__"),
                                                       paste(n5, collapse="__"),
                                                       paste(n6, collapse="__"))
  colnames(tmp) <- c(cluster_names, 'class', 'regvalue',
                     paste(cluster_names, '.id', sep=""))
  # store data
  treedata.double[[k]] <- tmp
}
rm(k, tmp)
print(proc.time() - ptm)
```

```
   user  system elapsed
 36.120   0.060  36.345
```

The double factor data was created consisting of a random selection of 10^{4} combinations.

Prediction on test data

Single representative predictions

Time class prediction with regression tree for single representative from each cluster

```
print("Predicting single factor data (~ 2min) ... ")
```

```
[1] "Predicting single factor data (~ 2min) ... "
```

```
pred.single.all <- vector("list", length(treedata.single))
for (k in (1:length(treedata.single))){
  # prediction and back transformation
  pred.single.all[[k]] <- log_transform_back( predict(tree.reg, newdata=treedata.single[[k]], method="a
}
pred.single <- do.call("rbind", pred.single.all)

# distance for all predictions on single factor per cluster
dist.single <- rep(NA, Nsingle)
for (k in 1:Nsingle){
  dist.single[k] <- log_distance(pred.single[k,], BDLsamples$time)
}
```

Double representative predictions

Time class prediction with regression tree for random selection of two representatives from each clusters

```
print("Predicting double factor data (~ 1min) ... ")
```

```
[1] "Predicting double factor data (~ 1min) ... "
```

```
pred.double.all <- vector("list", length(treedata.double))
for (k in (1:length(treedata.double))){
  pred.double.all[[k]] <- log_transform_back( predict(tree.reg, newdata=treedata.double[[k]], method="a
}
pred.double <- do.call("rbind", pred.double.all)

# distance for predictions on 2 sampled factors per cluster
dist.double <- rep(NA, Ndouble)
for (k in 1:Ndouble){
  dist.double[k] <- log_distance(pred.double[k,], BDLsamples$time)
}
```

Best factor combinations

Finding the best regression trees based on i) all single representatives from the clusters; and ii) single representatives only consisting of gene probes. The best is defined by minimal euclidian distance between experimental classes and predicted classes on the log scale. The best tree is not refitted with the respective factors in the tree, but the mean cluster tree uses the respective factor data for prediction.

```
# Best decision tree using all factors (minimal distance)
dist.rep.best <- min(dist.single)
# best combination of representatives for the clusters (remove duplicates)
rep.best <- unique(single.combinations[which(dist.single==dist.rep.best),
                                       c("c1", "c3", "c4", "c5")])
rep.best.idx <- rownames(rep.best)[1]
```

```r
# predictions of best representative
pred.rep.best <- pred.single[as.numeric(rep.best.idx), ]

print("Best single representatives for decision tree:")
```

```
[1] "Best single representatives for decision tree:"
```

```r
print(rep.best)
```

```
          c1   c3     c4    c5
16062 Cyp1a2  Fn1 S100a4 Il17a
16084 Cyp1a2 GLDH S100a4 Il17a
```

```r
print(dist.rep.best)
```

```
[1] 0.05554854
```

```r
# ------------------------
# Best decision tree using only gene factors, i.e. first reduce to the gene combinations
dist.single.genes <- dist.single[gene_only.single]
single.combinations.genes <- single.combinations[gene_only.single,]
dist.gene.best <- min(dist.single.genes)
# find best gene combination
gene.best <- unique(single.combinations.genes[which(dist.single.genes==dist.gene.best),
                                              c("c1", "c3", "c4", "c5")])
# predictions with best representative
gene.best.idx <- rownames(gene.best)
pred.gene.best <- pred.single[as.numeric(gene.best.idx), ]

print("Best single representatives based on genes for decision tree:")
```

```
[1] "Best single representatives based on genes for decision tree:"
```

```r
print(gene.best)
```

```
          c1  c3     c4    c5
14808 Cyp1a2 Fn1 Col1a1 Il17a
```

```r
print(dist.gene.best)
```

```
[1] 0.06672908
```

```r
# ------------------------
# Plot time courses of the representatives/factors in provided tree combinations
plot_tree_representatives <- function(combination){
  Nc <- ncol(combination)
  Nr <- nrow(combination)
  par(mfrow=c(Nr, Nc))
  for (kr in 1:Nr){
```

```r
    for (kc in 1:Nc){
      cluster <- colnames(combination)[kc]
      name <- as.character(combination[kr, kc])
      plot_single(name)
    }
  }
  par(mfrow=c(1,1))
}

# plot to file
pdf(file.path(resultsPath, "decision_tree", "rep.best.representatives.pdf"),
    width=10, height=6, pointsize=12)
plot_tree_representatives(rep.best)
invisible(dev.off())
pdf(file.path(resultsPath, "decision_tree", "gene.best.representatives.pdf"),
    width=10, height=3, pointsize=12)
plot_tree_representatives(gene.best)
invisible(dev.off())
```

```r
plot_tree_representatives(rep.best)
```

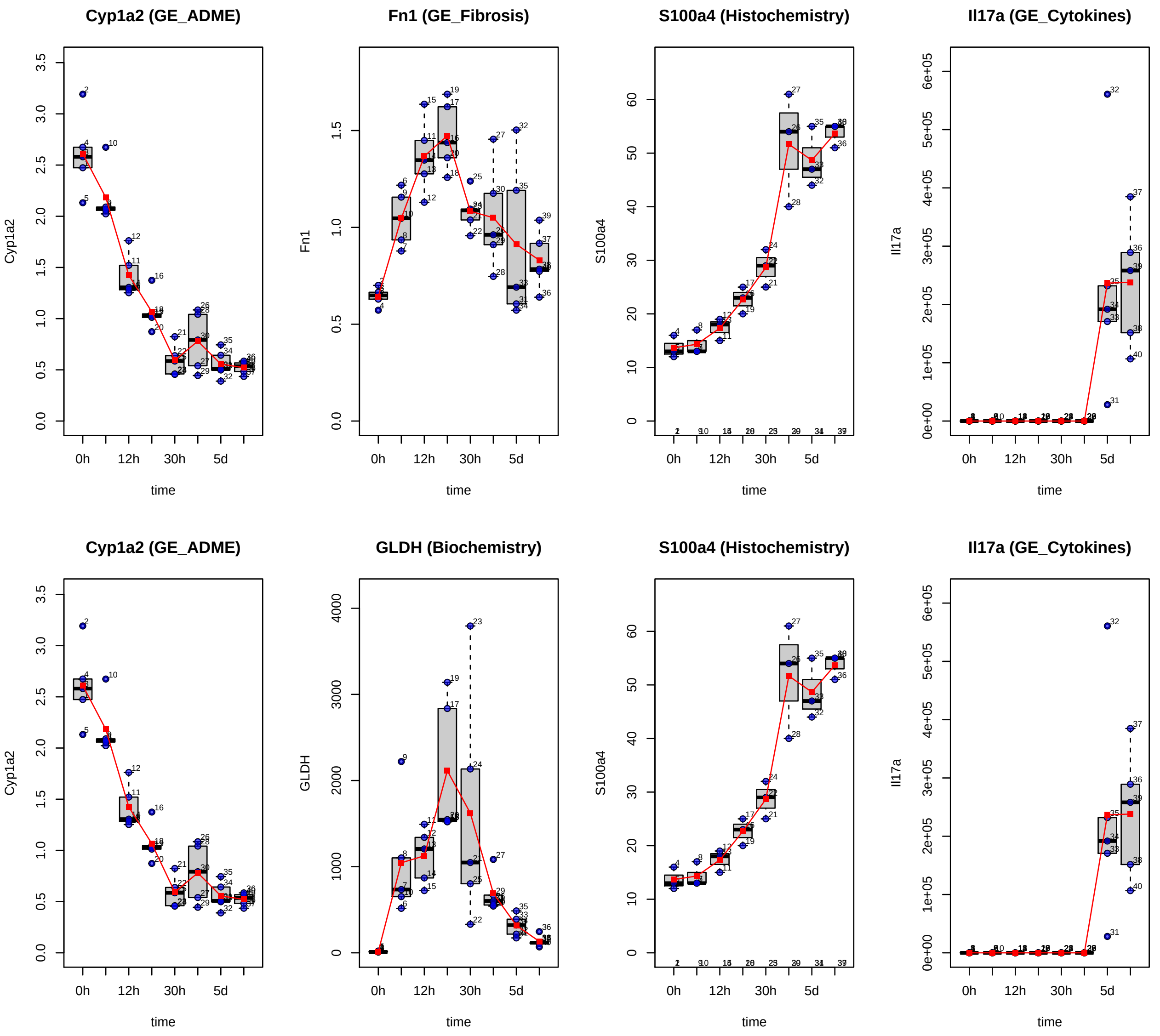

Figure: Best factor combination using all factors. Two alternative solutions for the single factor combinations.

```
plot_tree_representatives(gene.best)
```

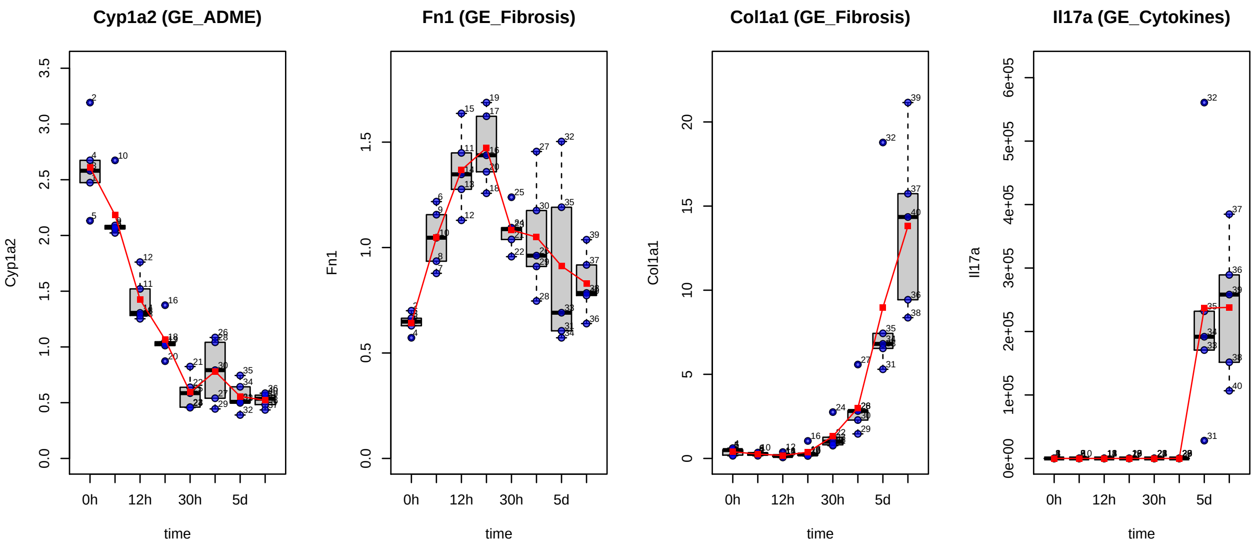

Figure: Best factor combination using transcript factors.

Prediction errors

Plot the distance distributions of single and double representatives and the best gene and representative trees.

```
# distance histogram
plot_tree_distance_dist <- function(){
  breaks <- seq(from=0, to=0.40, by=0.0125)
  hist(dist.single, freq=FALSE, breaks=breaks,
      main="Prediction error",
      xlab="RMSD(time.predicted, time.exp)",
      col=rgb(0.7,0.7,0.7, 1),
      ylim=c(0,15)
  )
  hist(dist.double, freq=FALSE, breaks=breaks,
       col=rgb(1,0,0, 0.5), add=TRUE)

  abline(v=dist.mean, col=rgb(0,0,1, 0.5), lwd=2)
  abline(v=dist.rep.best, col="black", lwd=2)
  abline(v=dist.gene.best, col="grey", lwd=2)

  legend("topright", legend=c("single factor", "double factor", "mean cluster"),
            col=c(rgb(0.7, 0.7, 0.7, 1),rgb(1, 0, 0, 0.5),rgb(0, 0, 1, 0.5)),
            bty="n", cex=1.0, pch=15)
}
# plot to file
pdf(file.path(resultsPath, "decision_tree", "tree_distance_distribution.pdf"),
    width=10, height=6, pointsize=14)
plot_tree_distance_dist()
invisible(dev.off())
```

```
plot_tree_distance_dist()
```

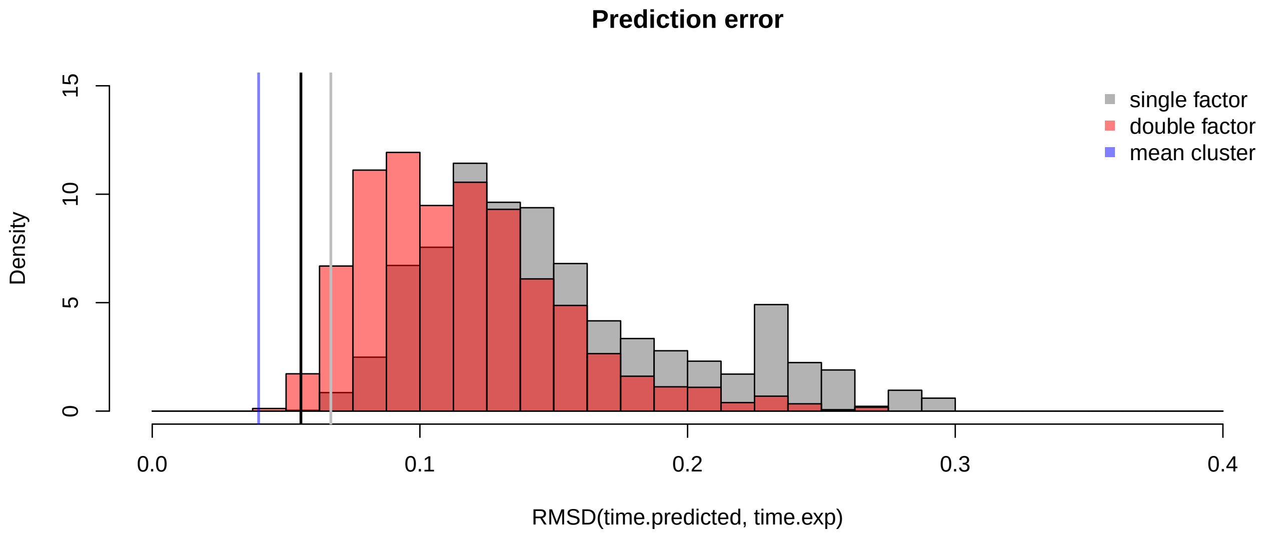

Figure: Prediction error of regression tree. Distribution of distances for single factor combinations, double factor combinations, mean cluster data, best gene factor combination, and best all factor combination. Adding additional factors to the prediction improves the predictions.

Predictive performance

Evaluation of the predictive performance of the regression trees by analysing which time points are predicted in which time classes. I.e. which experimental classes were predicted in which time classes of the regression tree for the mean cluster data (trainings data), single representative from each clusters and double representatives from each cluster.

```
# Predicted classes of the regression tree
node_levels <- levels(as.factor(pred.mean))
node_classes <- round(as.numeric(node_levels), digits=1)

# Plot of the predicted classes with the decision tree
plot_predicted_classes <- function(){
  # bar_colors <- brewer.pal(4, "Set3")
  bar_colors <- c(rgb(0.9,0.9,0.9),
                  rgb(0.7,0.7,0.7),
                  rgb(1,0,0, 0.5),
                  rgb(0,0,1, 0.5))
  par(mfrow=c(2,4))
  for (k in 1:Nt){
    # single factor predictions
    data <- as.vector(pred.single[, ((1:Nr)+Nr*(k-1))])
    tab.single <- table(factor(data, levels=node_levels))/length(data)
    # two factor predictions
    data <- as.vector(pred.double[, ((1:Nr)+Nr*(k-1))])
    tab.double <- table(factor(data, levels=node_levels))/length(data)
    # mean cluster predictions
    data <- as.vector(pred.mean[((1:Nr)+Nr*(k-1))])
    tab.mean <- table(factor(data, levels=node_levels))/length(data)
    # best single gene representative
    data <- as.vector(pred.gene.best[((1:Nr)+Nr*(k-1))])
    tab.gene.best <- table(factor(data, levels=node_levels))/length(data)
```

```
    # combined table
    tab <- rbind(tab.single, tab.double, tab.gene.best, tab.mean)
    colnames(tab) <- round(as.numeric(colnames(tab)), digits=1)

    # create the bar plot
    name <- sprintf("Time after BDL: %sh", levels(as.factor(BDLsamples$time))[k])
    barplot(tab, beside=TRUE,
            main=name,
            xlab="predicted time class [h]", ylab="fraction of predictions",
            ylim=c(0,1), col=bar_colors)
    if (k==1){
      legend("topright", legend=c("single factors", "double factors",
                                  "best single gene", "mean cluster"),
             col=bar_colors,
             bty="n", cex=1.0, pch=15)
    }
  }
  par(mfrow=c(1,1))
}
```

```
plot_predicted_classes()
```

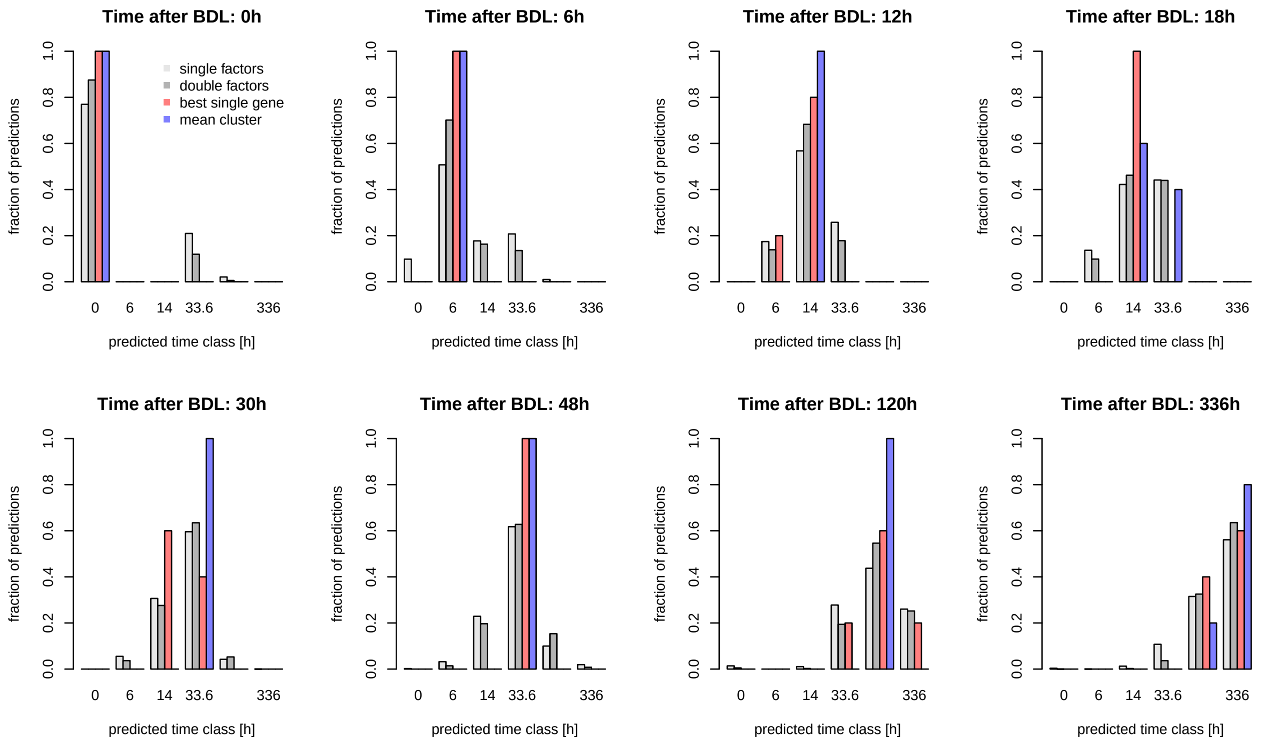

Figure: Prediction performance of regression tree.

```
# reverse bar plot
library(plyr)
library(reshape2)

# which experiments are predicted in which class
```

```
plot_predicted_classes2 <- function(){
  bar_colors <- c(rgb(1,1,1),
                  rgb(0.8,0.8,0.8),
                  rgb(1,0,0, 0.5),
                  rgb(0,0,1, 0.5))
  # single
  df <- data.frame(exp=rep(BDLsamples$time, Nsingle),
                 pre=as.vector(t(round(pred.single, digits=1))) )
  tmp <- count(df, c("exp", "pre"))
  tab.single <- acast(tmp, exp~pre, value.var="freq", fill=0)
  # double
  df <- data.frame(exp=rep(BDLsamples$time, Ndouble),
                 pre=as.vector(t(round(pred.double, digits=1))) )
  tmp <- count(df, c("exp", "pre"))
  tab.double <- acast(tmp, exp~pre, value.var="freq", fill=0)
  # best gene
  df <- data.frame(exp=rep(BDLsamples$time, 1),
                 pre=as.vector(t(round(pred.gene.best, digits=1))) )
  tmp <- count(df, c("exp", "pre"))
  tab.gene.best <- acast(tmp, exp~pre, value.var="freq", fill=0)
  # mean cluster
  df <- data.frame(exp=rep(BDLsamples$time, 1),
                 pre=as.vector(t(round(pred.mean, digits=1))) )
  tmp <- count(df, c("exp", "pre"))
  tab.mean <- acast(tmp, exp~pre, value.var="freq", fill=0)

  par(mfrow=c(2,3))
  for (k in 1:length(node_classes)){
    # combined table (normalized within each class)
    tab <- rbind(tab.single[,k]/sum(tab.single[,k]),
                 tab.double[,k]/sum(tab.double[,k]),
                 tab.gene.best[,k]/sum(tab.gene.best[,k]),
                 tab.mean[,k]/sum(tab.mean[,k]))

    # create the bar plot
    name <- sprintf("Predicted: %sh", node_classes[k])
    barplot(tab, beside=TRUE,
            main=name,
            xlab="time after BDL [h]", ylab="fraction",
            ylim=c(0,1), col=bar_colors)
    if (k==1){
      legend("topright", legend=c("single factors", "double factors",
                                  "best gene", "mean cluster"),
             col=bar_colors,
             bty="n", cex=1.0, pch=15)
    }
  }
  par(mfrow=c(1,1))
}

# barplot to file
pdf(file.path(resultsPath, "decision_tree", "predicted_classes2.pdf"),
    width=12, height=7.5, pointsize=14)
```

```r
plot_predicted_classes2()
invisible(dev.off())
```

```r
plot_predicted_classes2()
```

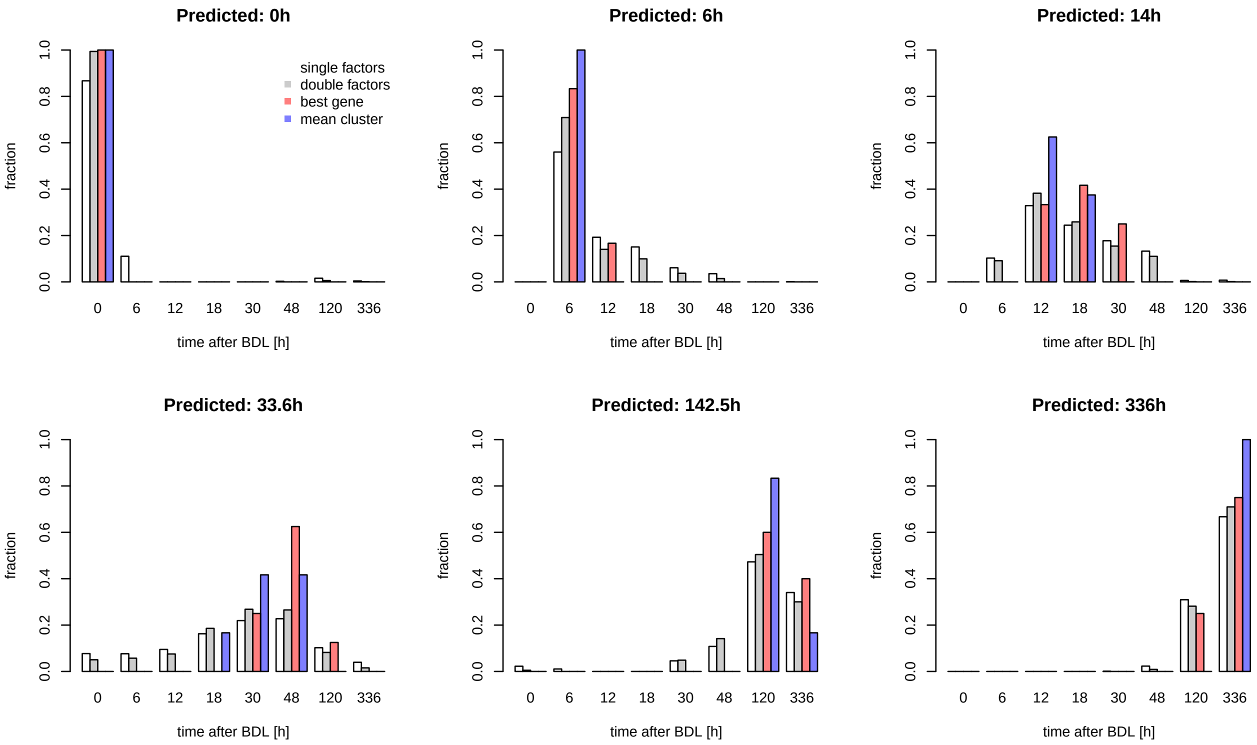

Figure: Prediction performance of regression tree.
